# Supplementary material for: Cryo-EM reveals the architecture of placental malaria VAR2CSA and provides molecular insight into chondroitin sulfate binding
Source: Nat Commun. 2021 May 19;12:2956. doi: 10.1038/s41467-021-23254-1 (PMC8134449; doi:10.1038/s41467-021-23254-1)
Supplement: Supplementary file 1 — Supplementary Information [file 41467_2021_23254_MOESM1_ESM.pdf]

Fig. S1: Resolution and structural flexibility of determined cryo-EM structures.

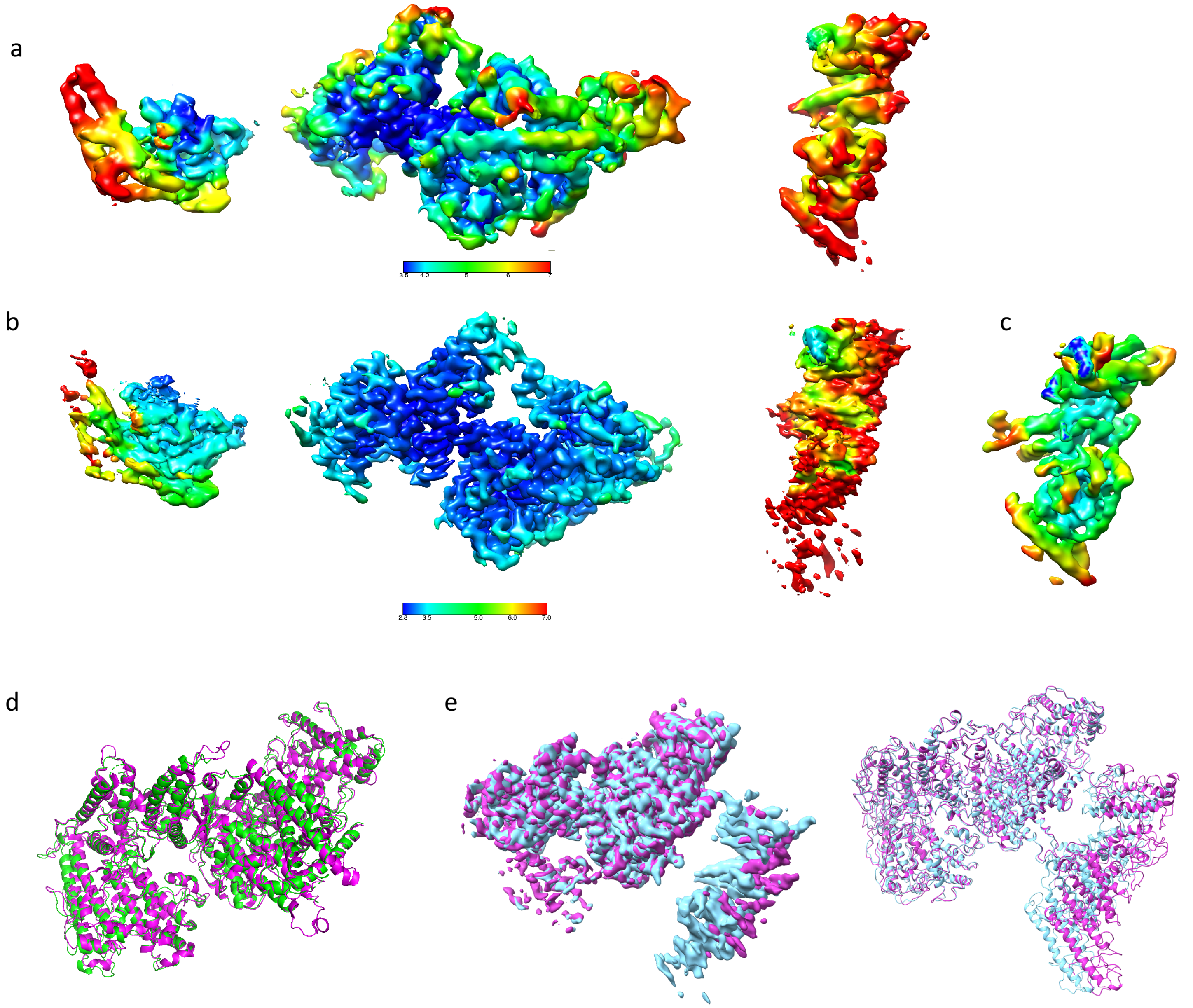

Fig. S1 a) Cryo-EM map of apo VAR2CSA, colored by local resolution. The DBL1 (left), core region ID1-ID3 (middle) and DBL5/6 (right) parts are shown separately. b) Cryo-EM map of VAR2CSA-plCS complex, colored by local resolution and shown as in a. The DBL1 and DBL5/6 parts have lower resolution compared to apo structure and the core region resolution has higher resolution. c) Improved DBL5/6 part of the map that is locally refined from a subset of the plCS particles (see supplement figure S6 for details), colored by local resolution with the same color code as b. d) Overlay of apo-structure (green) and plCS complex structure (purple) without DBL5/6, the rmsd between two structures is 0.73Å. e) Two independent maps/models calculated with two sub-fractions of plCS particles, alignment of the core structure shows displacement of DBL5/6.

Fig. S2: Quality of the cryo-EM density in selected functionally important regions

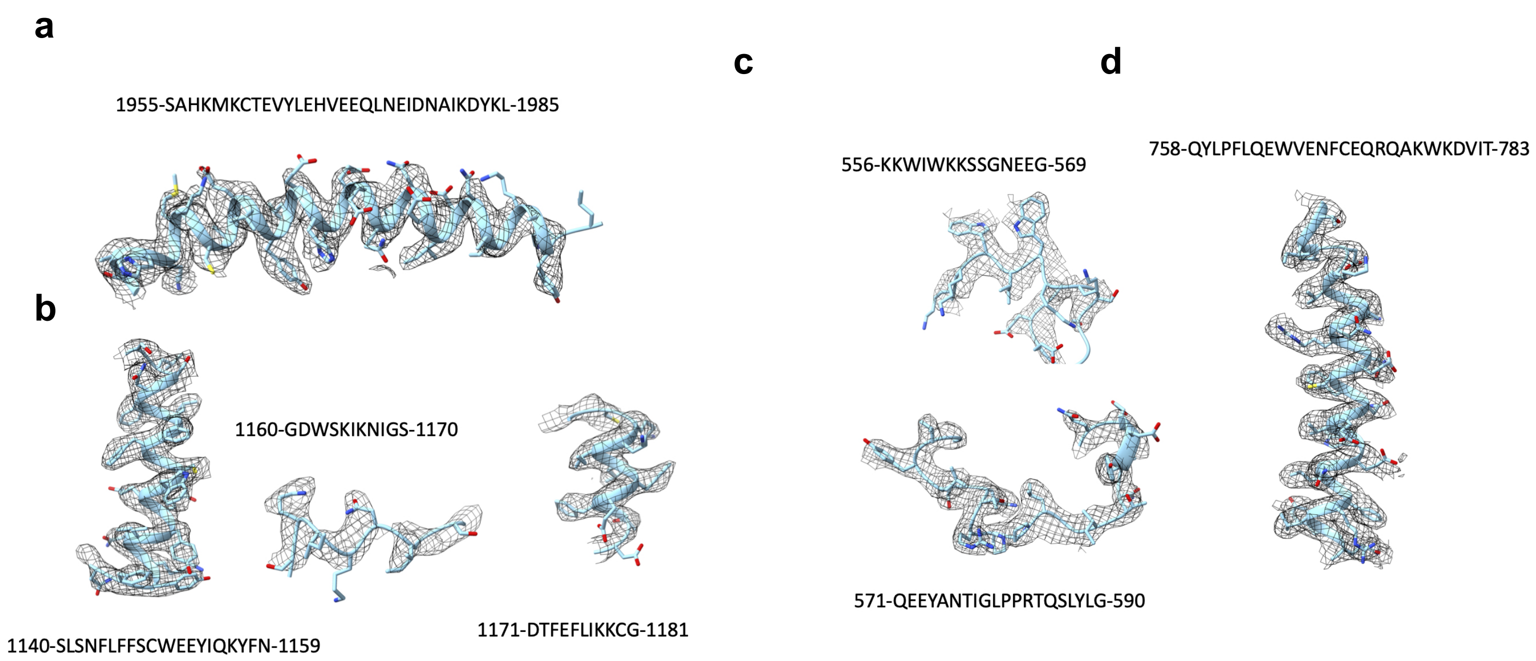

Fig. S2: a) ID3-helix (residue AA1955-1985). b) C-terminal part of ID2 which is interacting with ID3 α-helix (residue AA1140-1181). c) “WIW-motif”-loop in DBL2 (residue AA556-590). d) HB1 C-terminal half of α-helix in DBL2 involved in CS binding (residue AA758-783)

Fig. S3: Logo conservation of selected regions

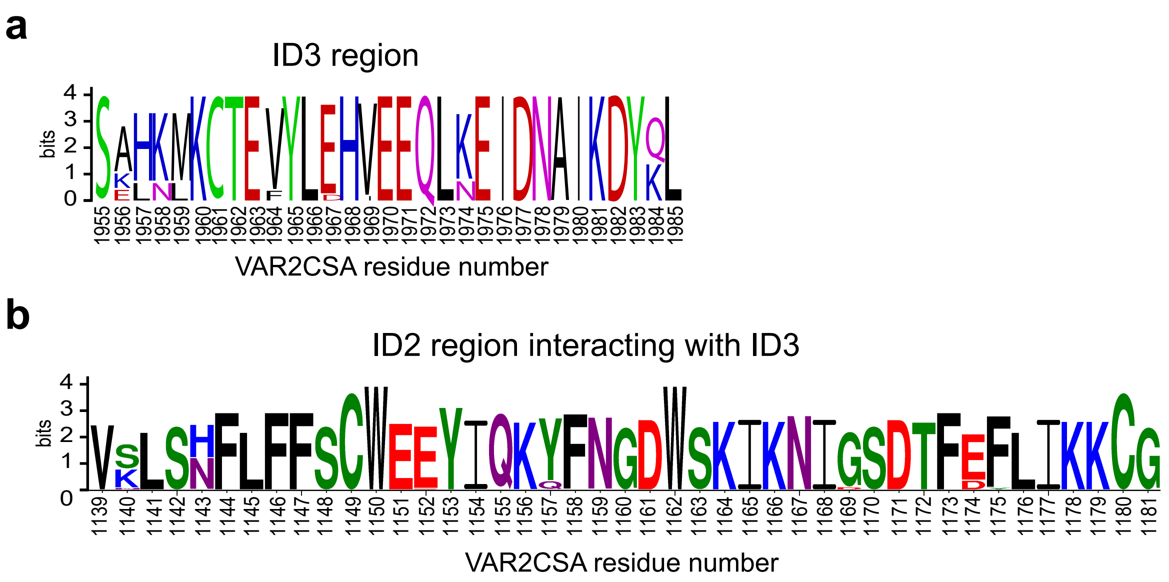

Fig. S3: a) LOGO conservation of ID3 region (residues AA1955-1985) in VAR2CSA subvariants. b) LOGO conservation of ID2 region interacting with ID3 (residues AA1139-1181).

Fig. S4: Structure of DBL3 and DBL4 in comparison with published crystal structures

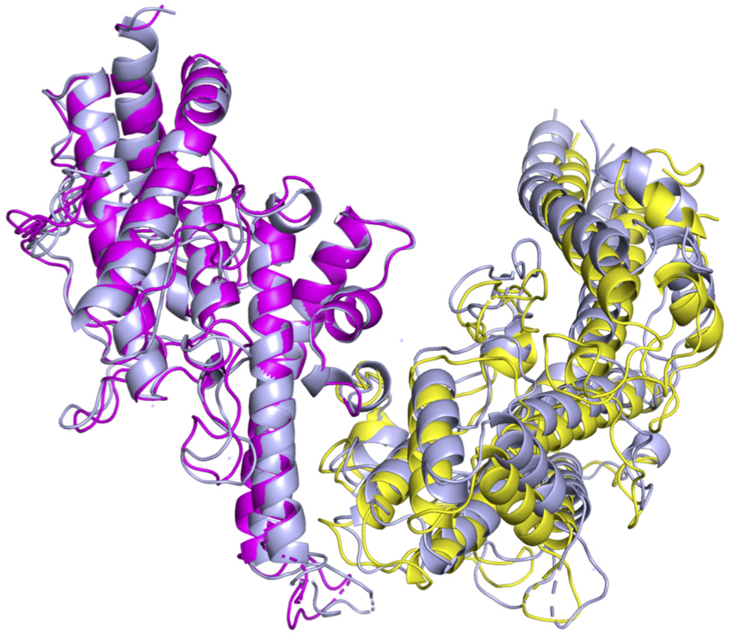

Fig. S4: The cryo-EM structure of the DBL3/4 domains compared to the equivalent crystal structure (PDB-ID 4P1T). The crystal structure is colored in gray and the cryo-EM structure of DBL3 in purple and DBL4 in yellow.

Fig. S5: Cryo-EM determination of VAR2CSA apo structure

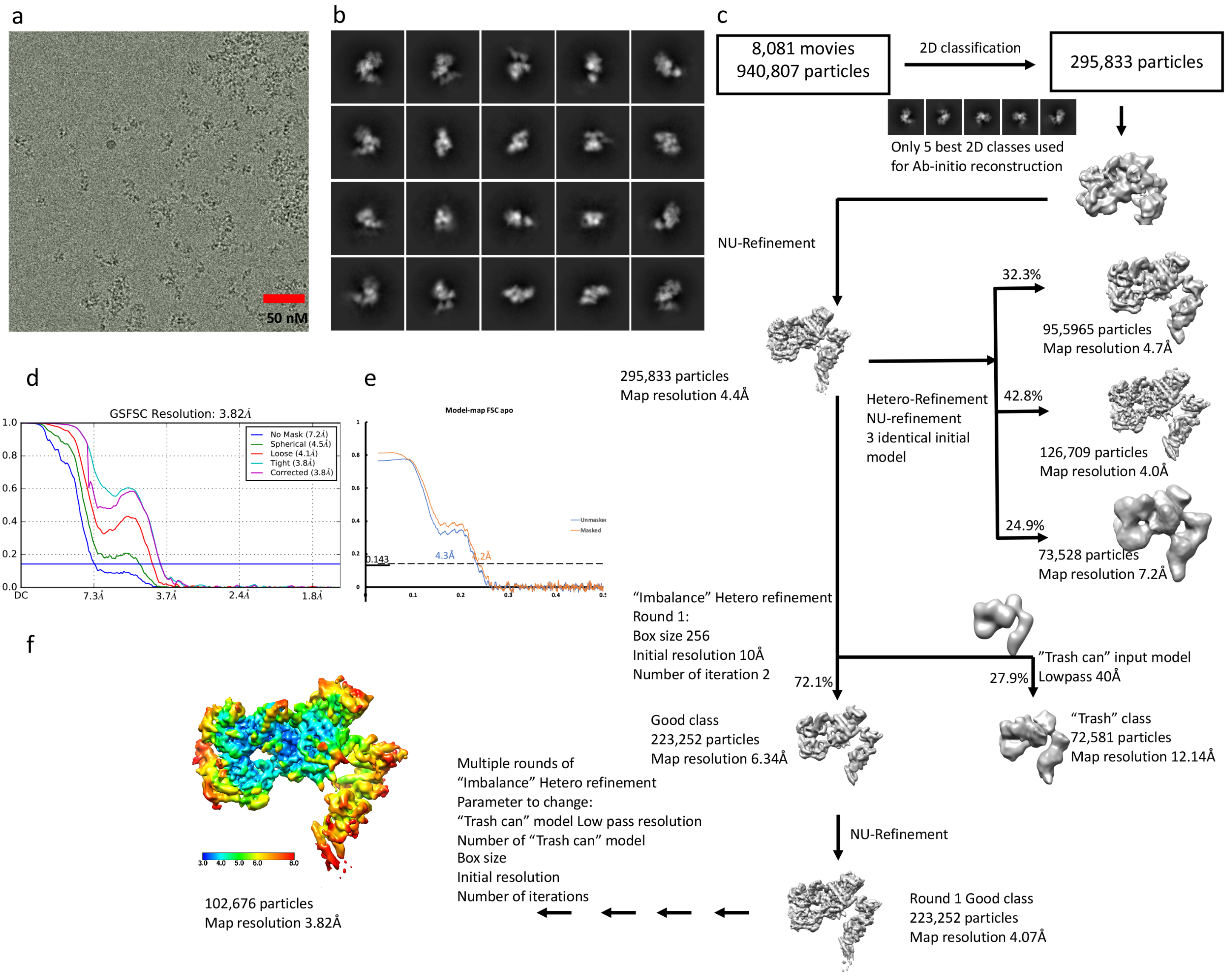

Fig S5: a) Representative micrograph of the grid used for structure determination. b) Representative 2D class averages with a box size of 36.6nm. c) Brief flowchart of the data processing. The Methods section and Table S1, S2 contains further details. d) Gold standard Fourier shell correlation (FSC) curve of the map. e) FSC curve of build model vs map. f) The final map colored by resolution.

Fig. S6: Cryo-EM determination of VAR2CSA pICS complex structure

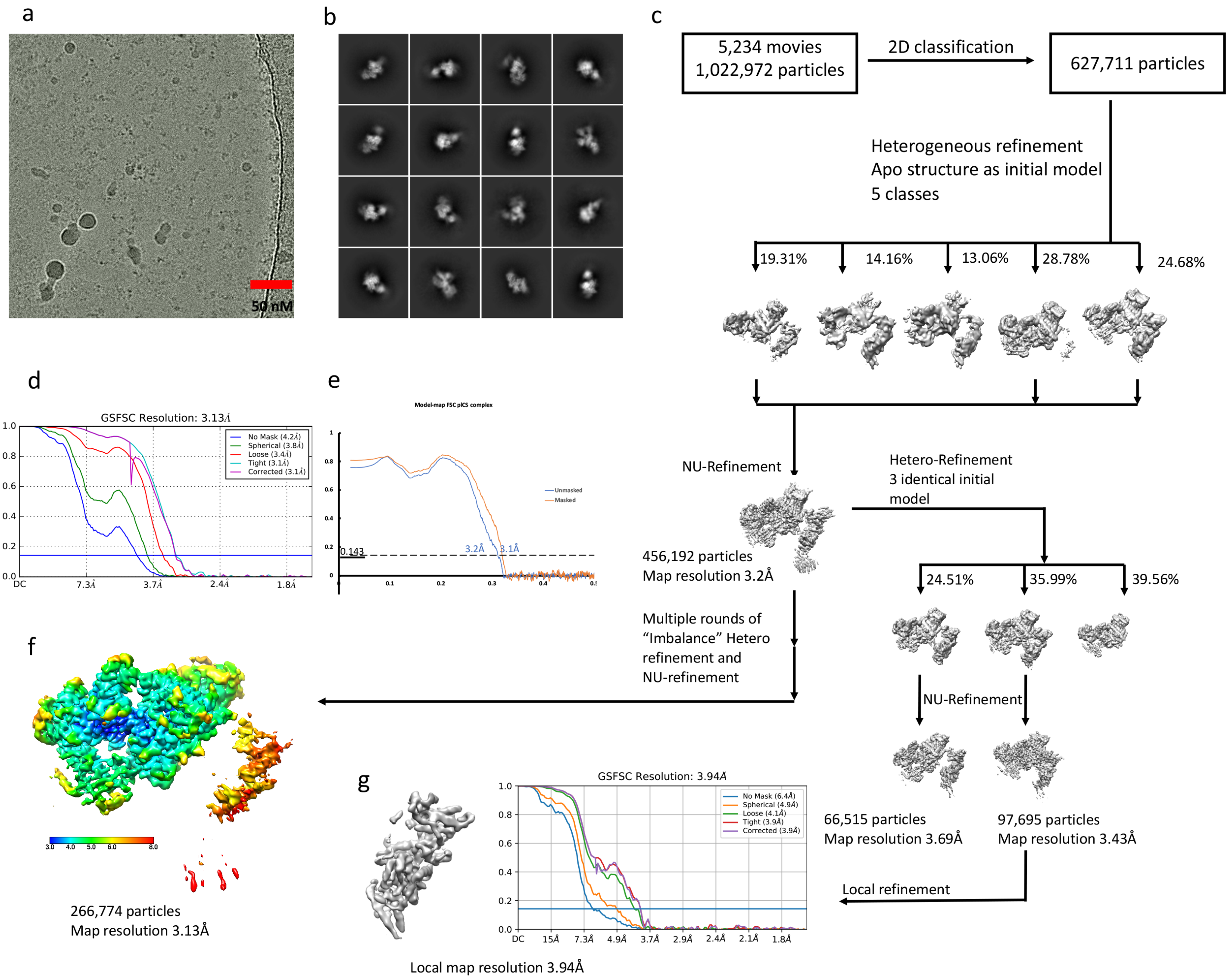

Fig. S6: a) Representative micrograph of the grid used for structure determination. b) Representative 2D class averages with a box size of 36.6nm. c) Brief flowchart of the data processing. The Methods section and Table S1, S2 contains further details. d) Gold standard Fourier shell correlation (FSC) curve of the map. e) FSC curve of build model vs map. f) The final map colored by resolution. g) Local refinement map on DBL5/6 and gold standard Fourier shell correlation (FSC) curve of the map.

Fig. S7: Logo of entire ecto domain

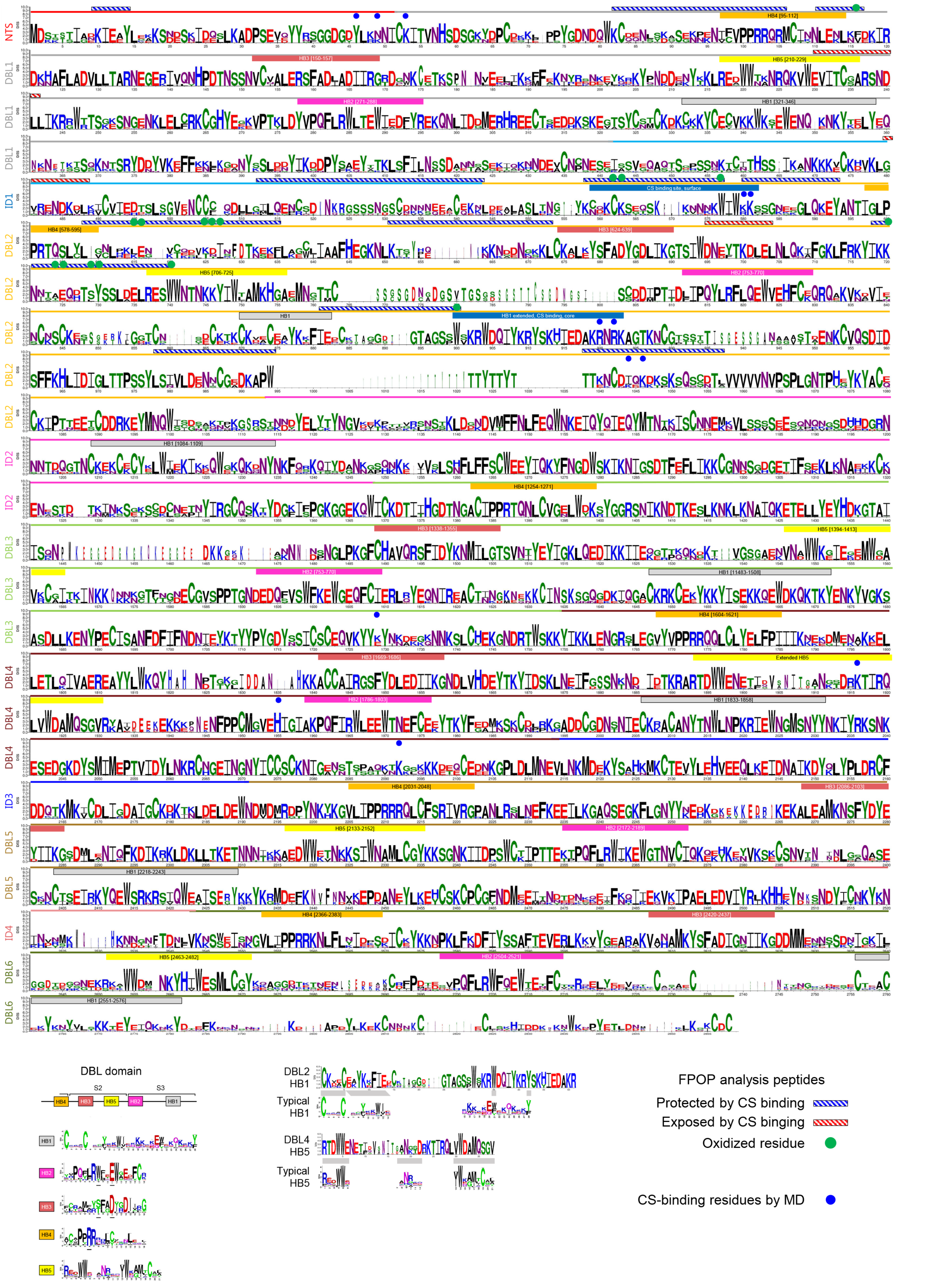

Fig S7. LOGO plot of VAR2CSA ectodomain. Domain and homology block borders are indicated above LOGO along with data from FPOP analysis of CS-protected/ exposed peptides and oxidized residues in the DBL1-ID2 protein, and CS-interacting residues predicted by Molecular Docking (MD). Small LOGO alignment below in the middle shows atypical HBs in VAR2CSA DBL2. Graphic HB LOGO legend modified from10

Fig. S8: FPOP peptides on structure

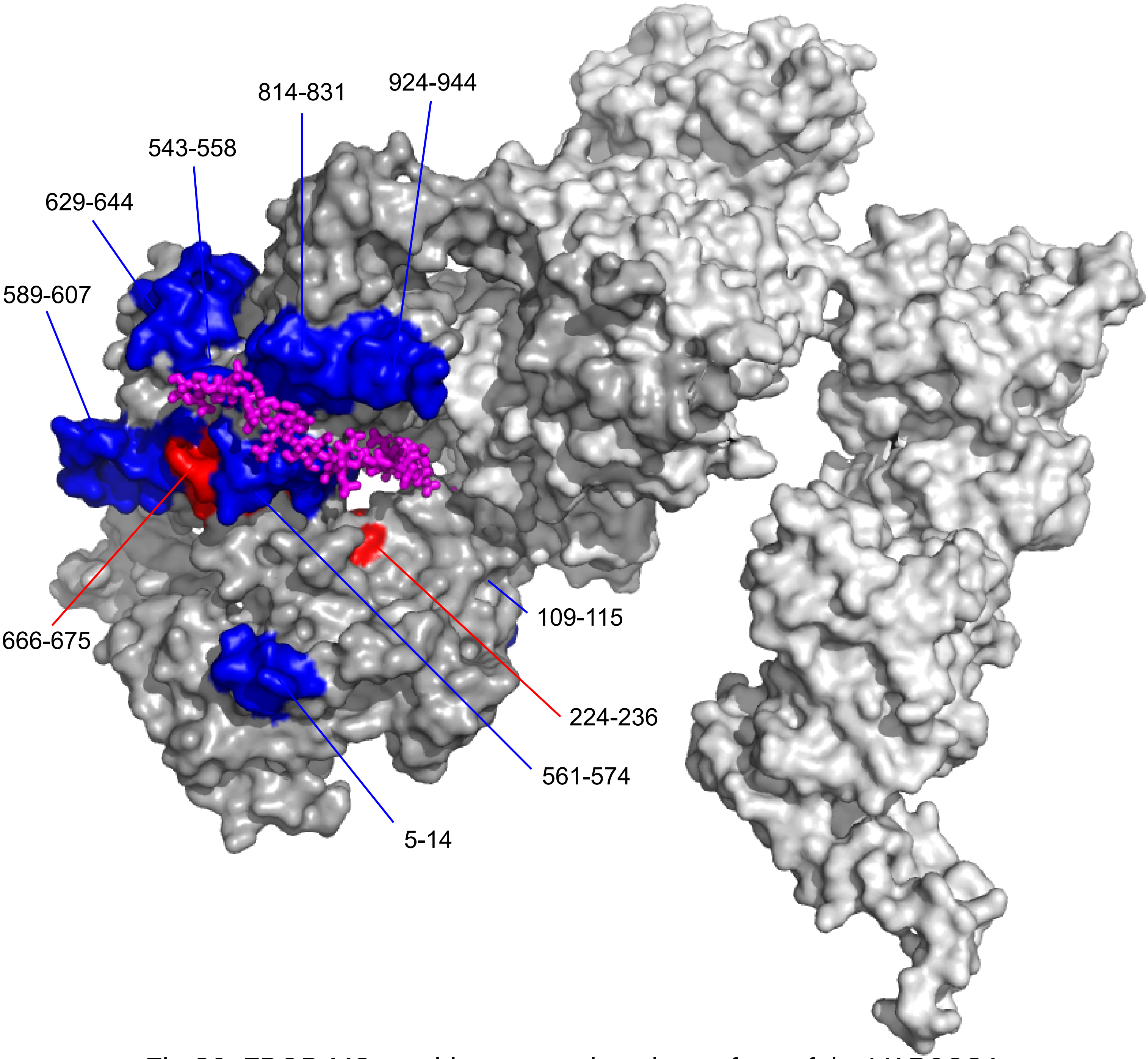

Fig S8: FPOP-MS peptides mapped on the surface of the VAR2CSA apo structure with docked CS 20-mer shown as magenta sticks. Blue patches are peptides which exhibit significant protection from oxidation upon binding to plCS. Red patches are peptides which exhibit significant exposure when binding plCS.

Fig S9: Extracted Ion Chromatograms of the VAR2CSA Peptides Oxidized by FPOP

Protected upon pICS binding:

A. **KPTKNKIEEY [z=2] Position 5-14:** Major oxidation products are K8 and Y14. Oxidation of K8 is significantly reduced upon pICS binding; oxidation of Y14 is unaffected by pICS binding.

Unoxidized peptide

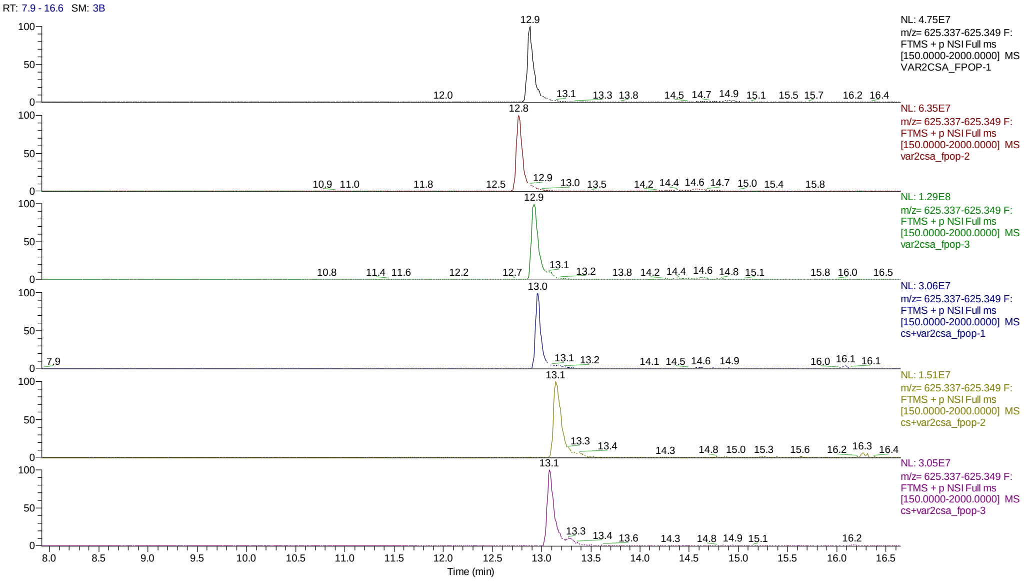

+1 ox

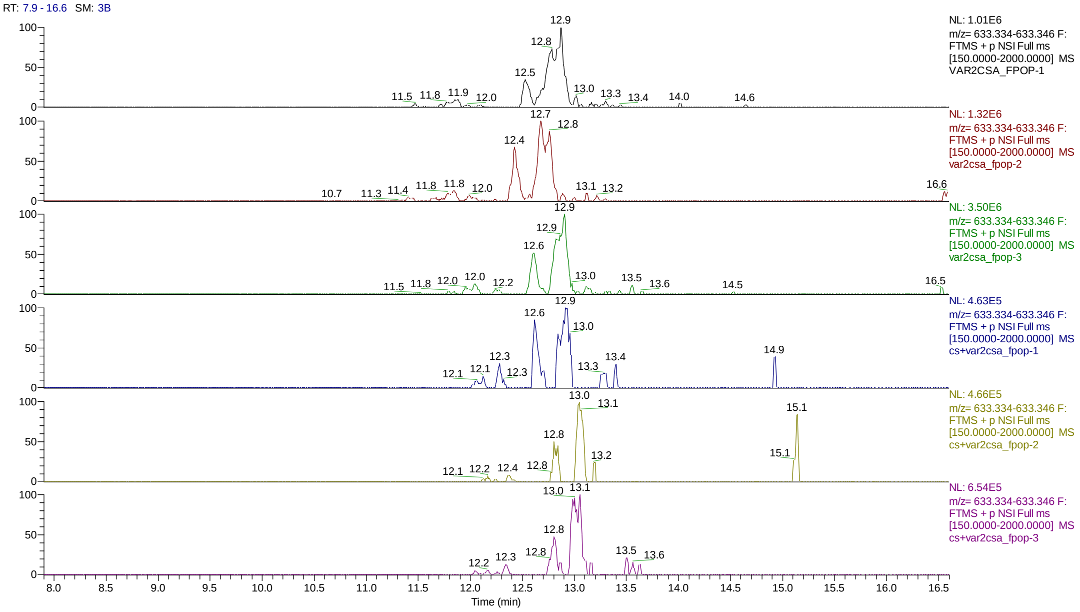

B. KCQQNSSDGSGKPENICVPPRRERL [z=4] Position 81-105: No amino acid level data, all oxidation products are reduced, C unlikely oxidation site due to lack of M+2O, M+3O signal

Unoxidized peptide

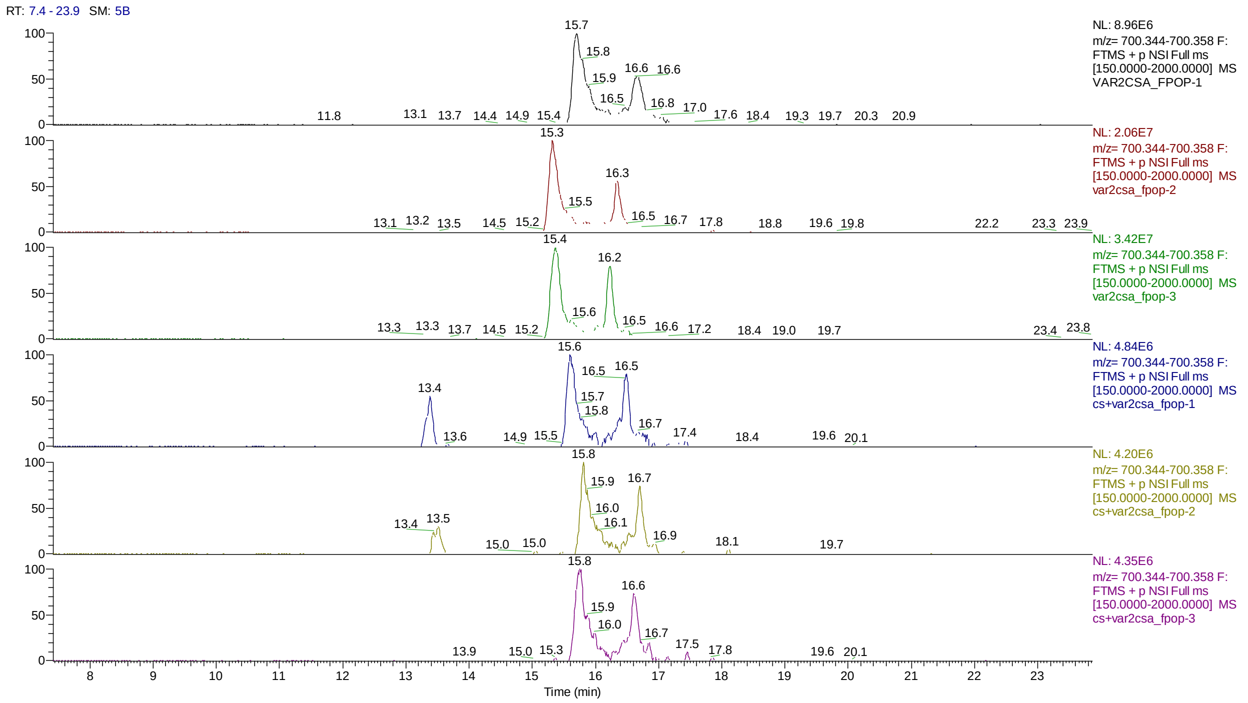

+1 ox

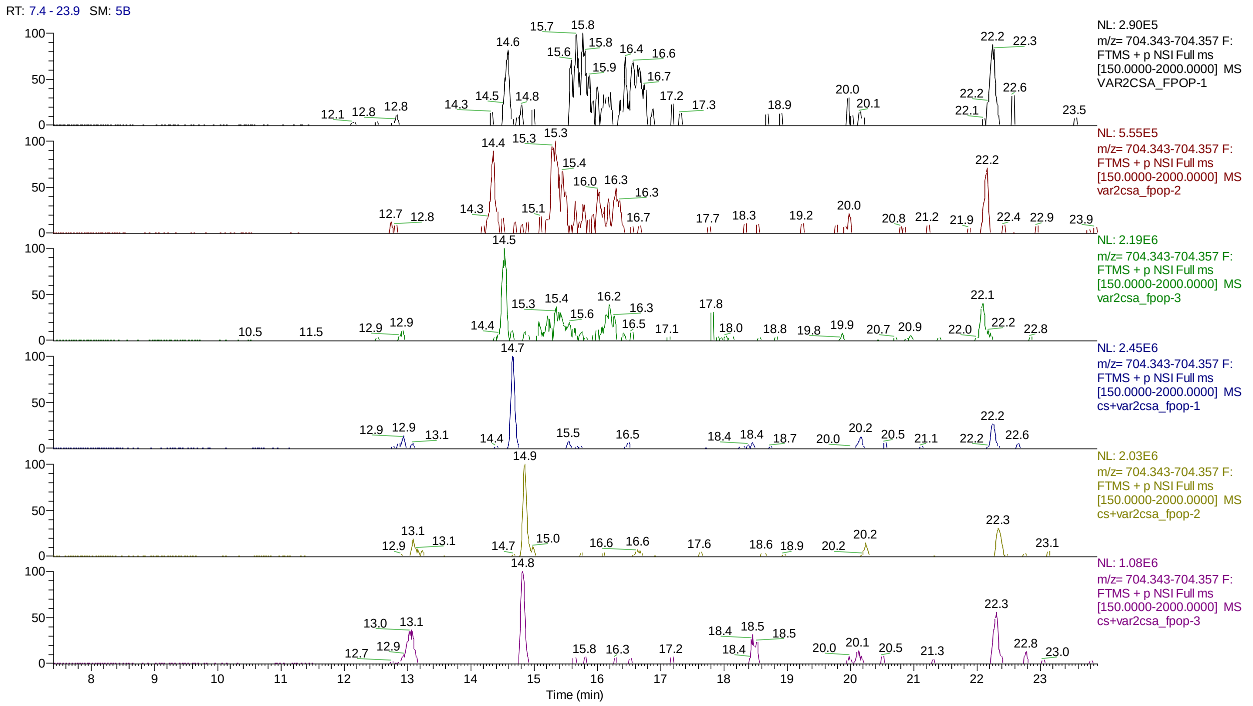

C. NLENLKF [z=2] Position 109-115: F115 is the major site of oxidation, and is significantly protected by pICS binding

Unoxidized peptide

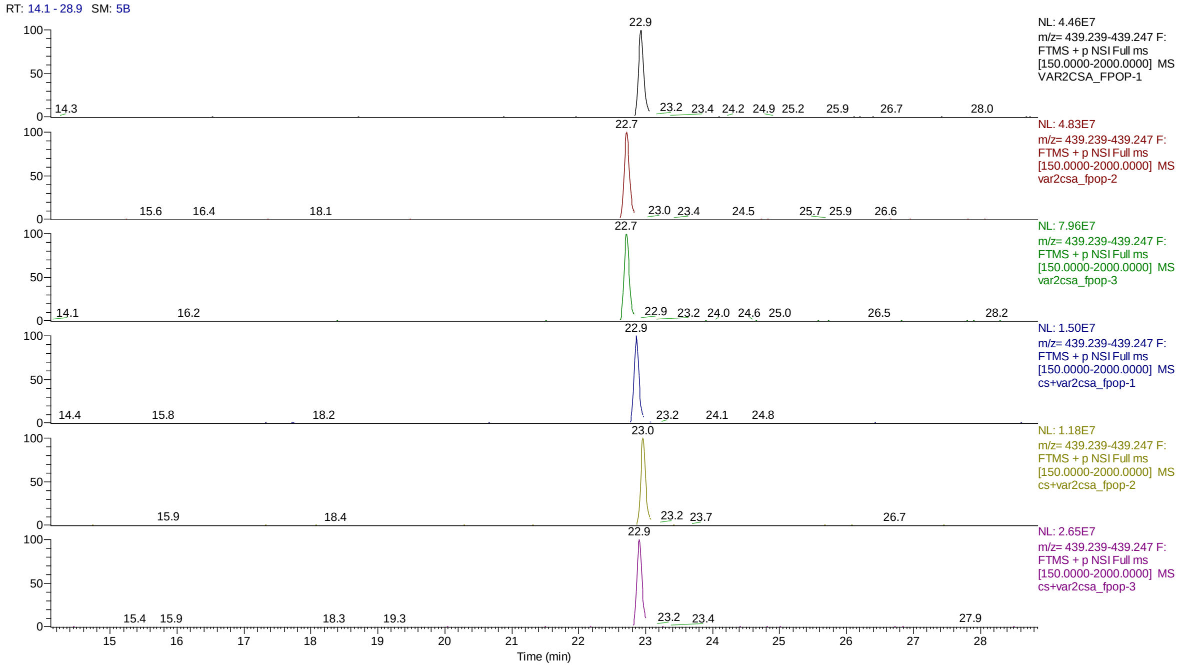

+1 ox

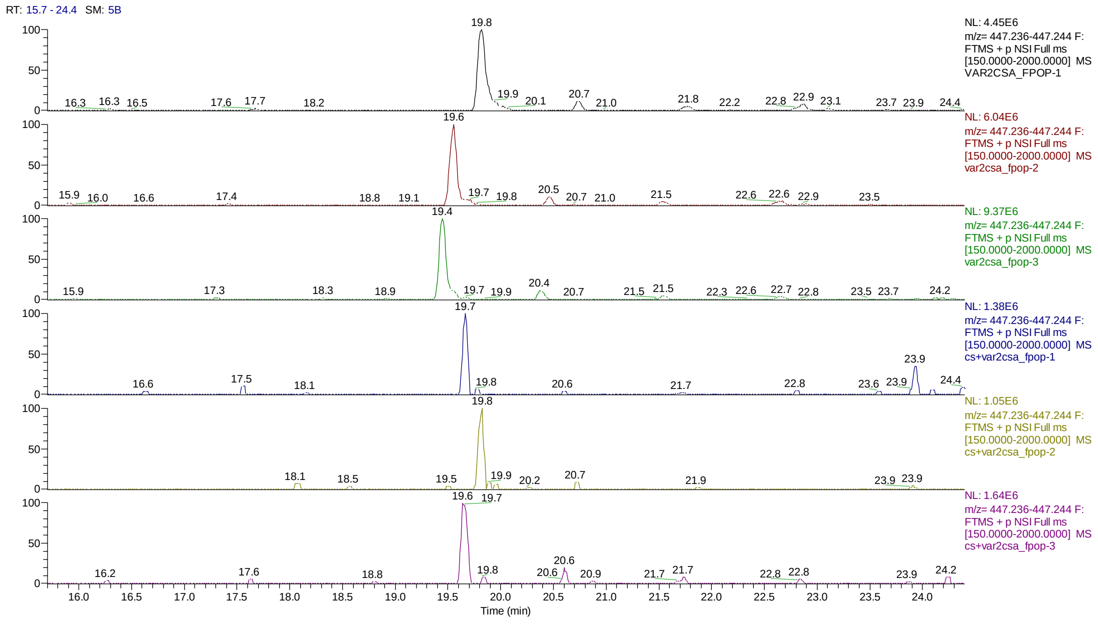

D. GILQENCSDNKRGSSSNDSCDNKNQDECQKKL [z=4] Position 500-531: No amino acid level data

Unoxidized peptide

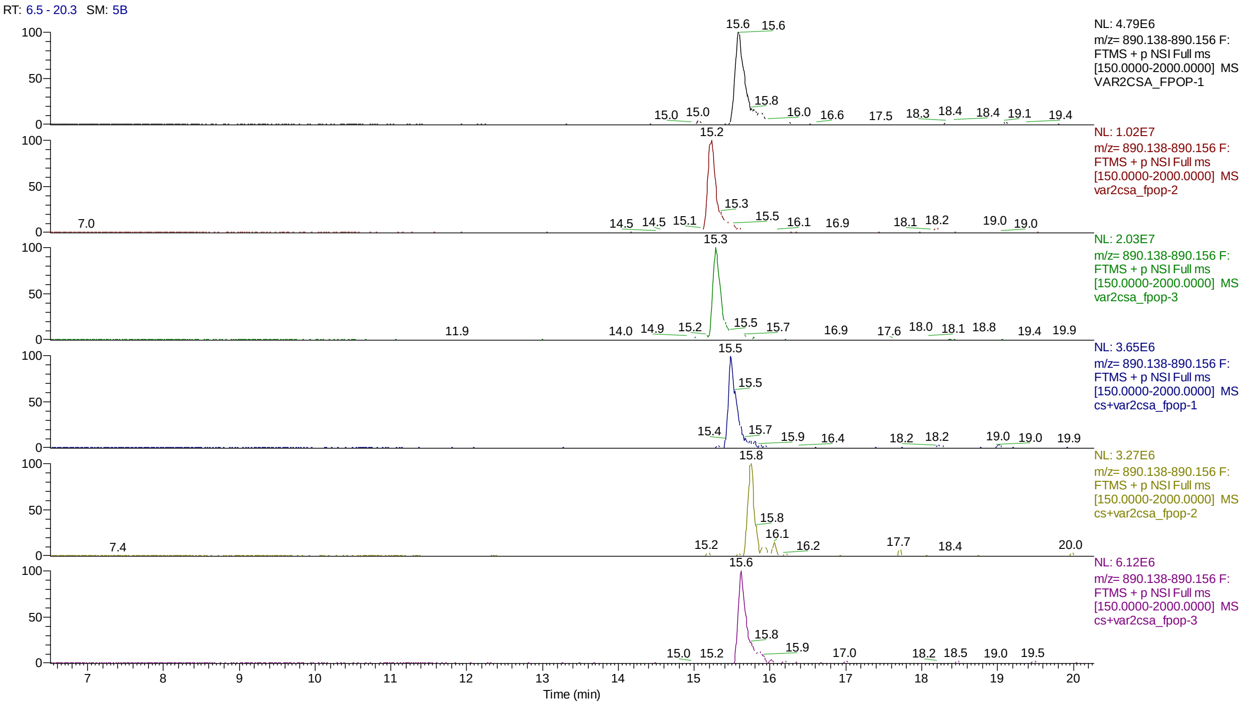

+1 ox

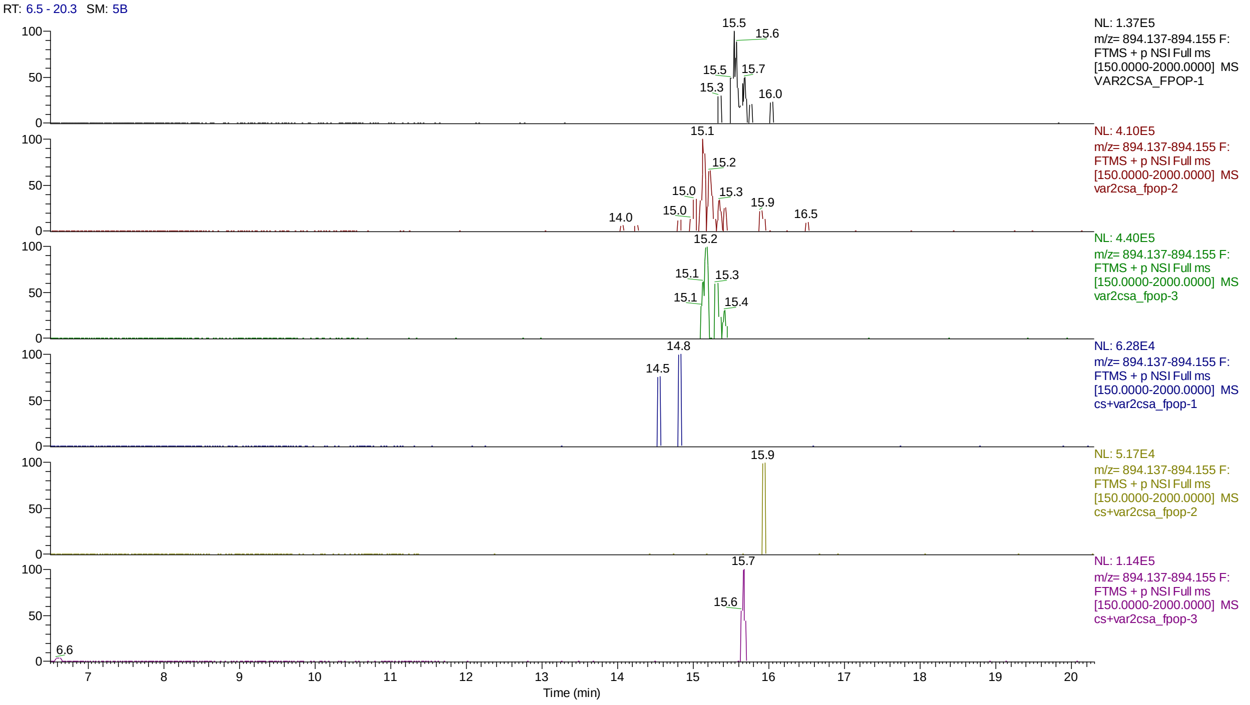

+2 ox

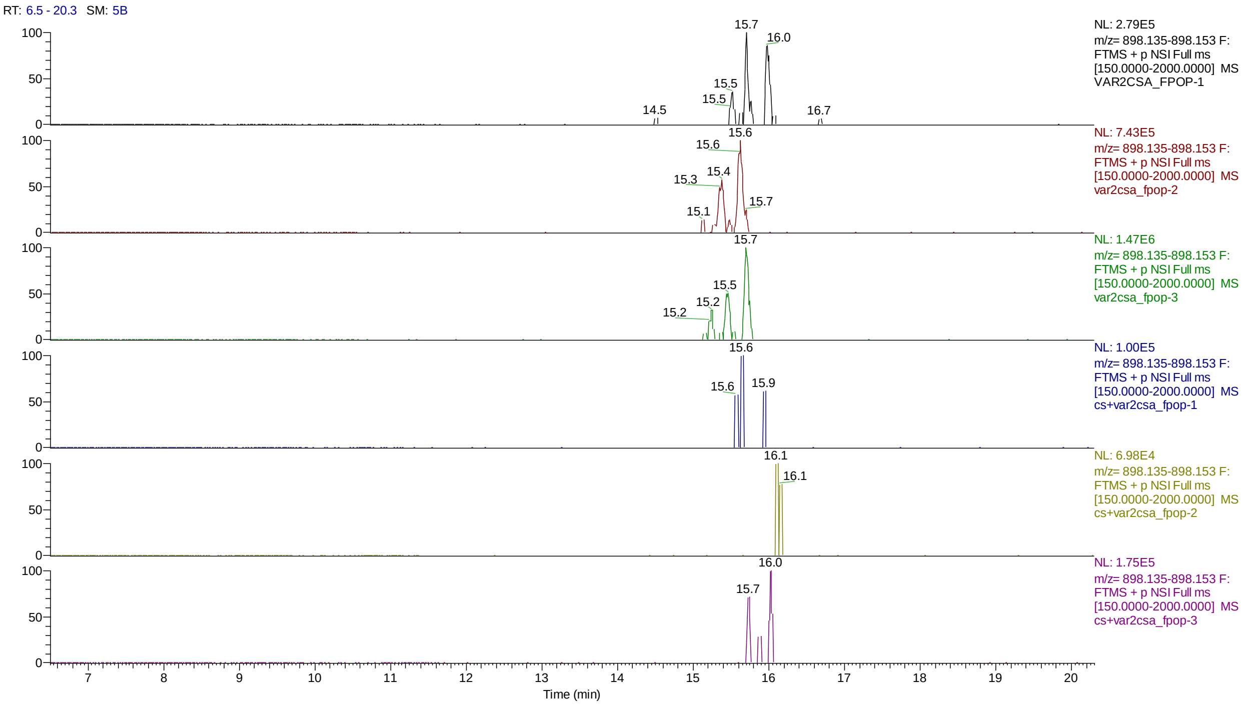

RT: 6.5 - 20.3 SM: 5B
NL: 2.79E5
m/z= 898.135-898.153 F: FTMS + p NSI Full ms [150.0000-2000.0000] MS VAR2CSA_FPOP-1
NL: 7.43E5
m/z= 898.135-898.153 F: FTMS + p NSI Full ms [150.0000-2000.0000] MS var2csa_fpop-2
NL: 1.47E6
m/z= 898.135-898.153 F: FTMS + p NSI Full ms [150.0000-2000.0000] MS var2csa_fpop-3
NL: 1.00E5
m/z= 898.135-898.153 F: FTMS + p NSI Full ms [150.0000-2000.0000] MS cs+var2csa_fpop-1
NL: 6.98E4
m/z= 898.135-898.153 F: FTMS + p NSI Full ms [150.0000-2000.0000] MS cs+var2csa_fpop-2
NL: 1.75E5
m/z= 898.135-898.153 F: FTMS + p NSI Full ms [150.0000-2000.0000] MS cs+var2csa_fpop-3
Time (min)

E. KCDKCKSGTSRSKKKW [z=3] Position 543-558: Major oxidation sites identified as W558 and either C547 and/or K548. C oxidation likely due to presence of M+2O and M+3O signal. All oxidation products reduced upon pICS binding.

Unoxidized peptide

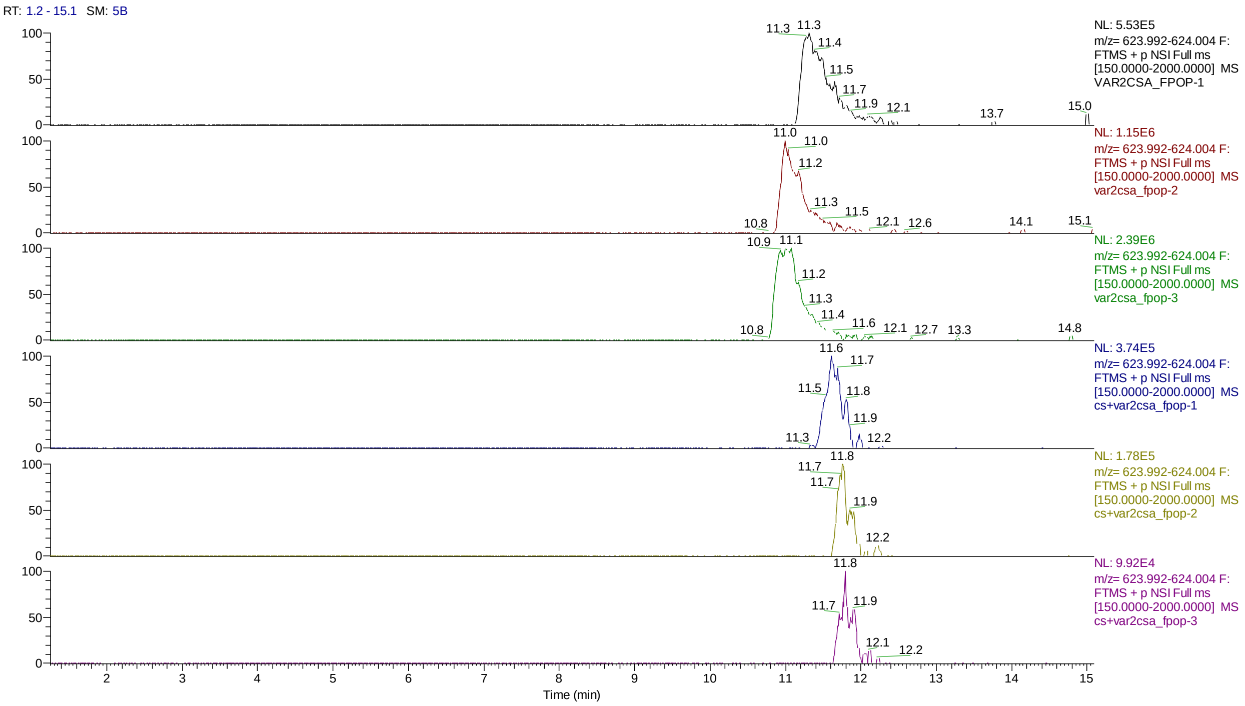

+1 ox

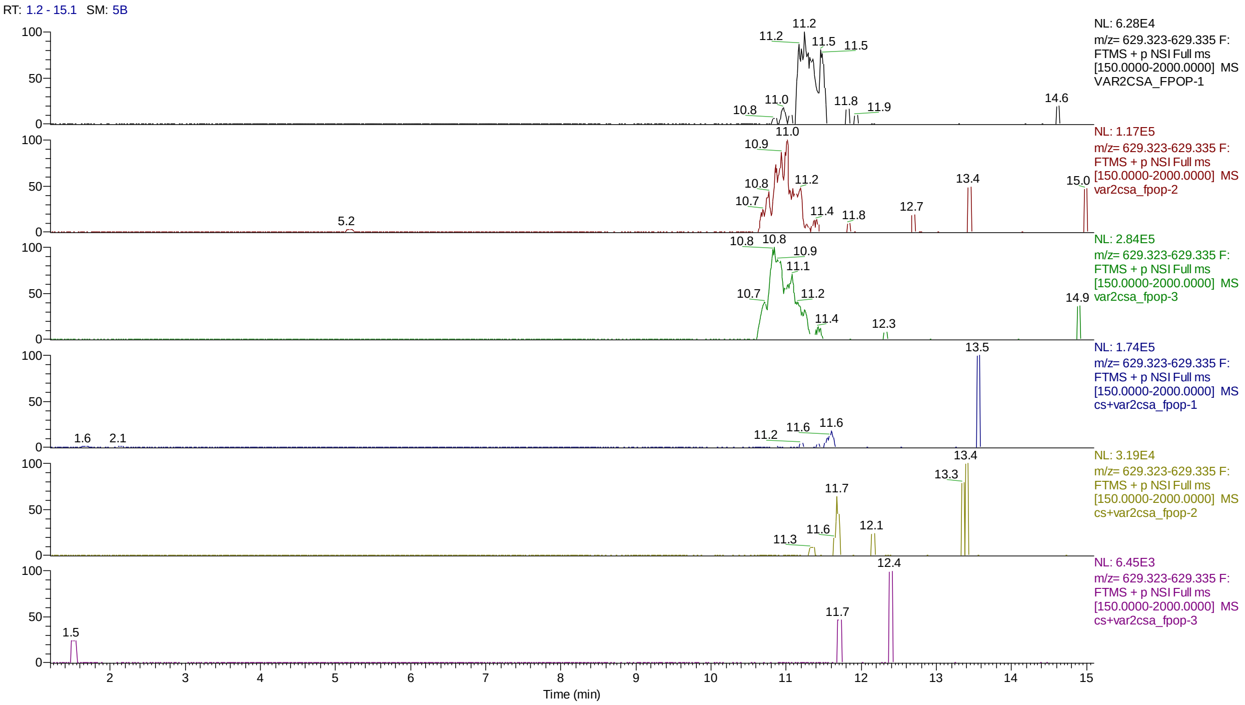

+2 ox

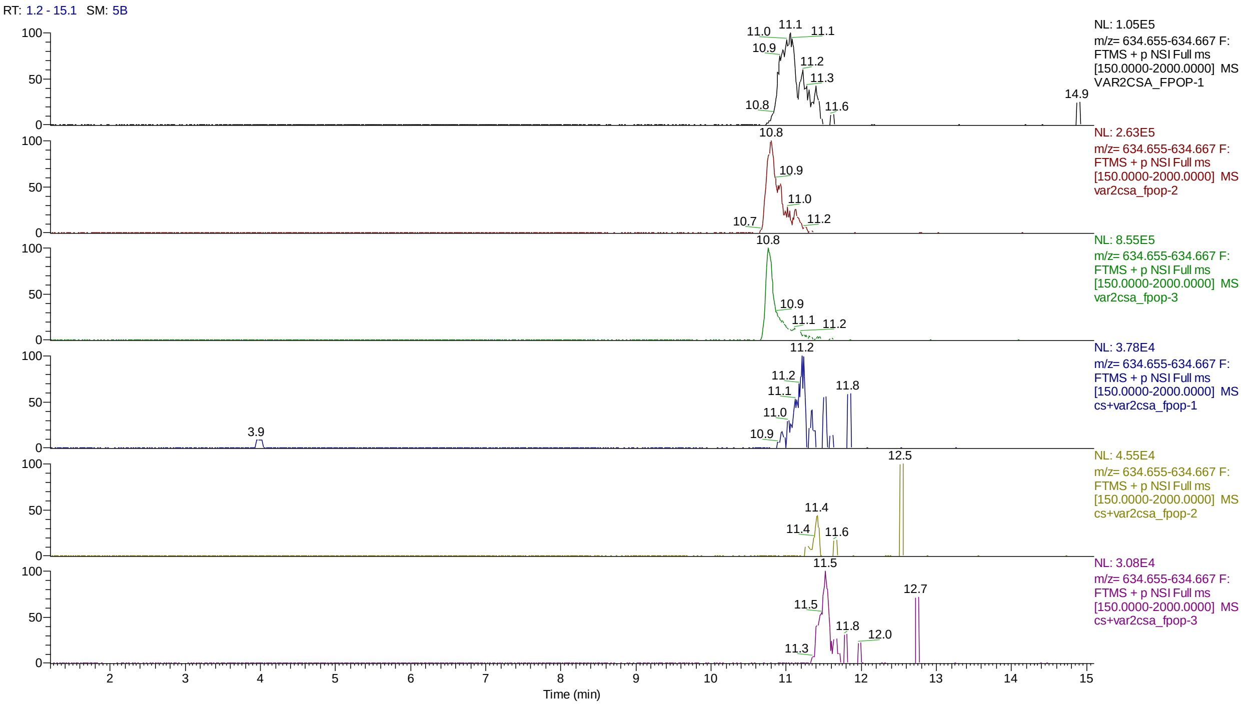
RT: 1.2 - 15.1 SM: 5B
NL: 1.05E5
m/z= 634.655-634.667 F: FTMS + p NSI Full ms [150.0000-2000.0000] MS VAR2CSA_FPOP-1
NL: 2.63E5
m/z= 634.655-634.667 F: FTMS + p NSI Full ms [150.0000-2000.0000] MS var2csa_fpop-2
NL: 8.55E5
m/z= 634.655-634.667 F: FTMS + p NSI Full ms [150.0000-2000.0000] MS var2csa_fpop-3
NL: 3.78E4
m/z= 634.655-634.667 F: FTMS + p NSI Full ms [150.0000-2000.0000] MS cs+var2csa_fpop-1
NL: 4.55E4
m/z= 634.655-634.667 F: FTMS + p NSI Full ms [150.0000-2000.0000] MS cs+var2csa_fpop-2
NL: 3.08E4
m/z= 634.655-634.667 F: FTMS + p NSI Full ms [150.0000-2000.0000] MS cs+var2csa_fpop-3
Time (min)

+3 ox

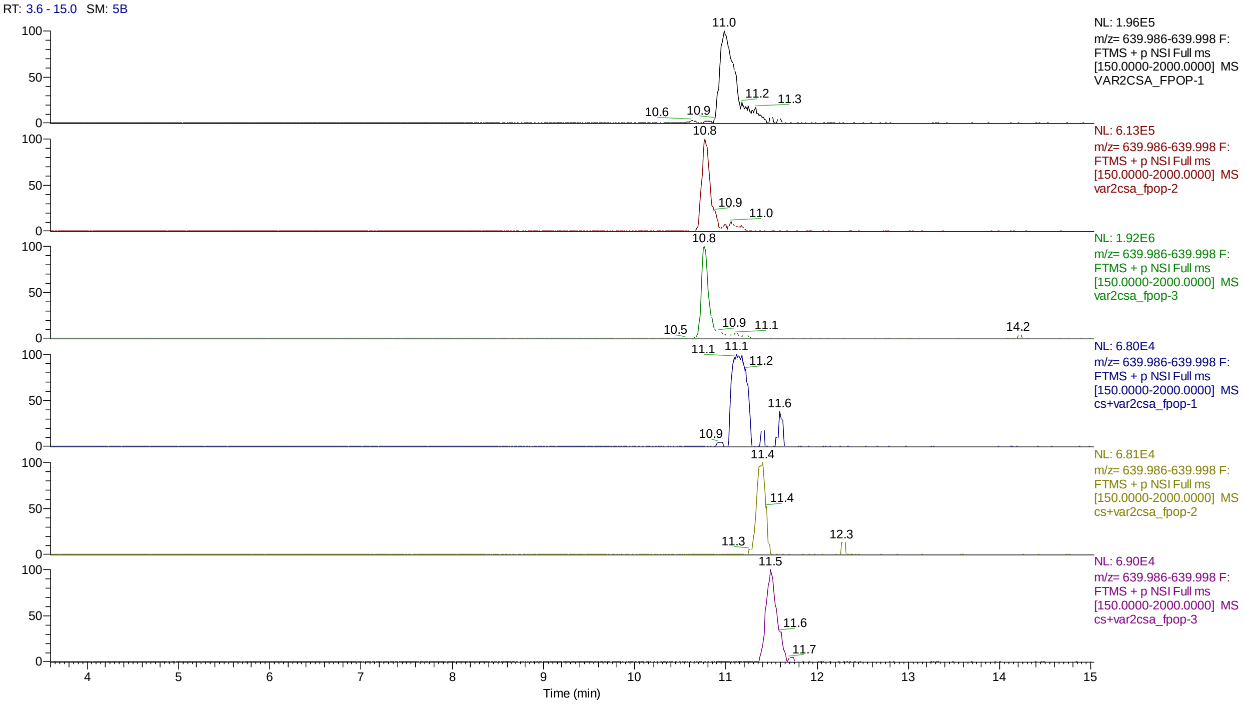
RT: 3.6 - 15.0 SM: 5B
NL: 1.96E5
m/z= 639.986-639.998 F: FTMS + p NSI Full ms [150.0000-2000.0000] MS VAR2CSA_FPOP-1
NL: 6.13E5
m/z= 639.986-639.998 F: FTMS + p NSI Full ms [150.0000-2000.0000] MS var2csa_fpop-2
NL: 1.92E6
m/z= 639.986-639.998 F: FTMS + p NSI Full ms [150.0000-2000.0000] MS var2csa_fpop-3
NL: 6.80E4
m/z= 639.986-639.998 F: FTMS + p NSI Full ms [150.0000-2000.0000] MS cs+var2csa_fpop-1
NL: 6.81E4
m/z= 639.986-639.998 F: FTMS + p NSI Full ms [150.0000-2000.0000] MS cs+var2csa_fpop-2
NL: 6.90E4
m/z= 639.986-639.998 F: FTMS + p NSI Full ms [150.0000-2000.0000] MS cs+var2csa_fpop-3
Time (min)

F. KKSSGNEEGLQEEY [z=2] Position 561-574: no amino acid level data; both major oxidation products are significantly reduced upon pICS binding

Unoxidized peptide

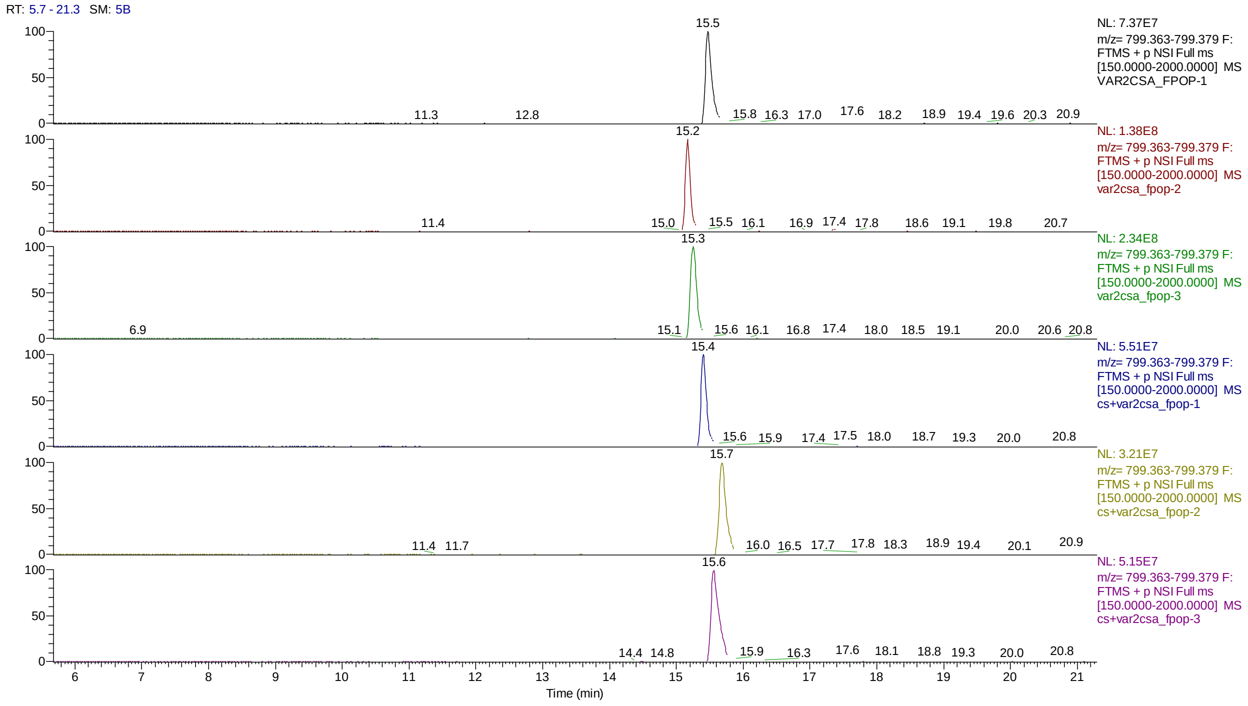

+1 ox

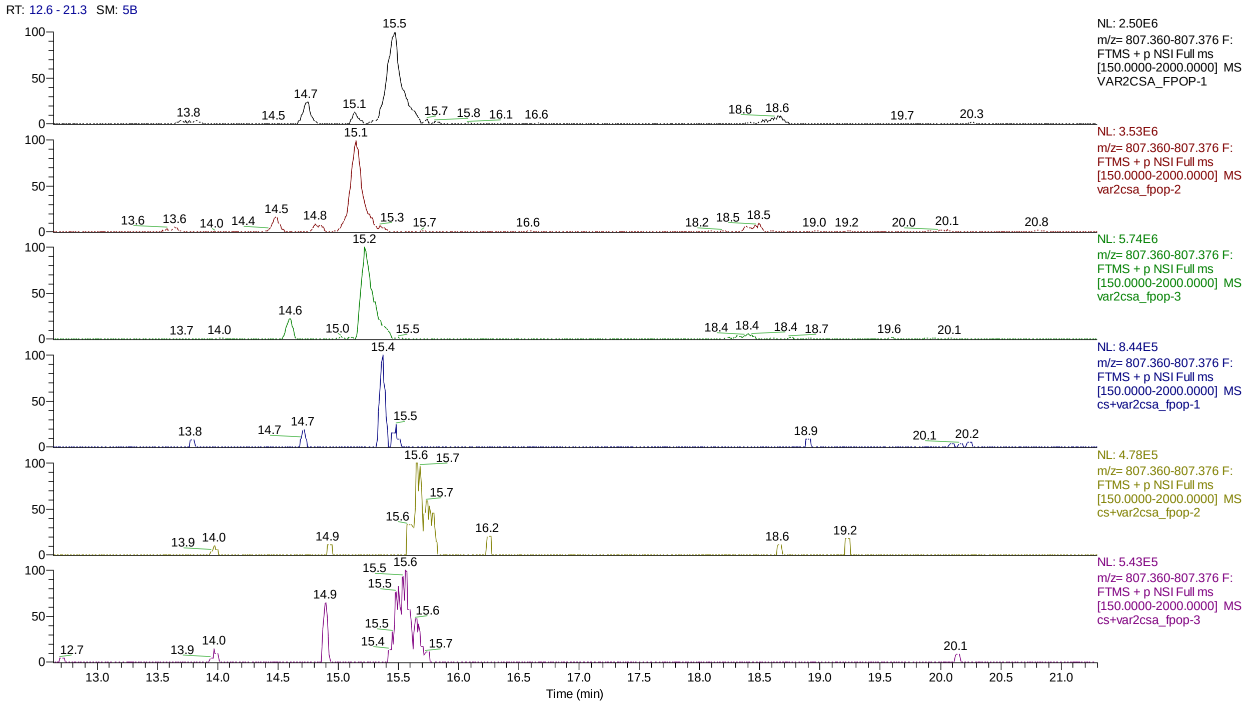

G. LGNLPKLENVCEDVKDINF [z=3] Position 589-607: Three major oxidation products, all of which are reduced upon pICS binding: K603; L595 and/or E596; and D604 and/or I605

Unoxidized peptide

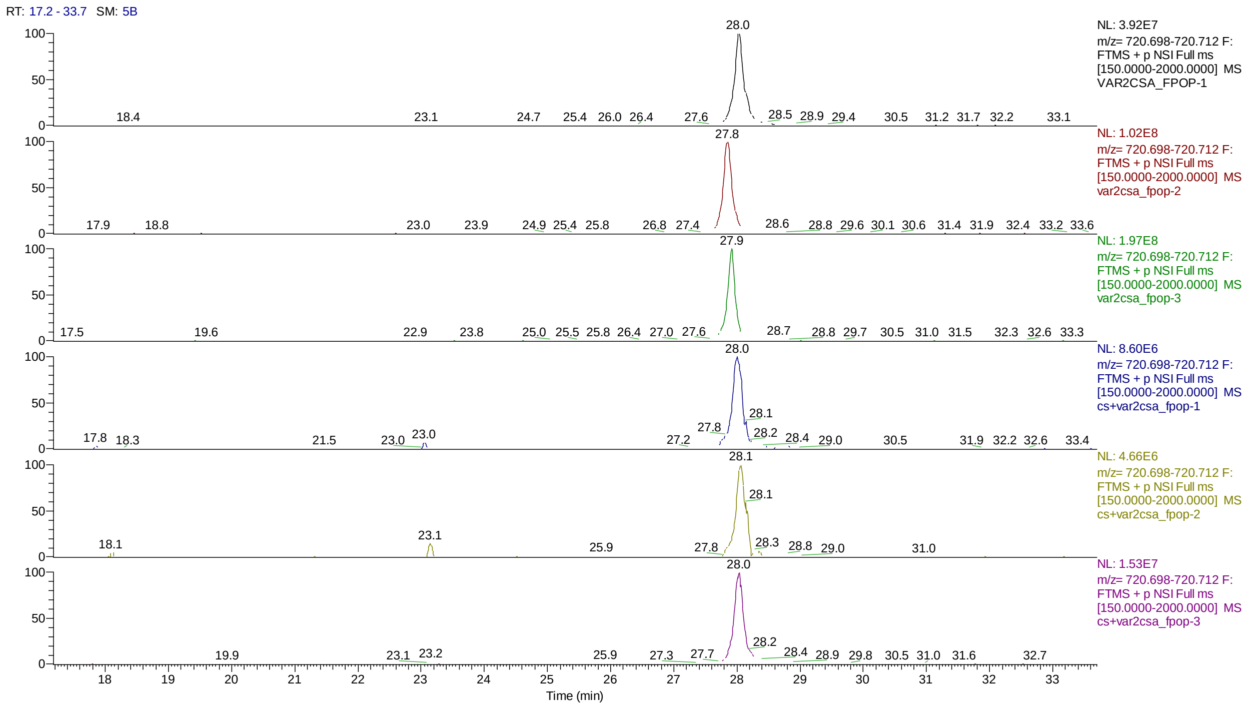

+1 ox

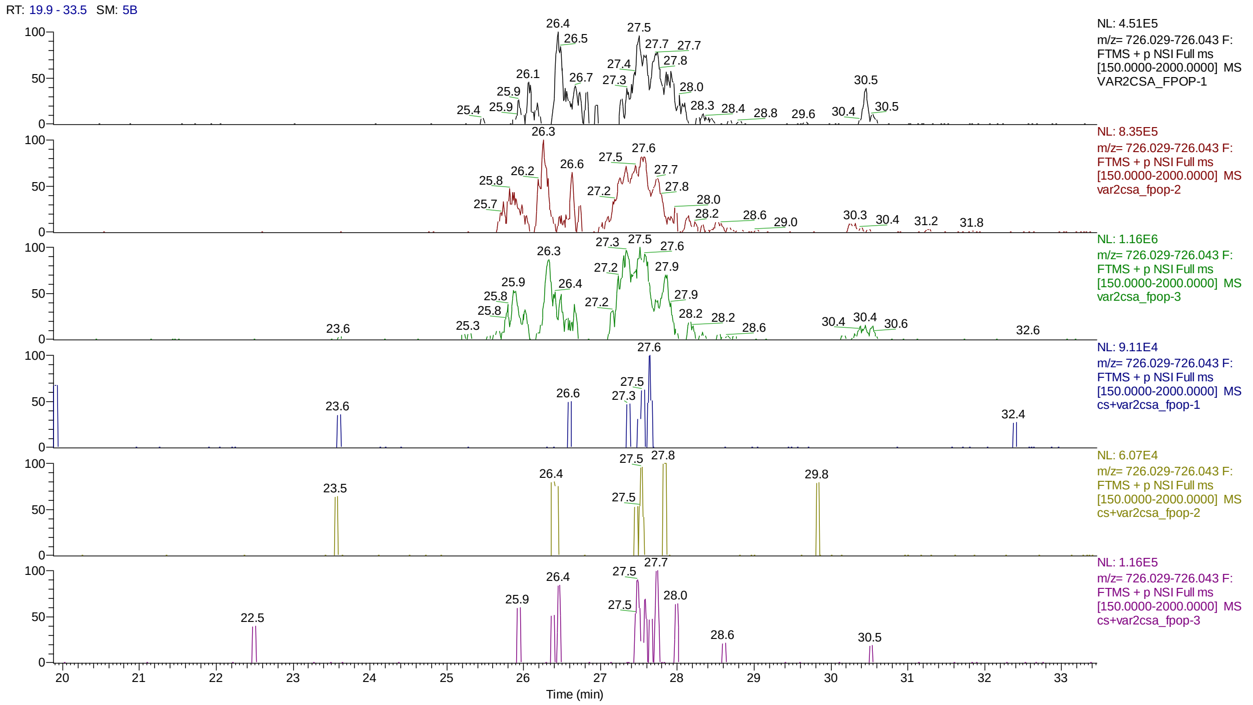

+2 ox

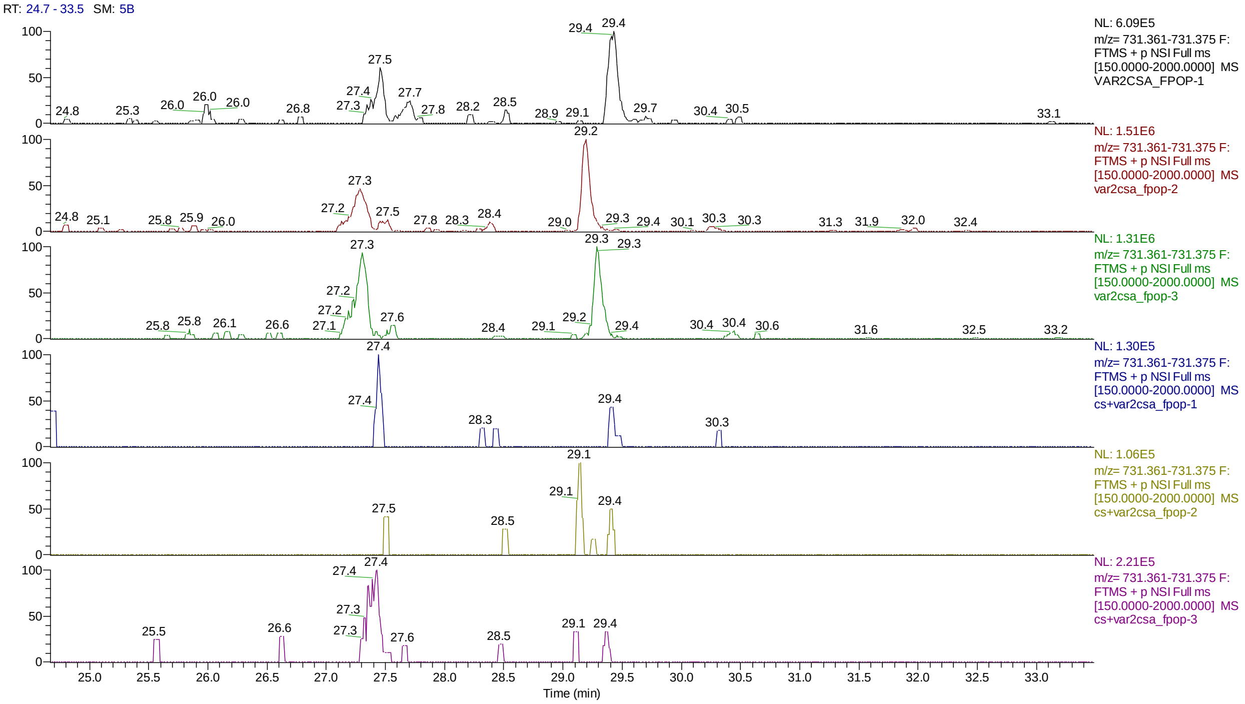
RT: 24.7 - 33.5 SM: 5B
NL: 6.09E5
m/z= 731.361-731.375 F: FTMS + p NSI Full ms [150.0000-2000.0000] MS VAR2CSA_FPOP-1
NL: 1.51E6
m/z= 731.361-731.375 F: FTMS + p NSI Full ms [150.0000-2000.0000] MS var2csa_fpop-2
NL: 1.31E6
m/z= 731.361-731.375 F: FTMS + p NSI Full ms [150.0000-2000.0000] MS var2csa_fpop-3
NL: 1.30E5
m/z= 731.361-731.375 F: FTMS + p NSI Full ms [150.0000-2000.0000] MS cs+var2csa_fpop-1
NL: 1.06E5
m/z= 731.361-731.375 F: FTMS + p NSI Full ms [150.0000-2000.0000] MS cs+var2csa_fpop-2
NL: 2.21E5
m/z= 731.361-731.375 F: FTMS + p NSI Full ms [150.0000-2000.0000] MS cs+var2csa_fpop-3
Time (min)

H. KKRYPQNKNSGNKENL [z=3] Position 629-644: no amino acid level data, multiple oxidation products, all of which are reduced upon pICS binding

Unoxidized peptide

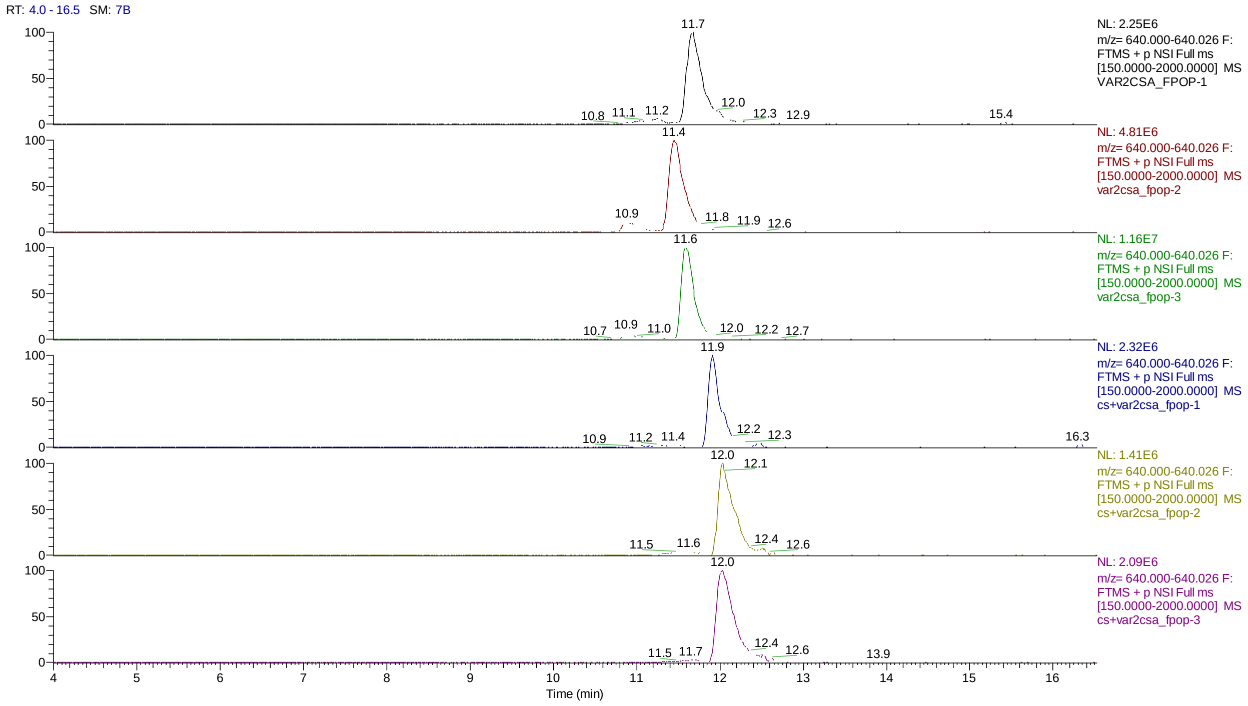

+1 ox

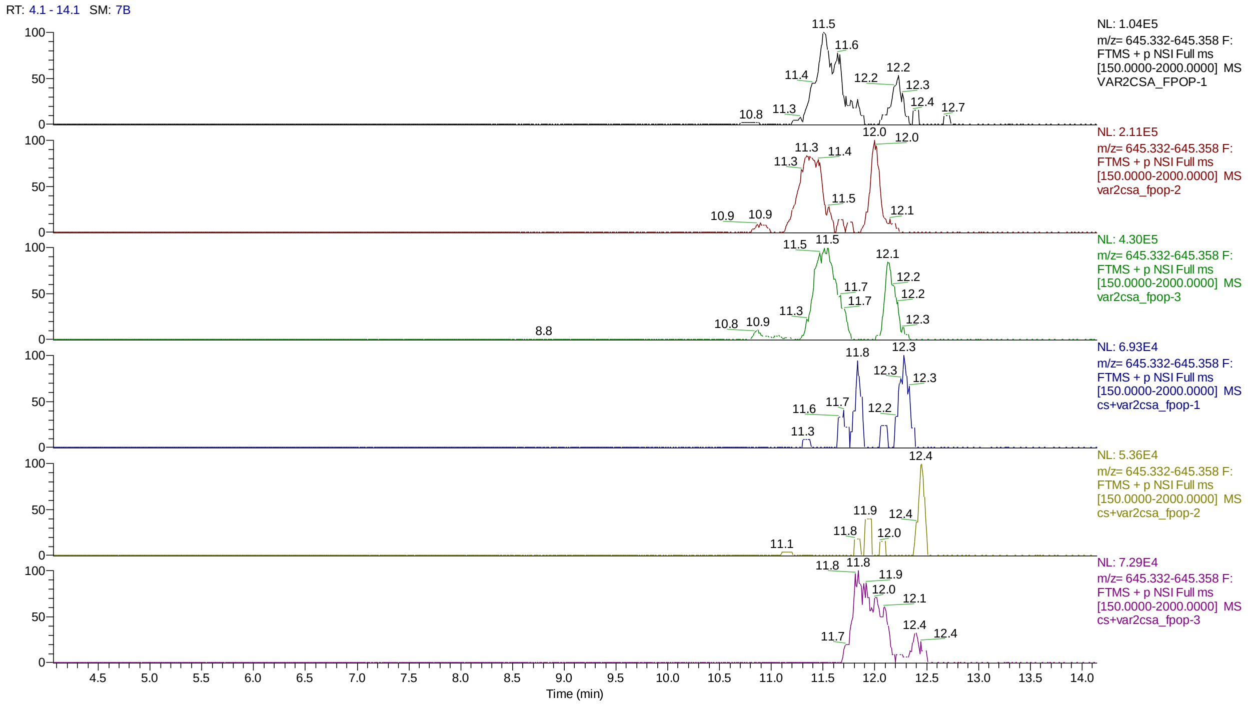

I. **IKKNNTAEQDTSY [z=2] Position 689-701:** Three major oxidation products, all of which are reduced upon pICS binding: K691; S700 and/or Y701; and A695 and/or E696

Unoxidized peptide

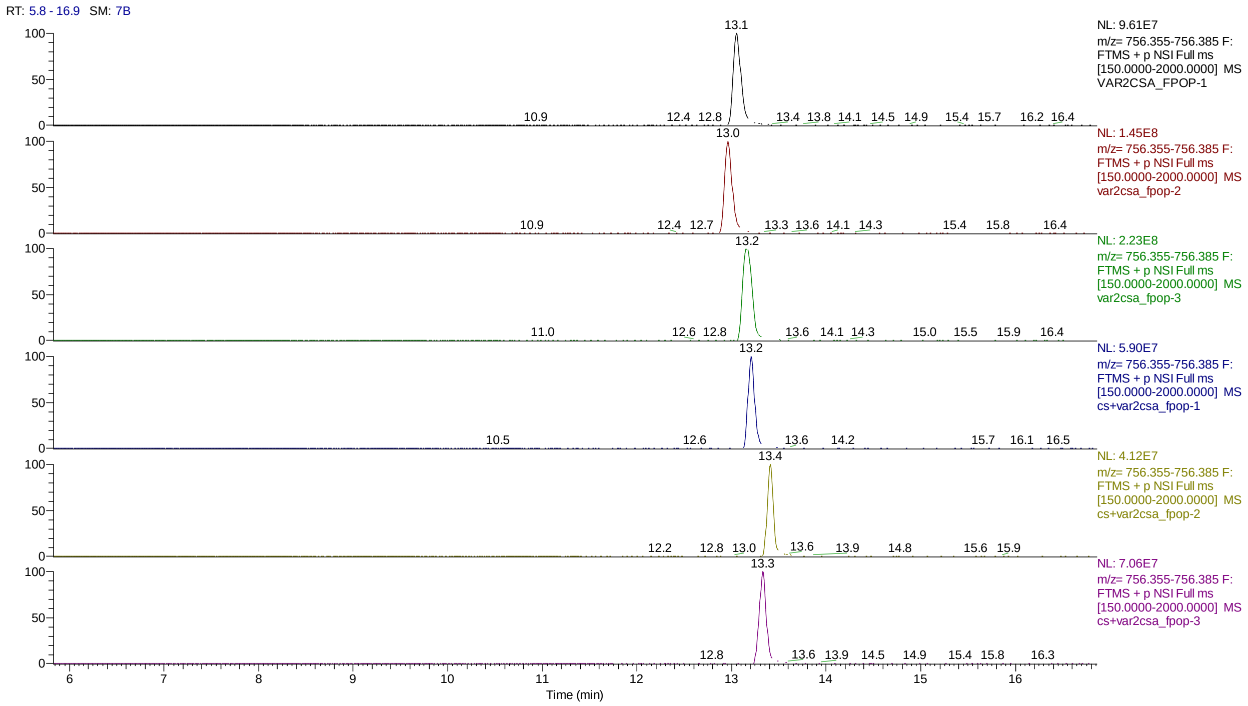

+1 ox

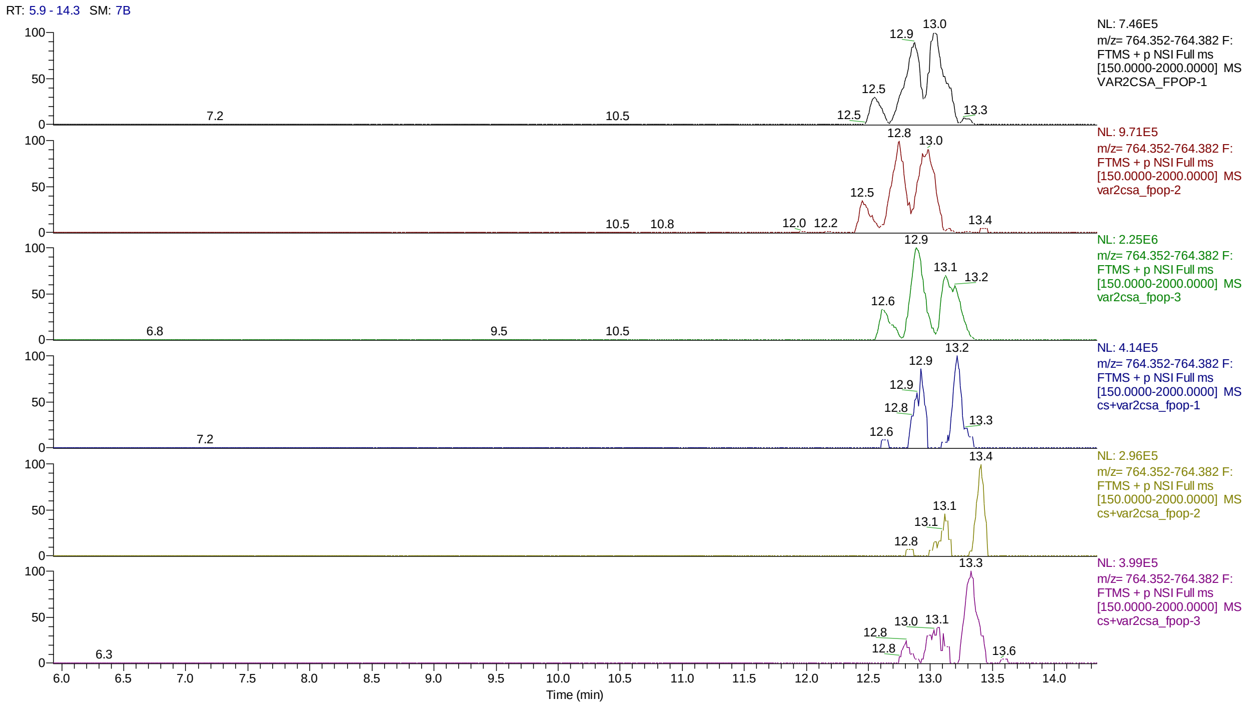

+2 ox

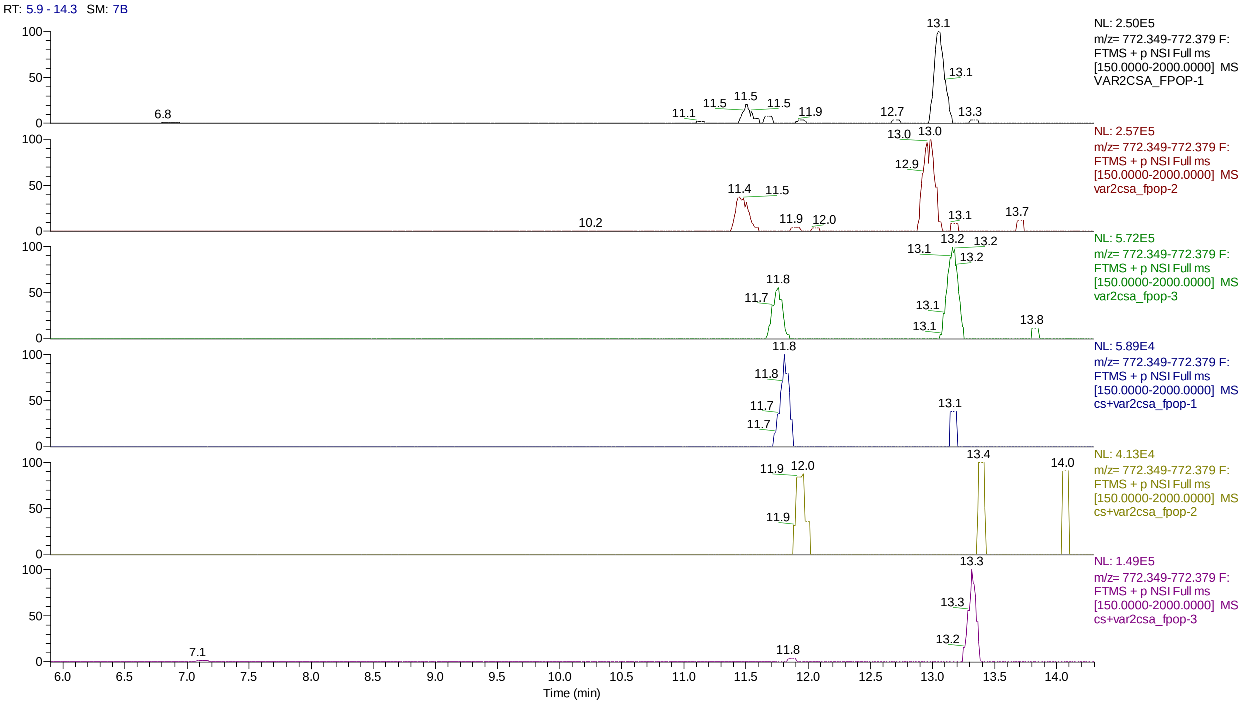

RT: 5.9 - 14.3 SM: 7B
NL: 2.50E5
m/z= 772.349-772.379 F: FTMS + p NSI Full ms [150.0000-2000.0000] MS VAR2CSA_FPOP-1
NL: 2.57E5
m/z= 772.349-772.379 F: FTMS + p NSI Full ms [150.0000-2000.0000] MS var2csa_fpop-2
NL: 5.72E5
m/z= 772.349-772.379 F: FTMS + p NSI Full ms [150.0000-2000.0000] MS var2csa_fpop-3
NL: 5.89E4
m/z= 772.349-772.379 F: FTMS + p NSI Full ms [150.0000-2000.0000] MS cs+var2csa_fpop-1
NL: 4.13E4
m/z= 772.349-772.379 F: FTMS + p NSI Full ms [150.0000-2000.0000] MS cs+var2csa_fpop-2
NL: 1.49E5
m/z= 772.349-772.379 F: FTMS + p NSI Full ms [150.0000-2000.0000] MS cs+var2csa_fpop-3
Time (min)

J. **SSLDELRESW [z=2] Position 702-711:** Major oxidation product identified in oxidation of W711, which is reduced upon pICS binding

Unoxidized peptide

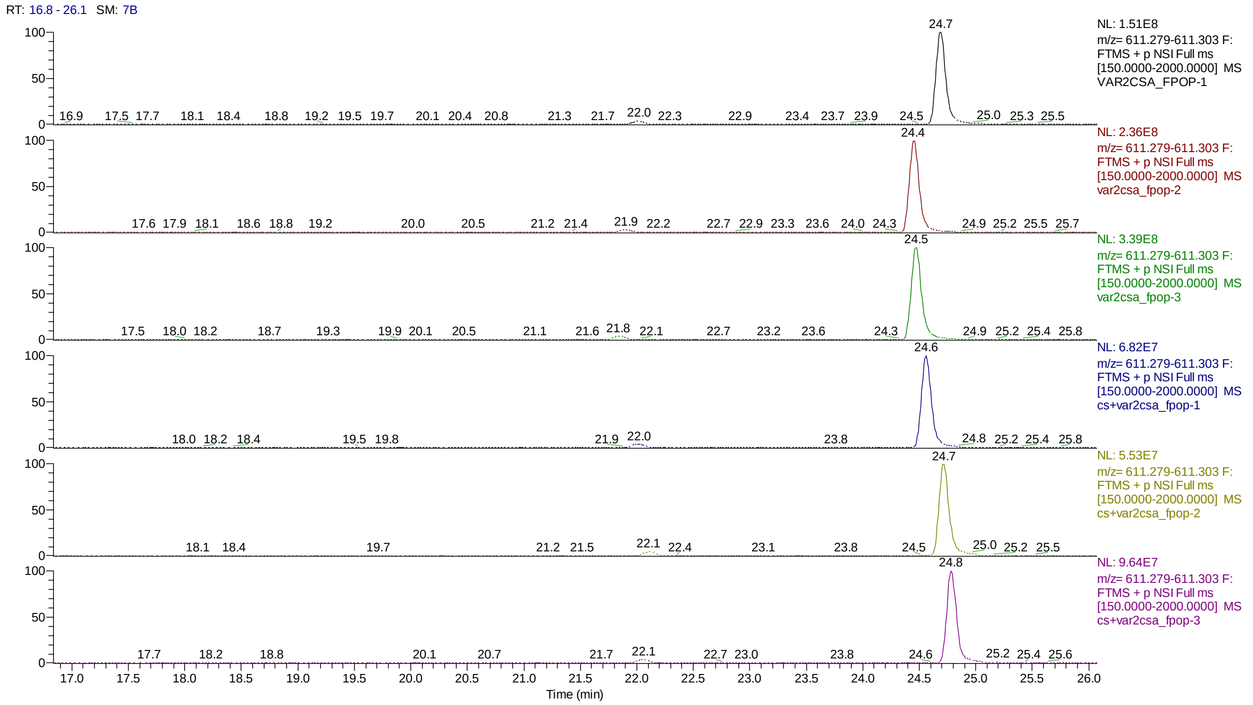

+1 ox

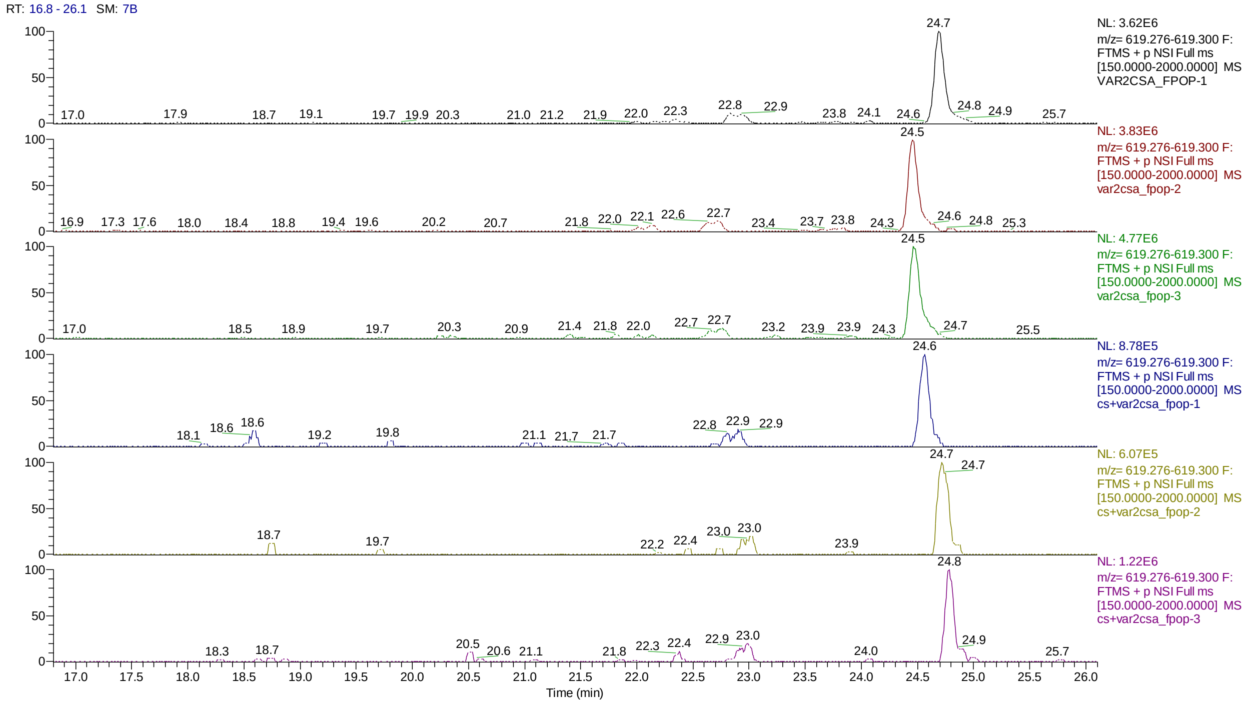

+2 ox

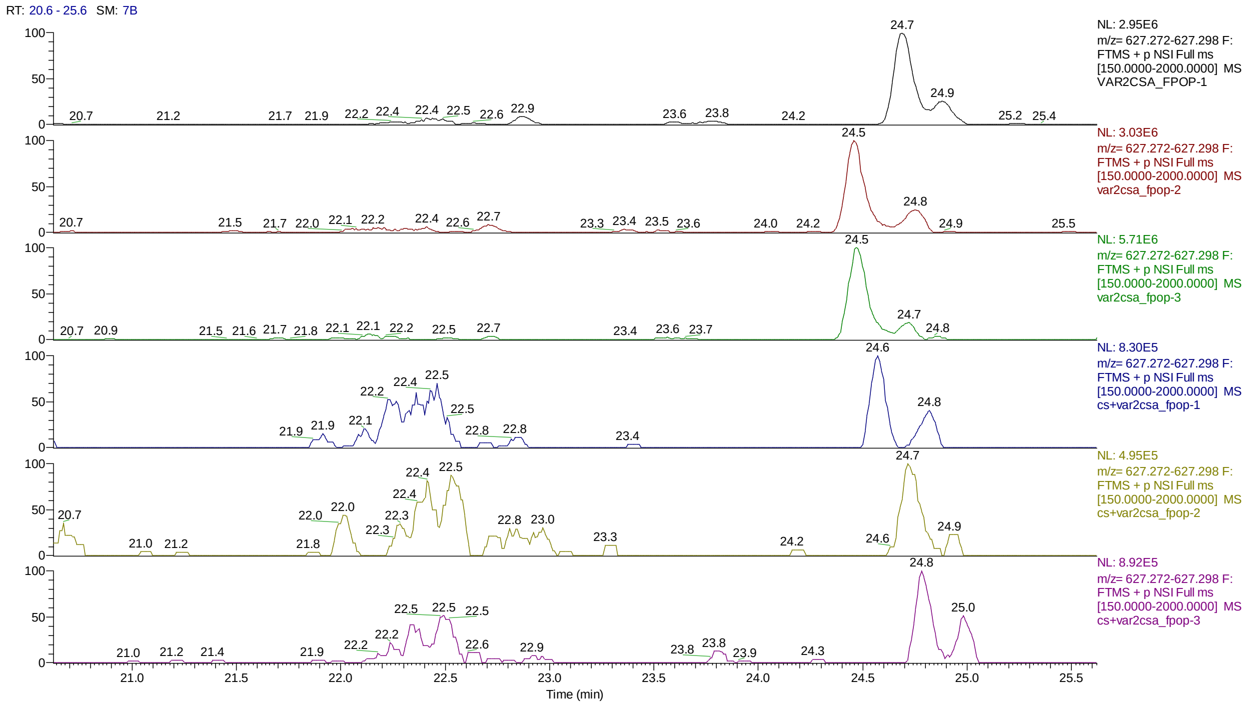
RT: 20.6 - 25.6 SM: 7B
NL: 2.95E6
m/z= 627.272-627.298 F: FTMS + p NSI Full ms [150.0000-2000.0000] MS VAR2CSA_FPOP-1
NL: 3.03E6
m/z= 627.272-627.298 F: FTMS + p NSI Full ms [150.0000-2000.0000] MS var2csa_fpop-2
NL: 5.71E6
m/z= 627.272-627.298 F: FTMS + p NSI Full ms [150.0000-2000.0000] MS var2csa_fpop-3
NL: 8.30E5
m/z= 627.272-627.298 F: FTMS + p NSI Full ms [150.0000-2000.0000] MS cs+var2csa_fpop-1
NL: 4.95E5
m/z= 627.272-627.298 F: FTMS + p NSI Full ms [150.0000-2000.0000] MS cs+var2csa_fpop-2
NL: 8.92E5
m/z= 627.272-627.298 F: FTMS + p NSI Full ms [150.0000-2000.0000] MS cs+var2csa_fpop-3
Time (min)

K. IEACGTAGGGIFTAGSPW [z=2] Position 814-831: W831 is major oxidation product, and is reduced upon pICS binding; other major oxidation products are unidentified, but also reduced upon pICS binding

Unoxidized peptide

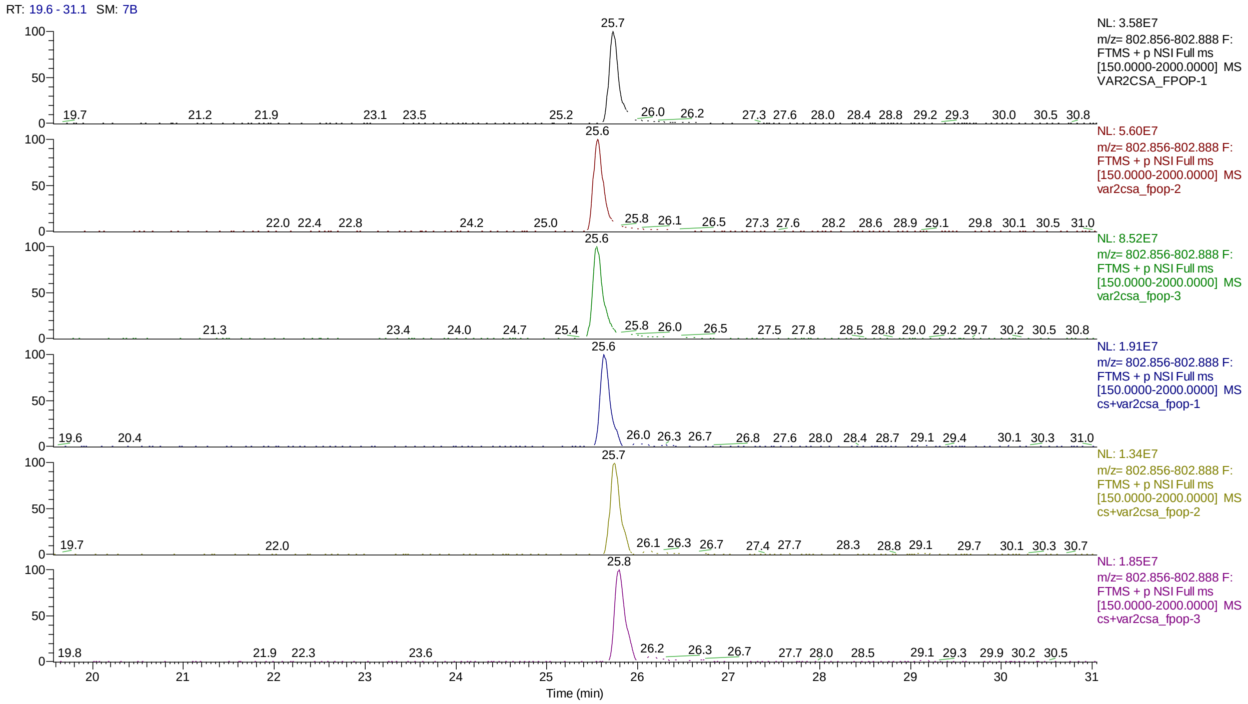

+1 ox

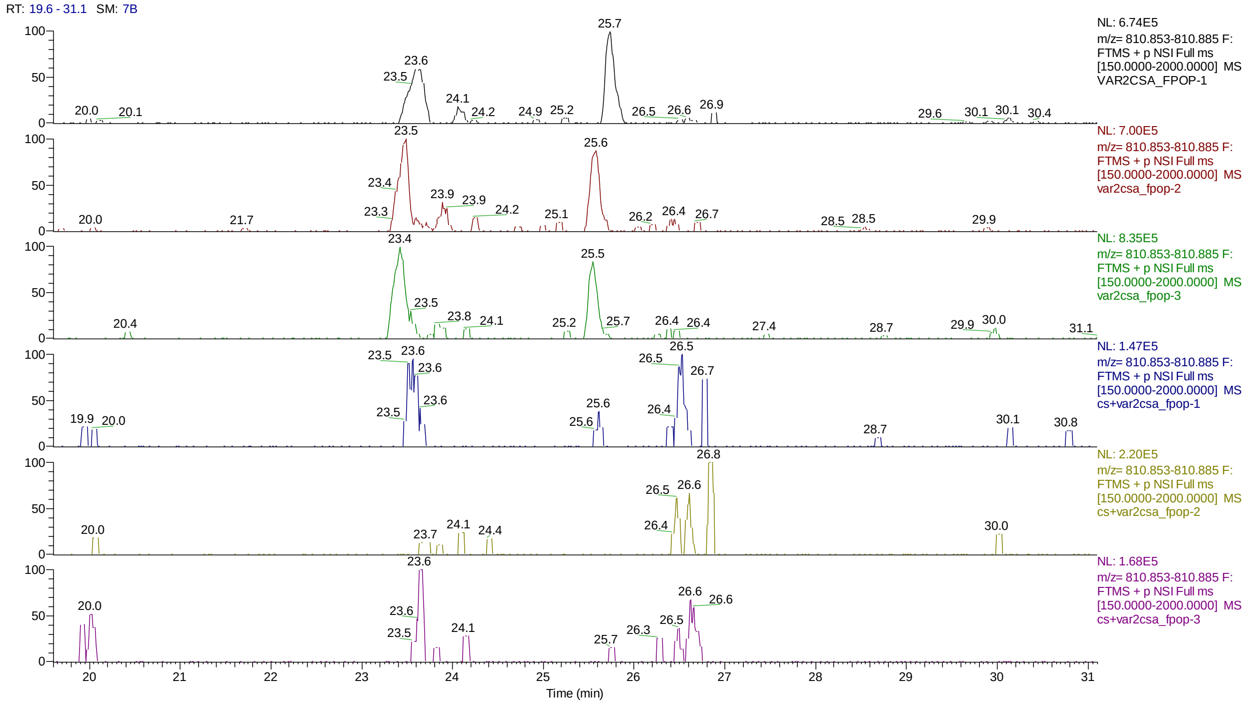

L. LSNVLDDNICGADKAPW [z=2] Position 901-917: No residue-level data; all major oxidation products are reduced upon pICS binding

Unoxidized peptide

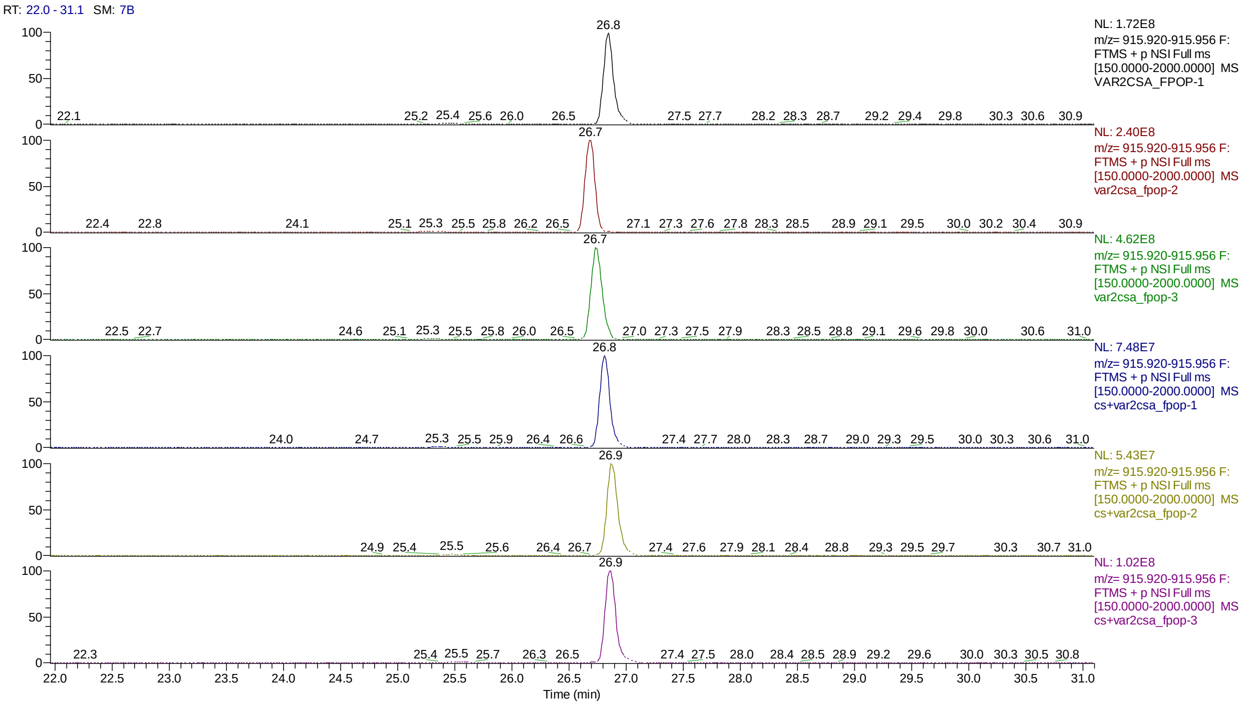

+1 ox

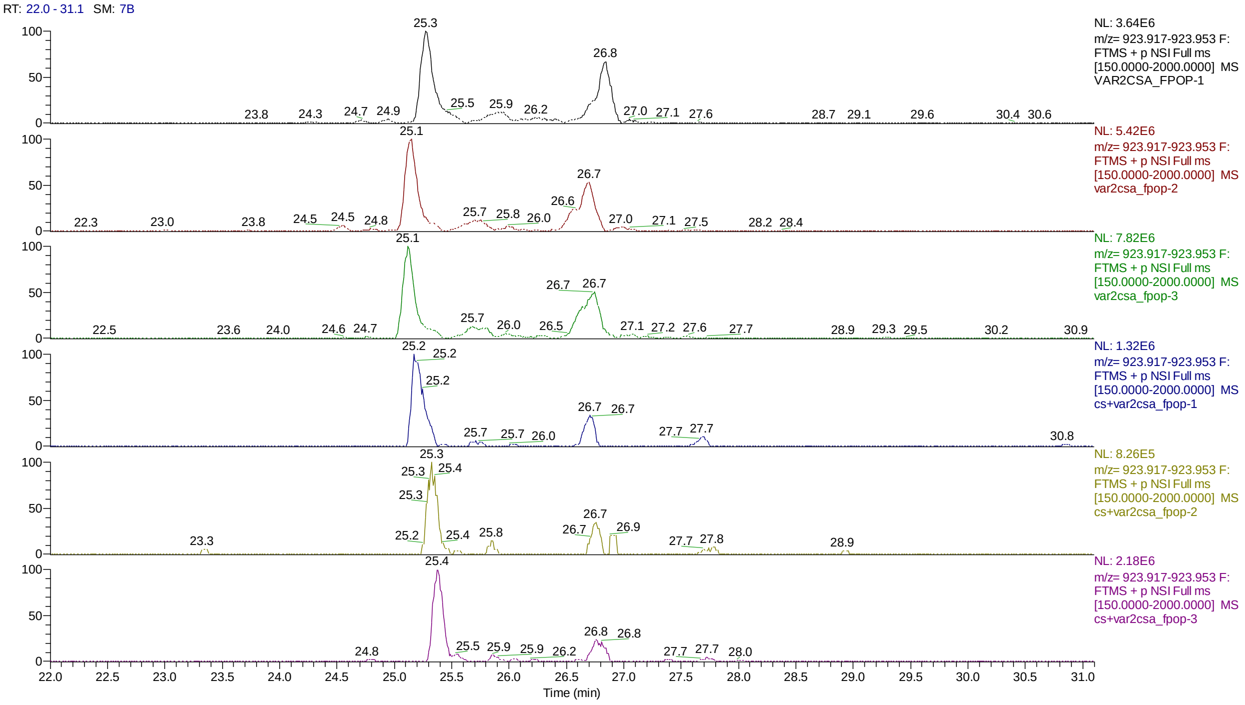

+2 ox

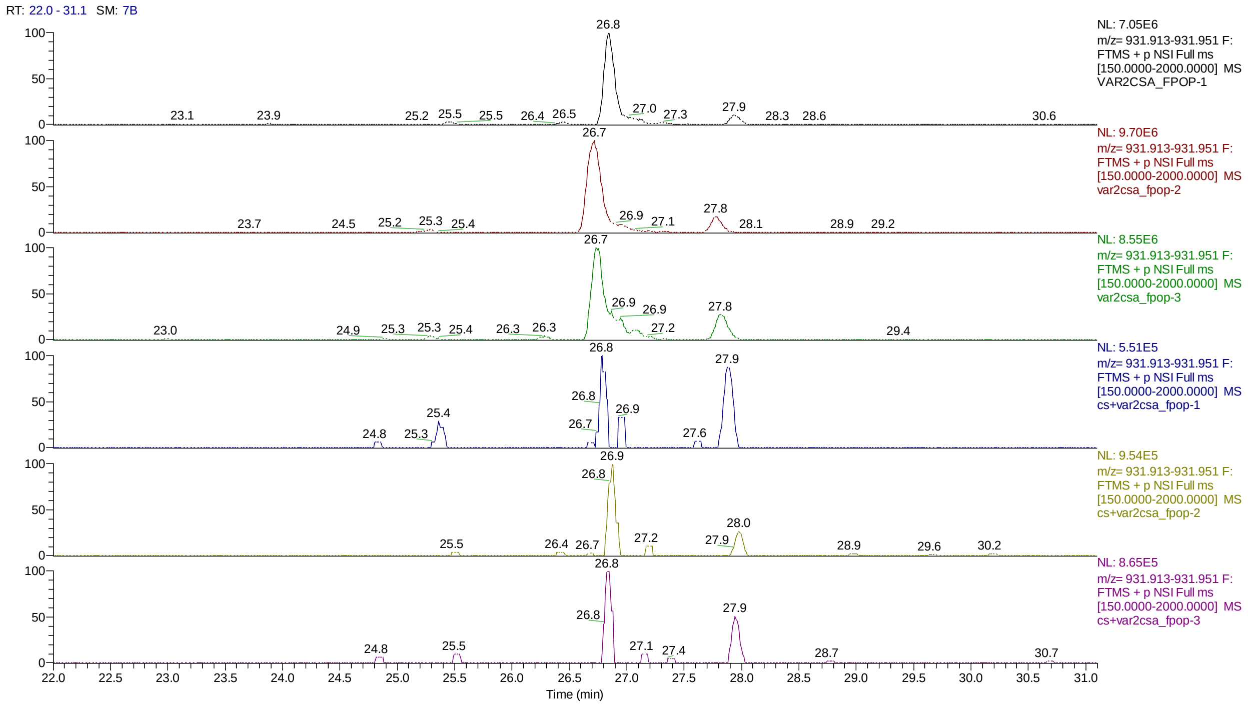

RT: 22.0 - 31.1 SM: 7B
NL: 7.05E6
m/z= 931.913-931.951 F: FTMS + p NSI Full ms [150.0000-2000.0000] MS VAR2CSA_FPOP-1
NL: 9.70E6
m/z= 931.913-931.951 F: FTMS + p NSI Full ms [150.0000-2000.0000] MS var2csa_fpop-2
NL: 8.55E6
m/z= 931.913-931.951 F: FTMS + p NSI Full ms [150.0000-2000.0000] MS var2csa_fpop-3
NL: 5.51E5
m/z= 931.913-931.951 F: FTMS + p NSI Full ms [150.0000-2000.0000] MS cs+var2csa_fpop-1
NL: 9.54E5
m/z= 931.913-931.951 F: FTMS + p NSI Full ms [150.0000-2000.0000] MS cs+var2csa_fpop-2
NL: 8.65E5
m/z= 931.913-931.951 F: FTMS + p NSI Full ms [150.0000-2000.0000] MS cs+var2csa_fpop-3
Time (min)

M. TTTEKCNKERDKSKSQSSDTL [z=3] Position 924-944: No residue-level data; the one major oxidation product is reduced upon pICS binding

Unoxidized peptide

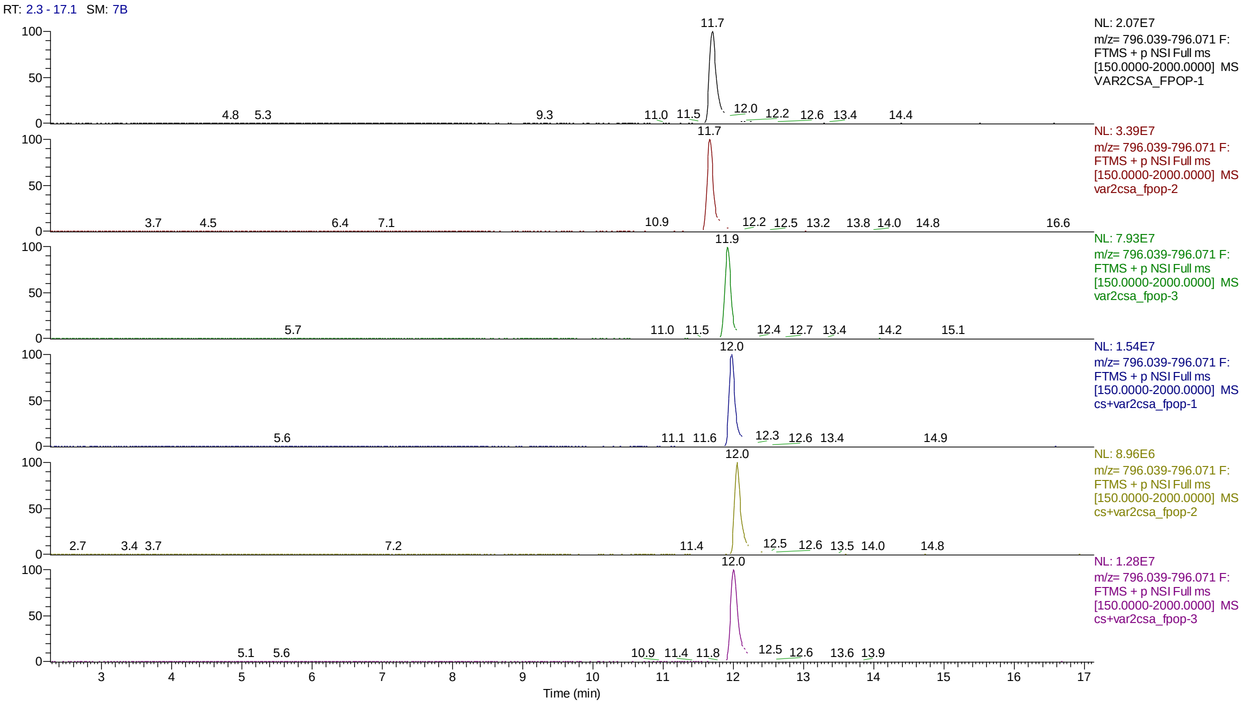

+1 ox

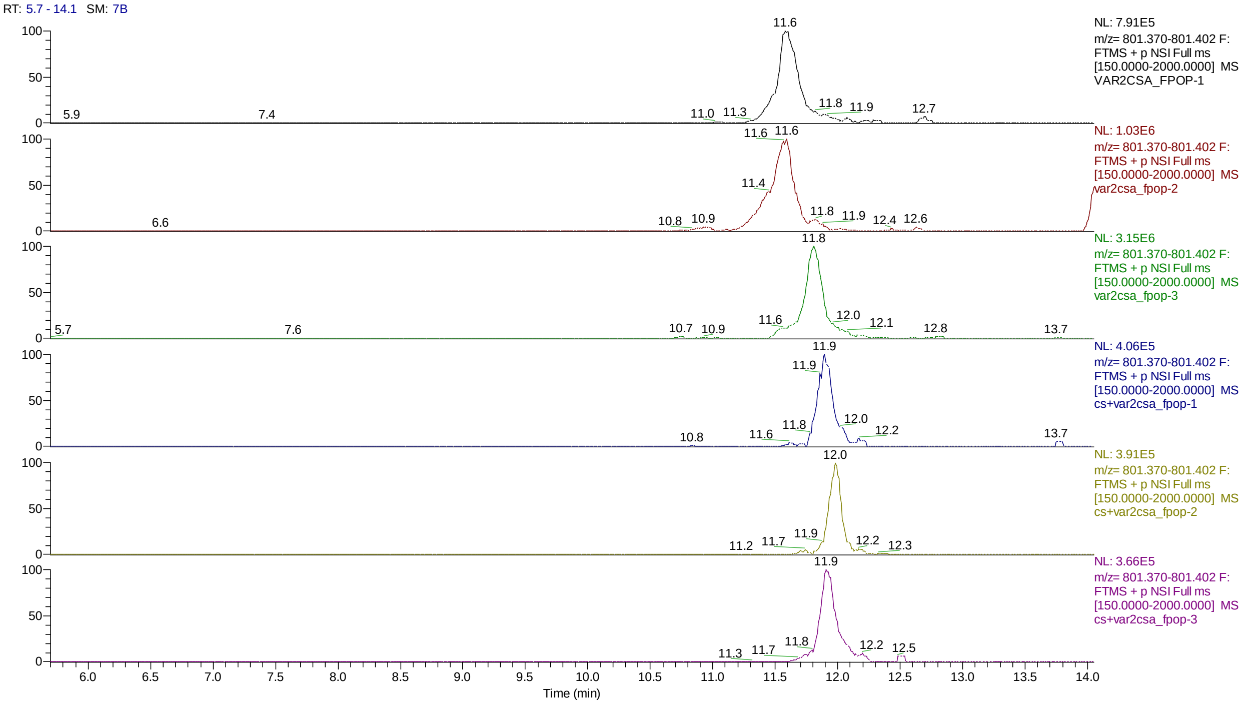

Exposed upon CS binding:

N. EVITCGARSNDLL [z=2] Position 224-236: No residue-level data; one major oxidation product that is significantly increased upon pICS binding. Oxidation of C unlikely due to lack of +2O and +3O oxidation products

Unoxidized peptide

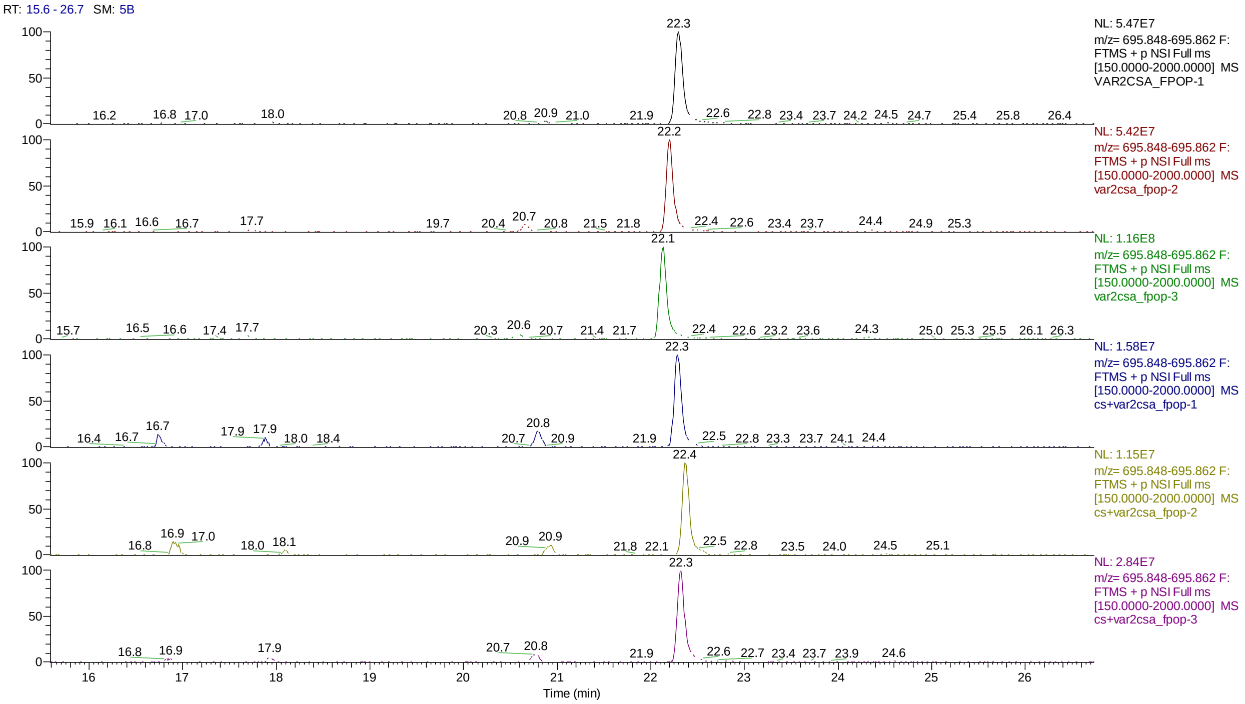

+1 ox

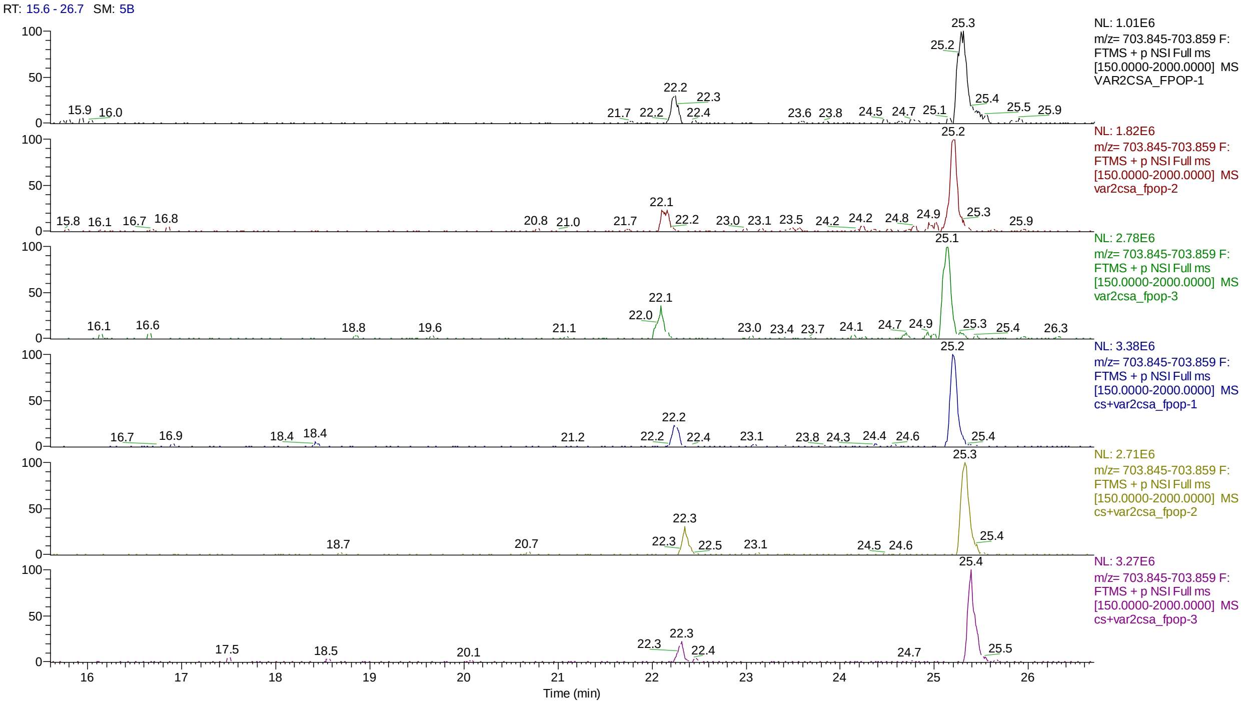

O. DNEYTKDLEL [z=2] 666-675: No amino acid level data; the major oxidation product eluting at ~20.5 min is increased upon pICS binding

Unoxidized peptide

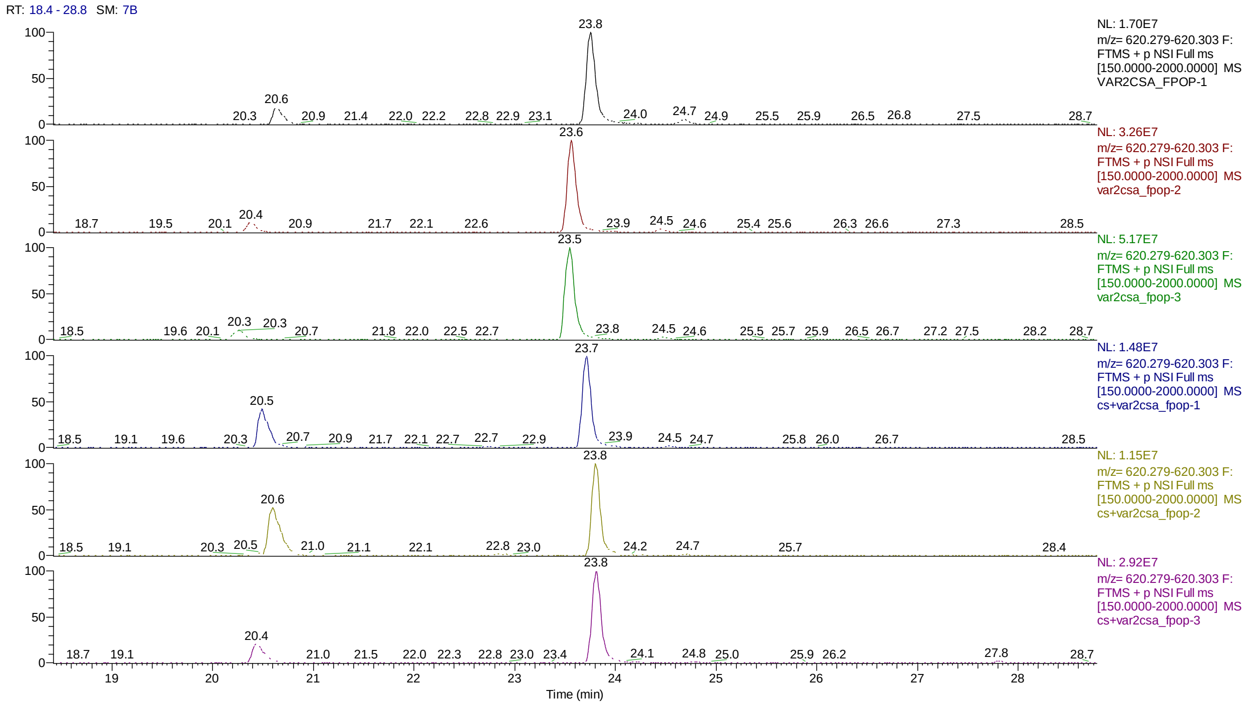

+1 ox

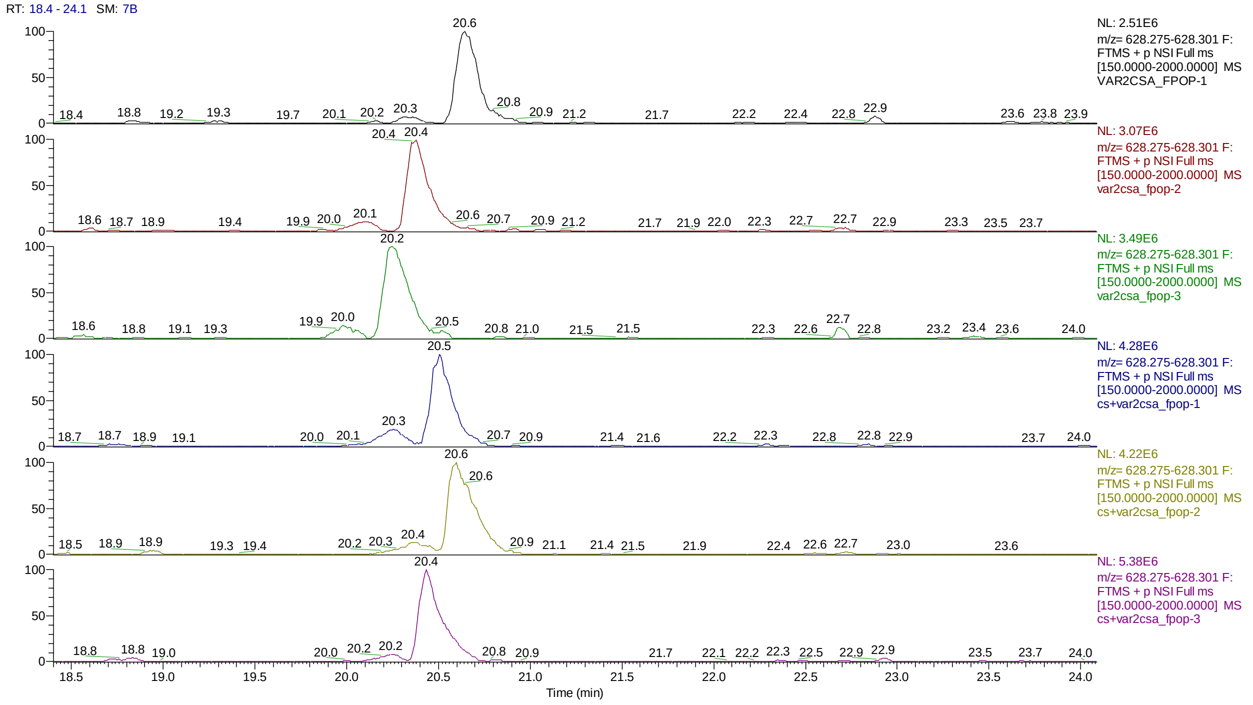

Extracted Ion Chromatograms of the Peptides NOT significantly Changed (p<0.05) of VAR2CSA in the presence of pICS.

P. KGTNSNLEKNLKQMF [z=3] Position 173-187

Unoxidized peptide

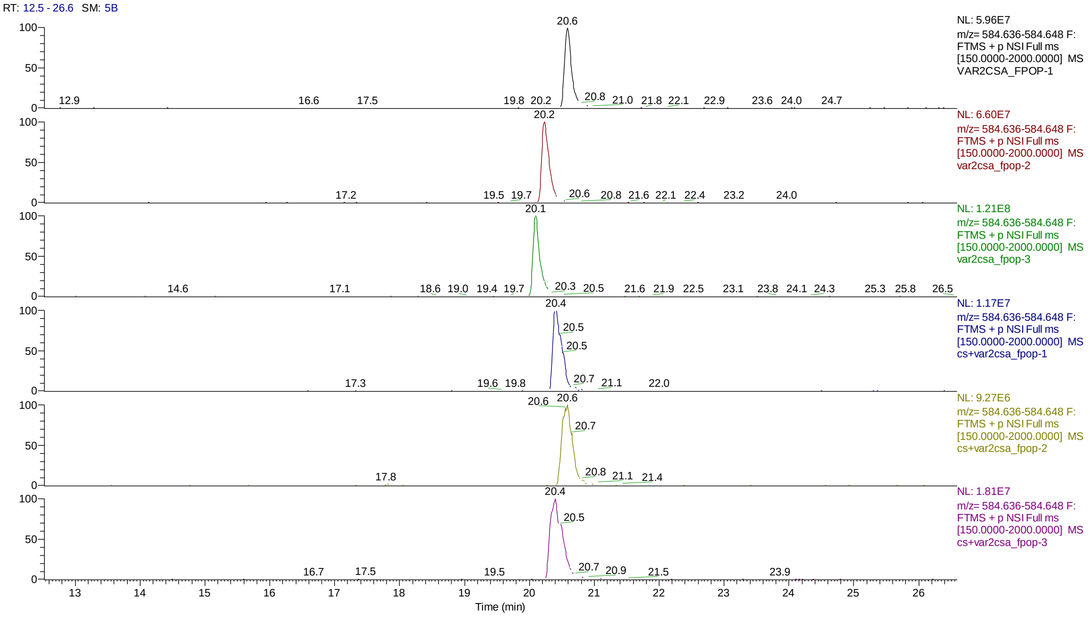

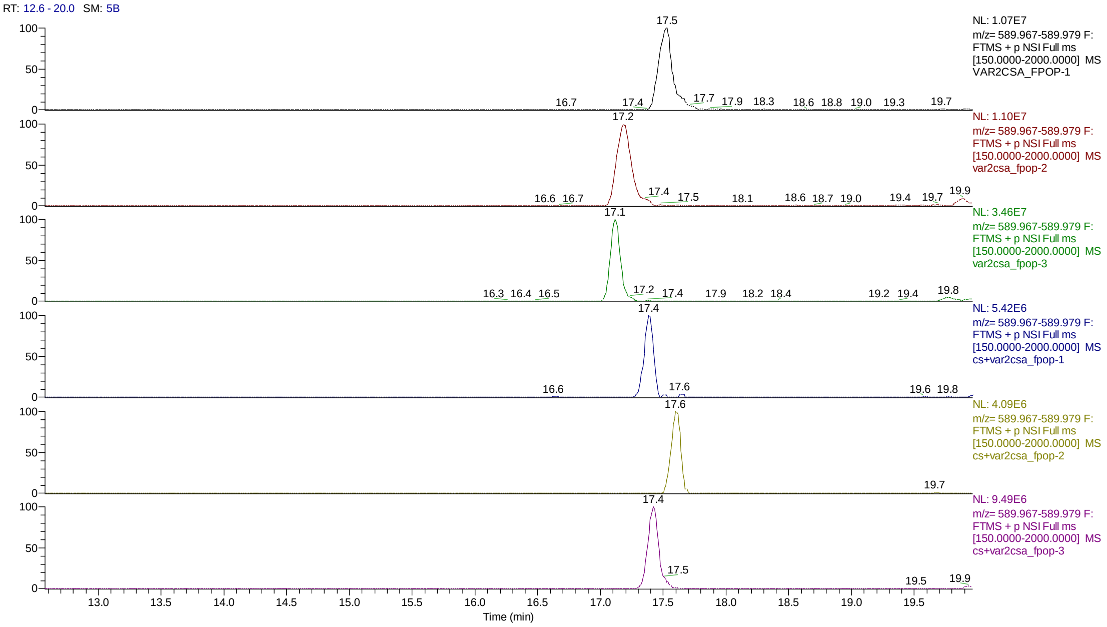

Q. AKIRENDKVLQDKYPKDQKY [z=3] Position 188-207

Unoxidized peptide

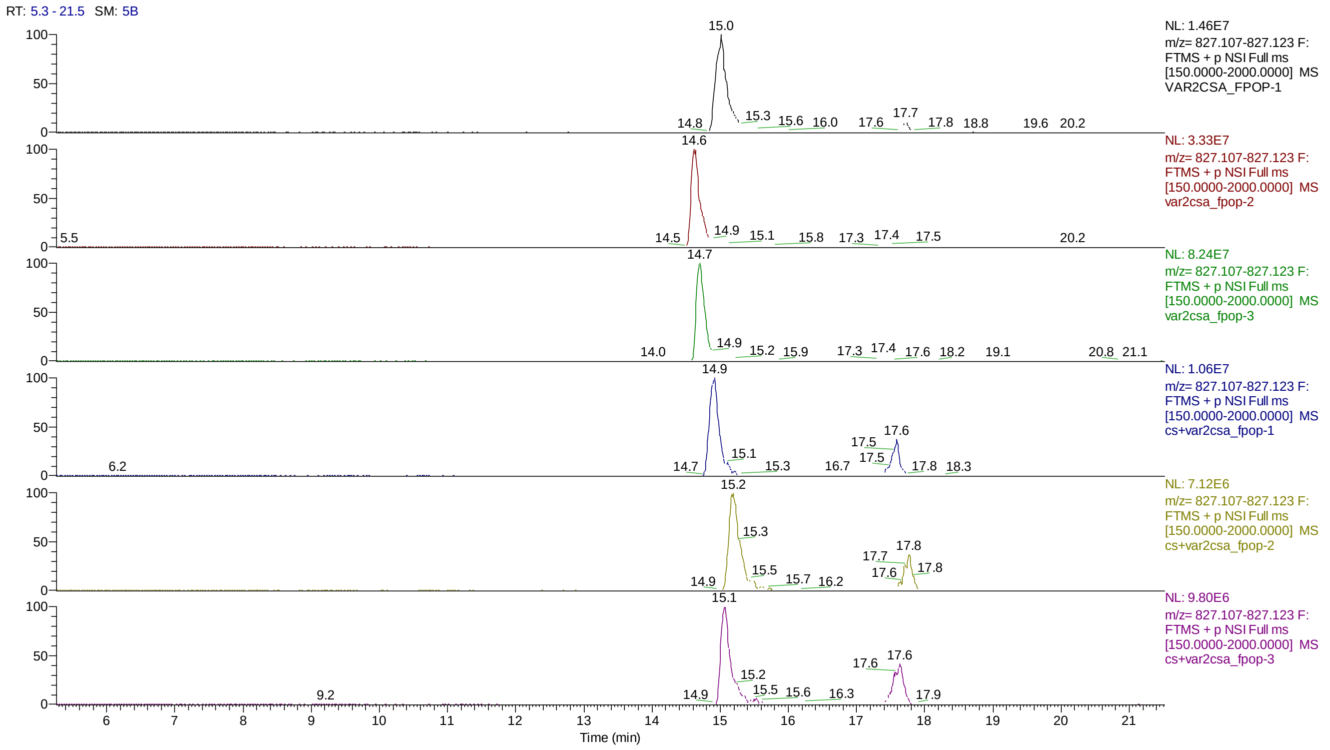

+1 ox

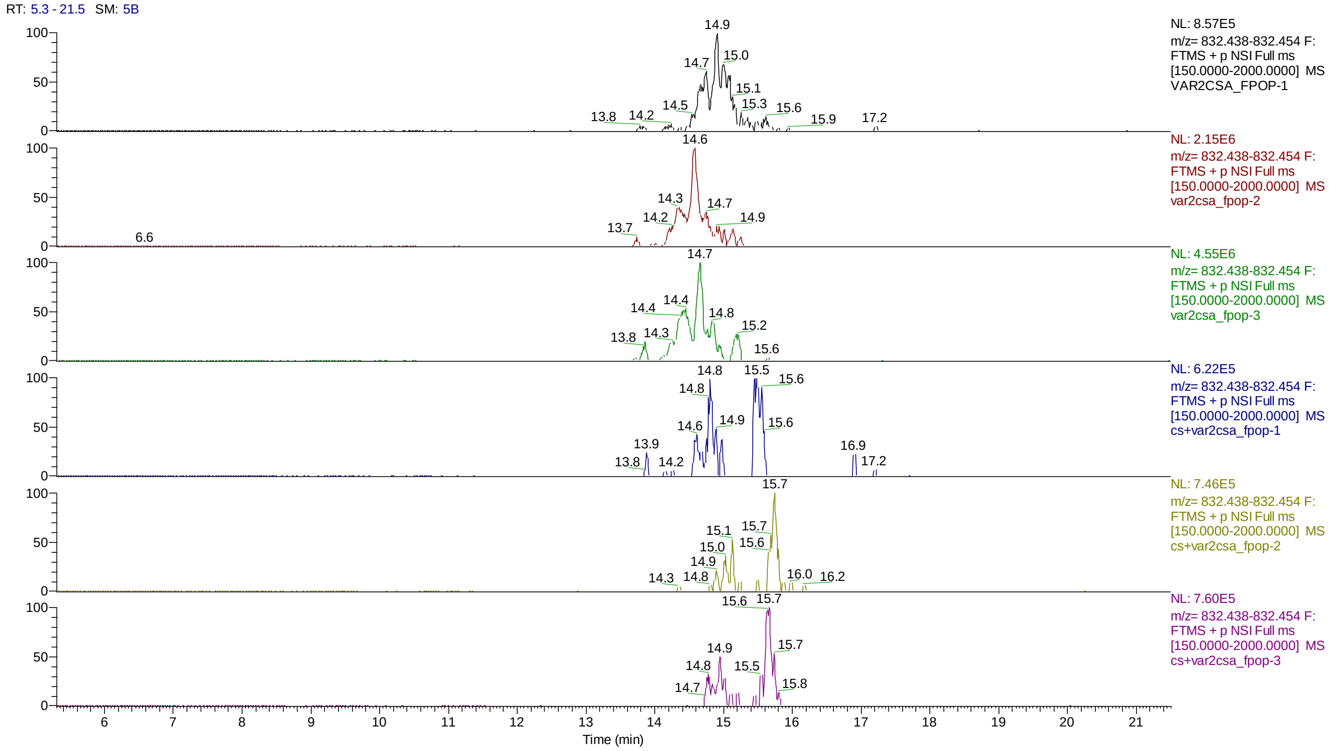

R. TKLREAW [z=2] Position 208-214

Unoxidized peptide

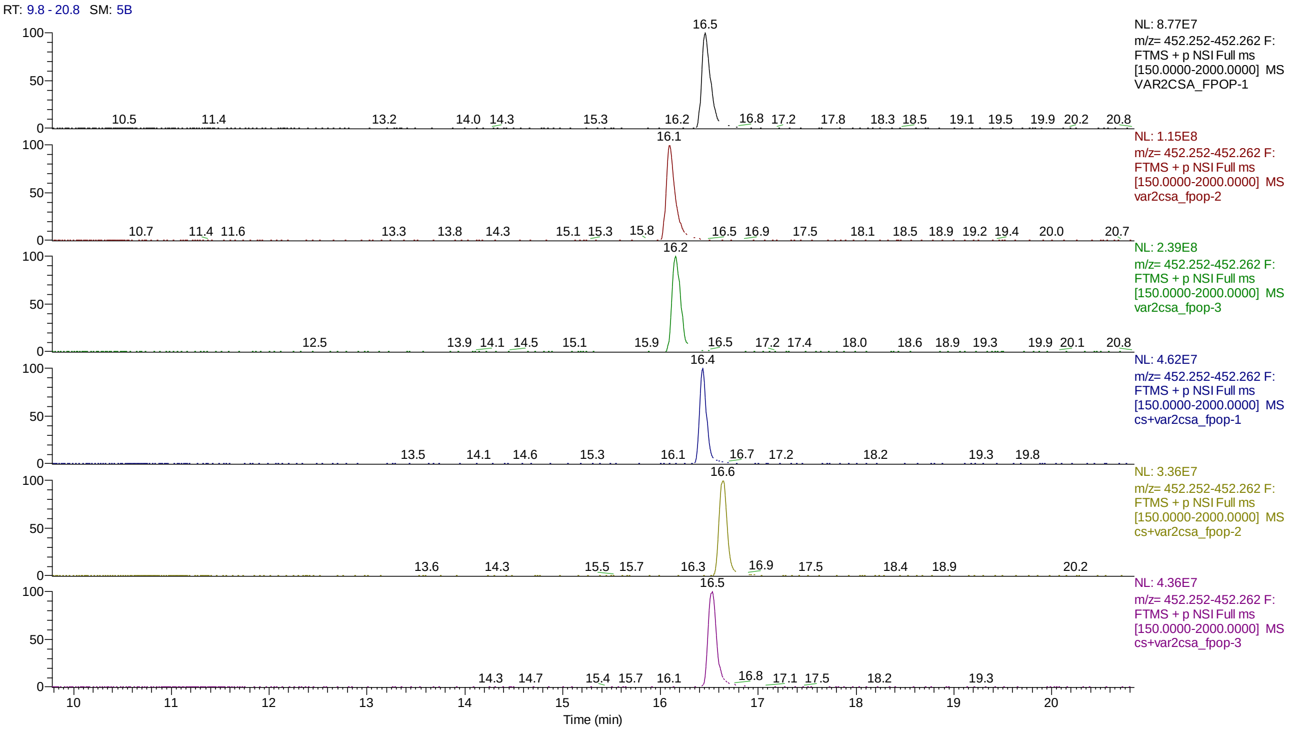

+1 ox

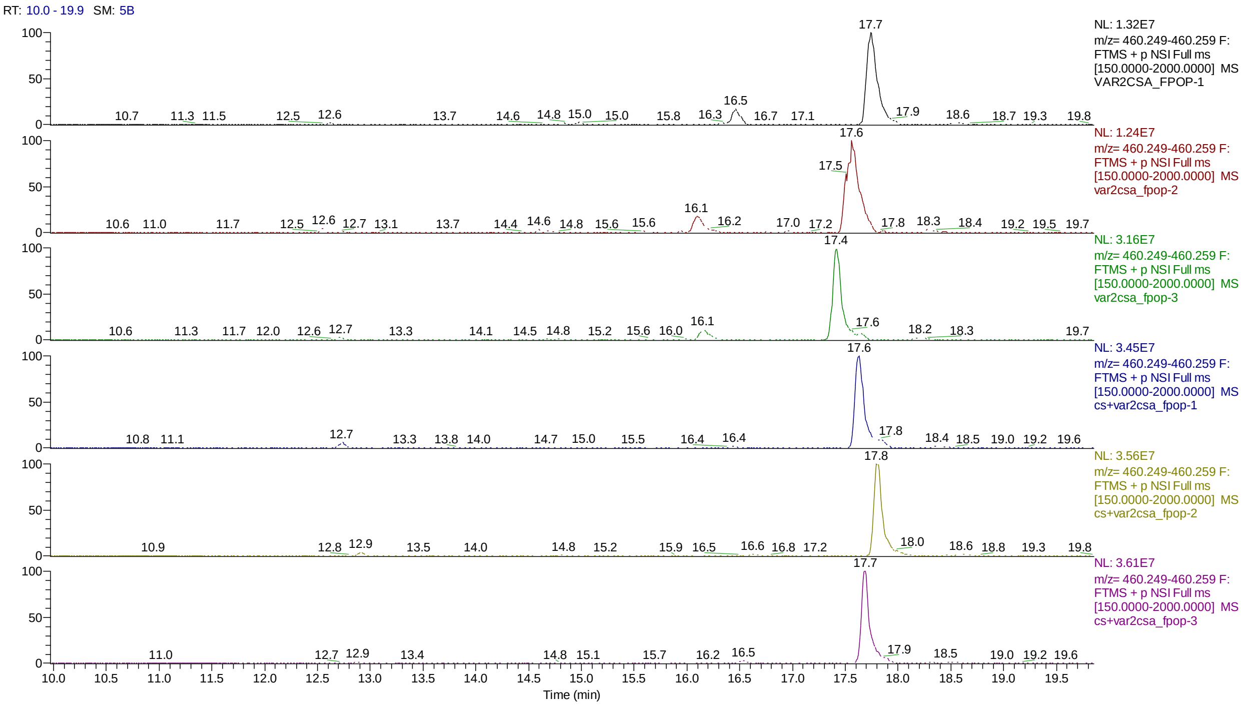

S. NANRQKVW [z=2] Position 216-223

Unoxidized peptide

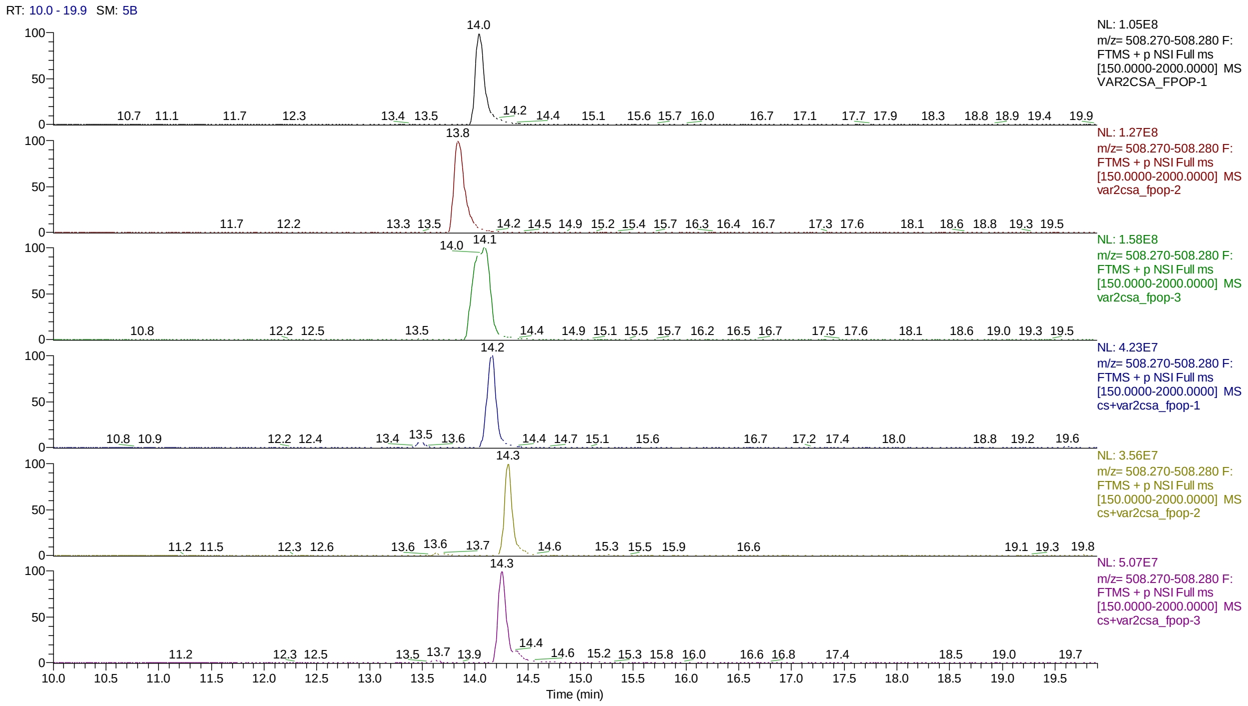

+1 ox

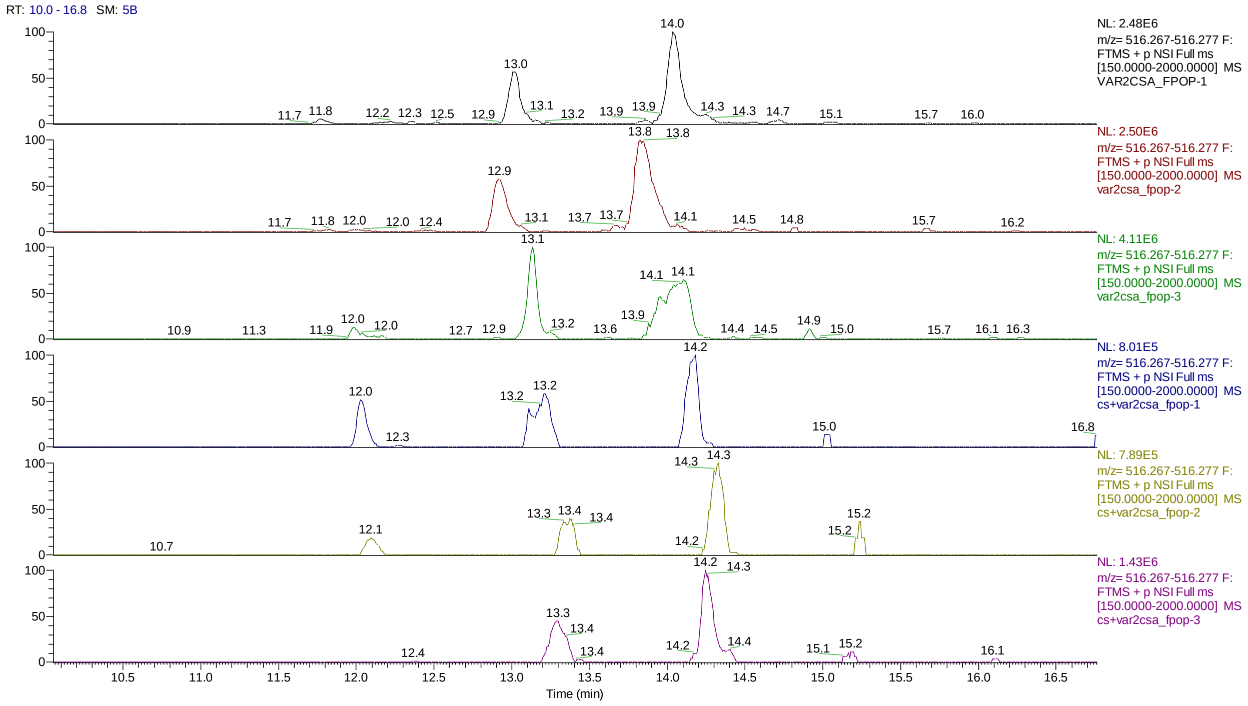

T. LIKRGW [z=2] Position 236-241

Unoxidized peptide

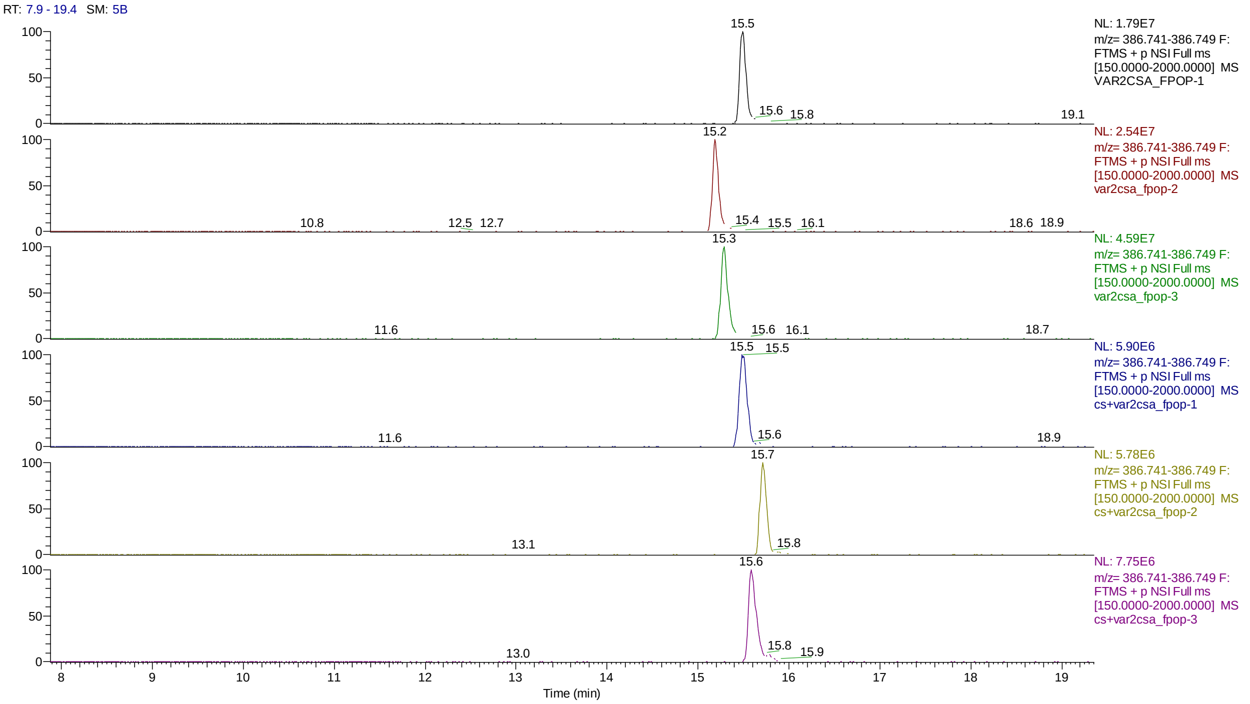

+1 ox

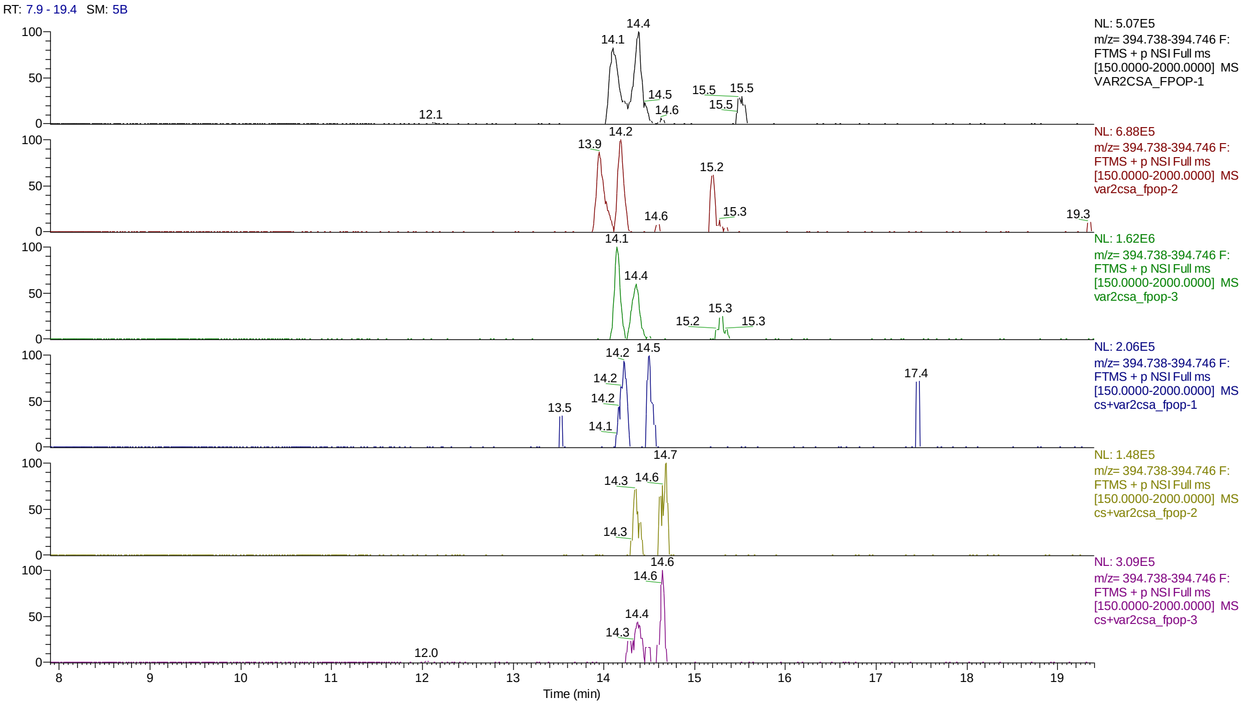

U. DYVPQFL [z=2] Position 271-277

Unoxidized peptide

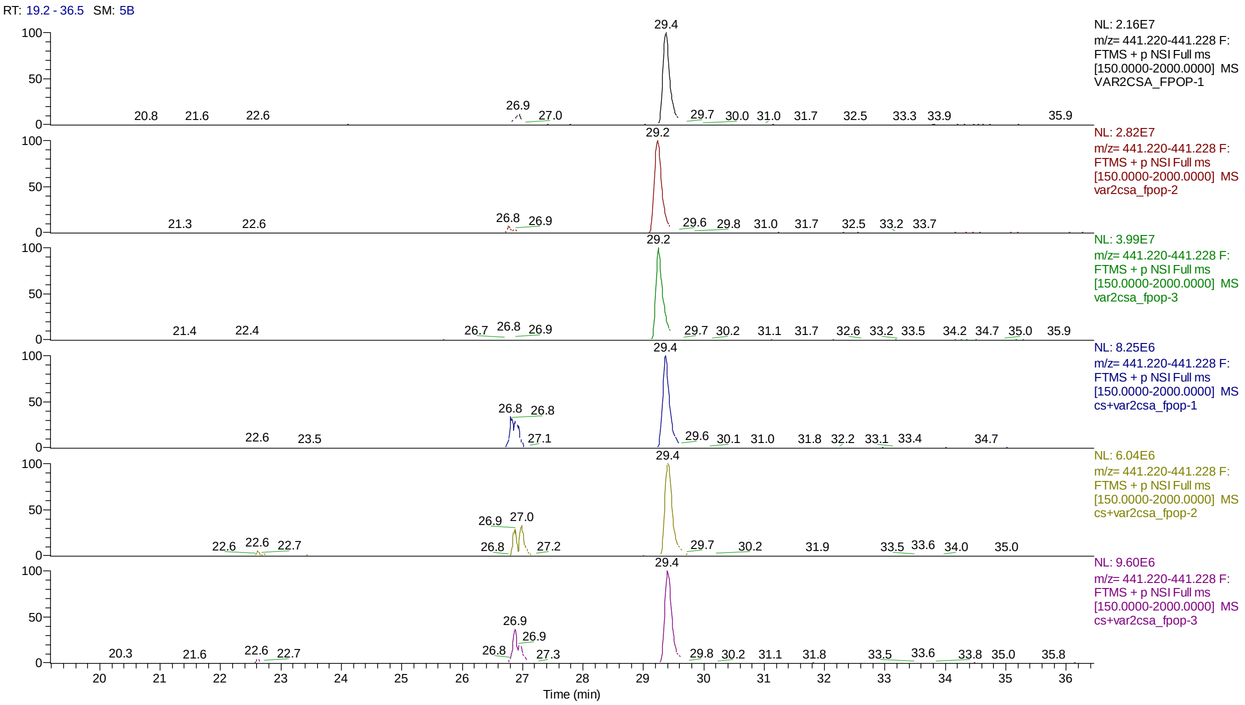

+1 ox

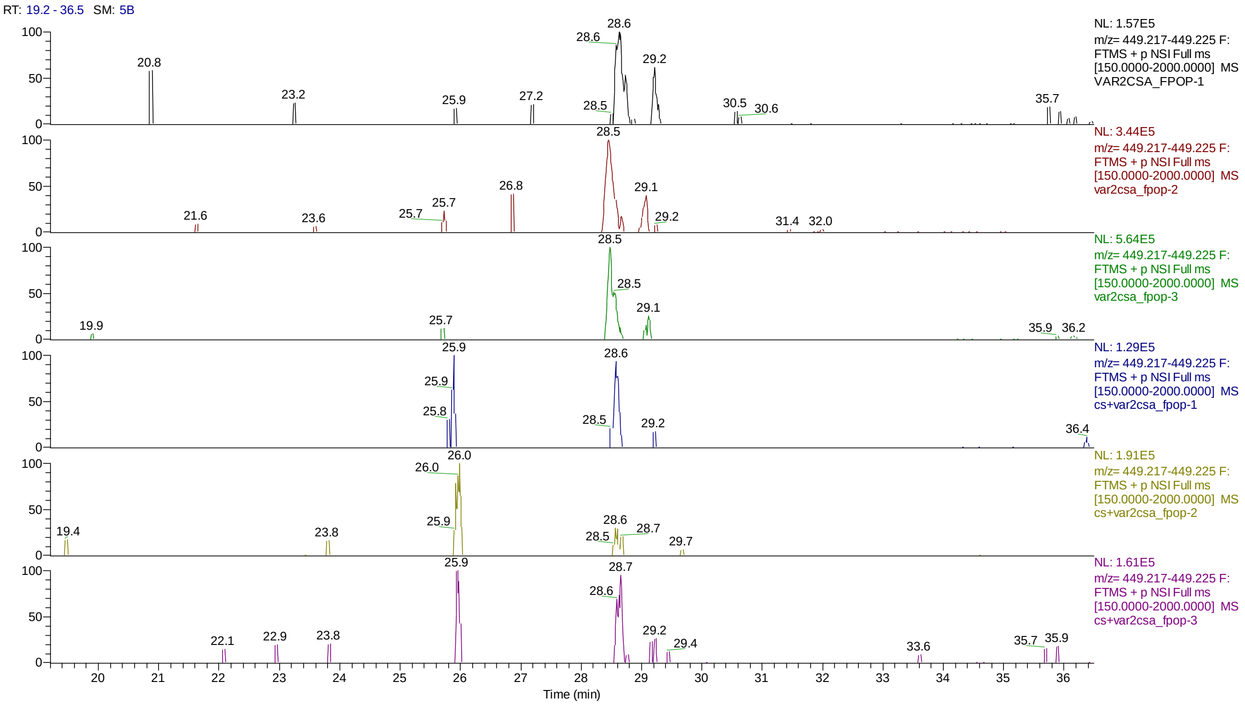

V. KTEWENQENKY [z=3] Position 337-347

Unoxidized peptide

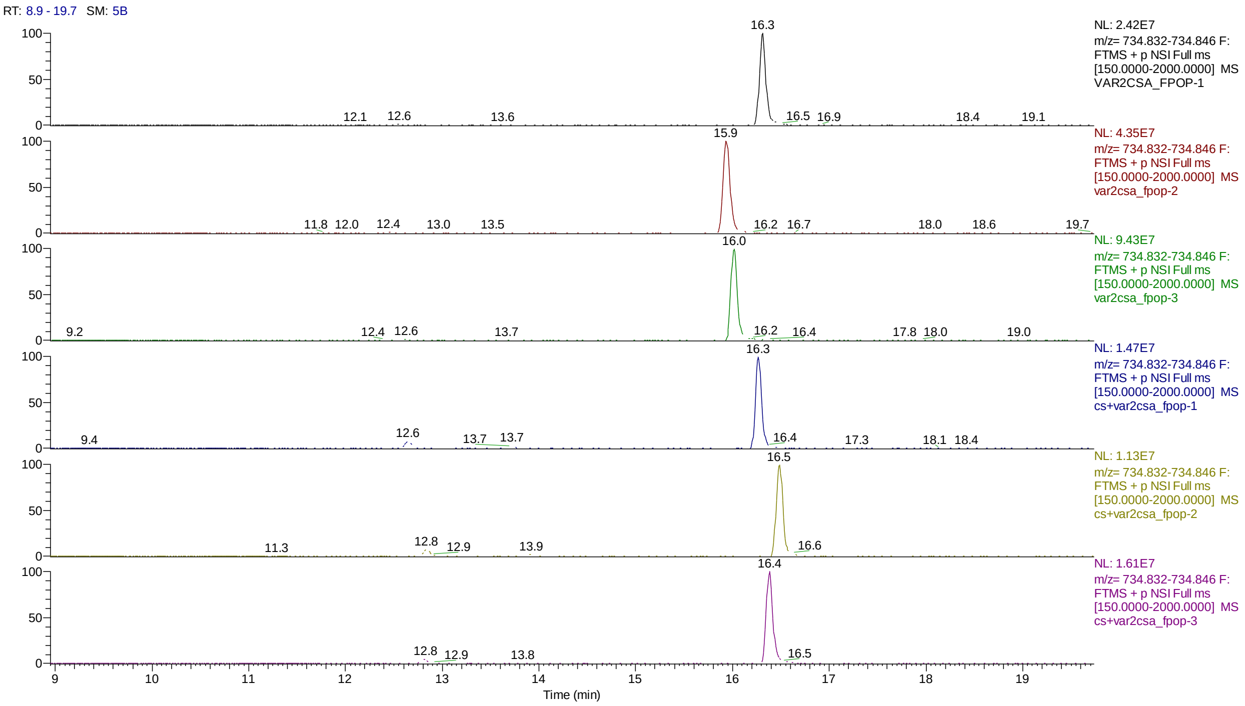

+1 ox

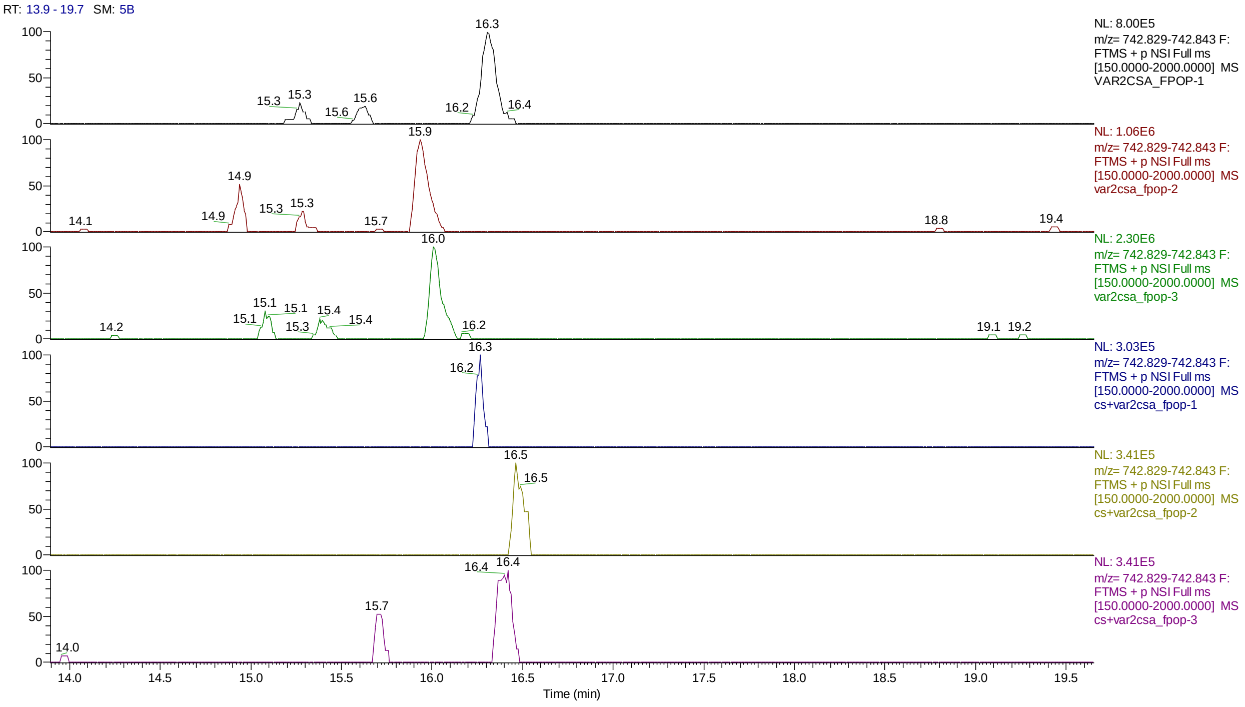

W. GVRENDKDL [z=2] Position 469-477

Unoxidized peptide

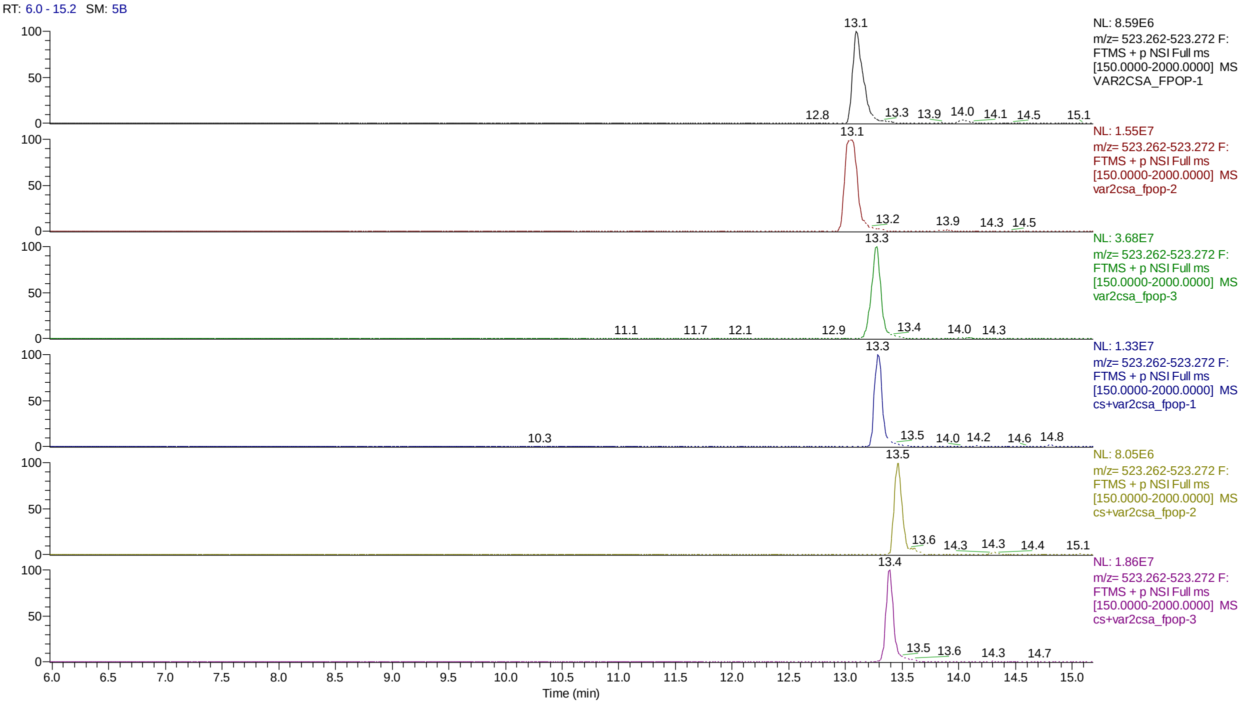

+1 ox

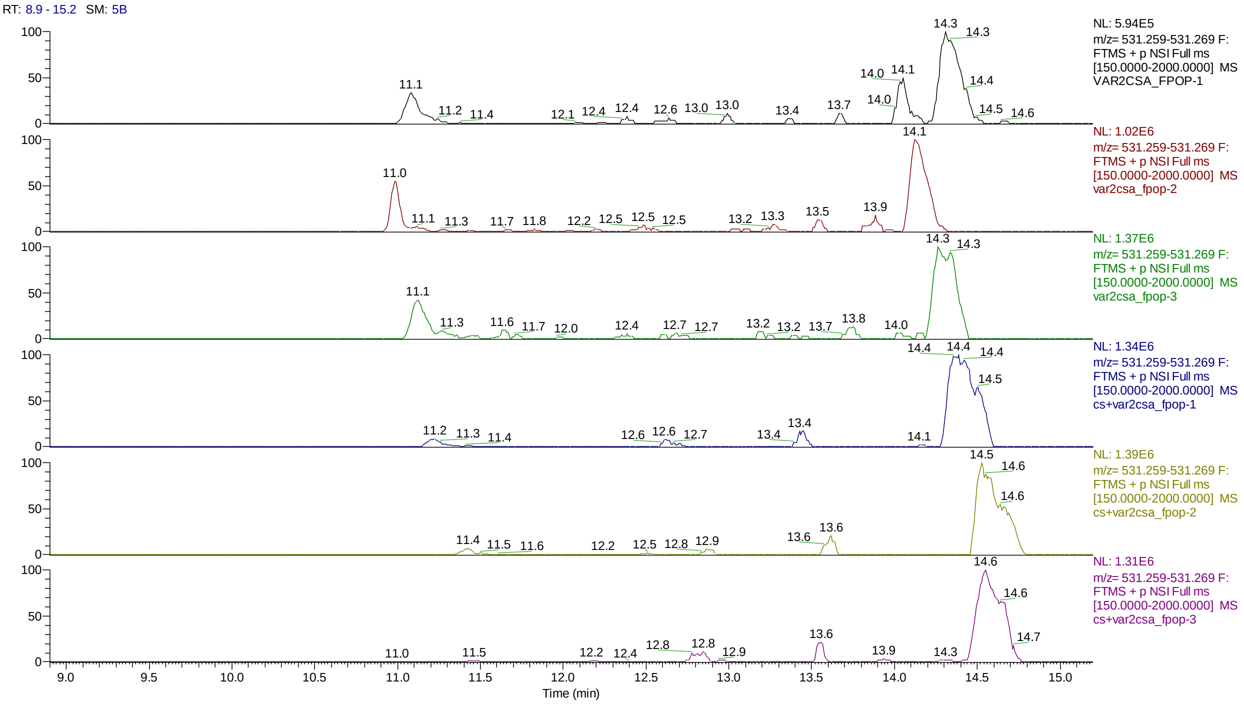

X. DPCEKKLPPYDDNDQW [z=3] Position 65-80

Unoxidized peptide

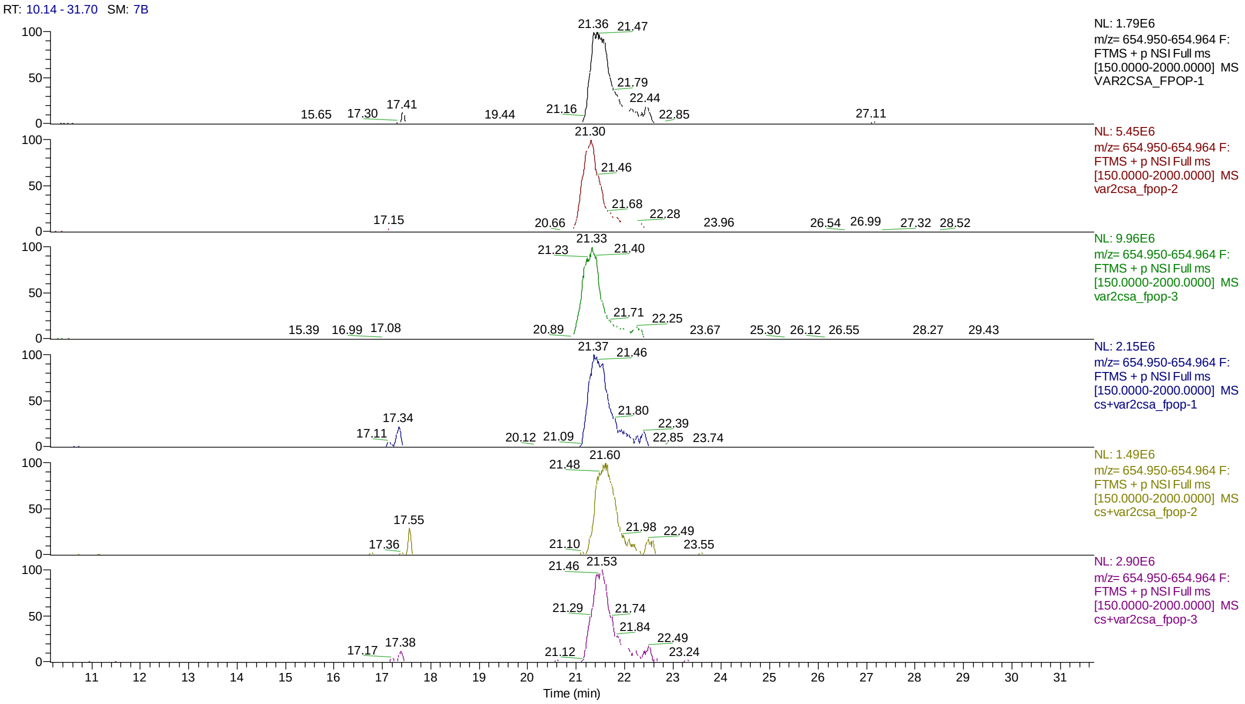

+1 ox

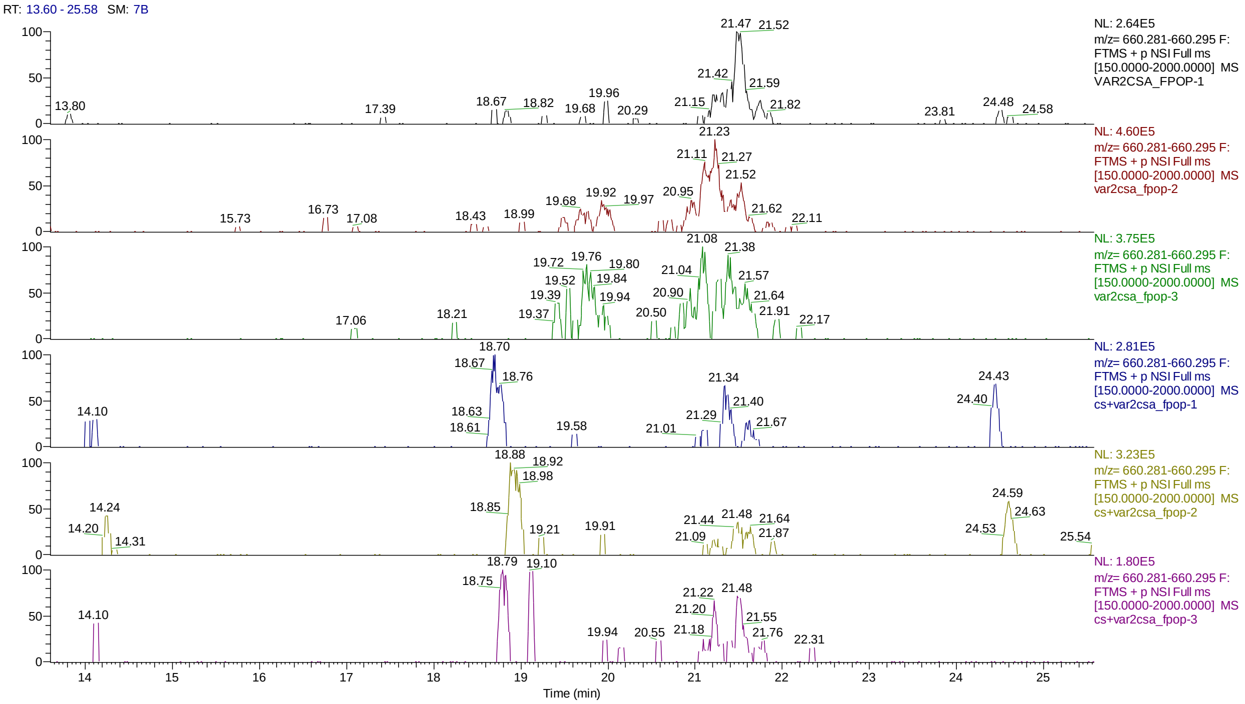

Y. ADLADIIRGTDQW [z=2] Position 160-172

Unoxidized peptide

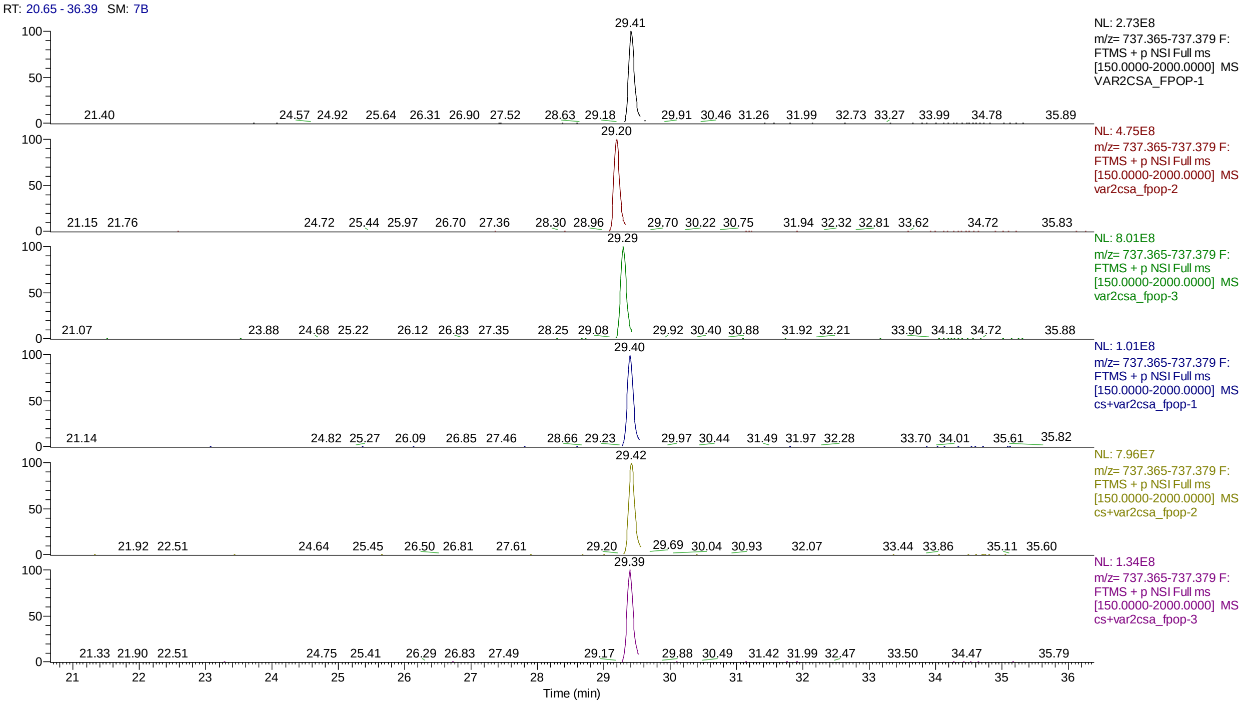

+1 ox

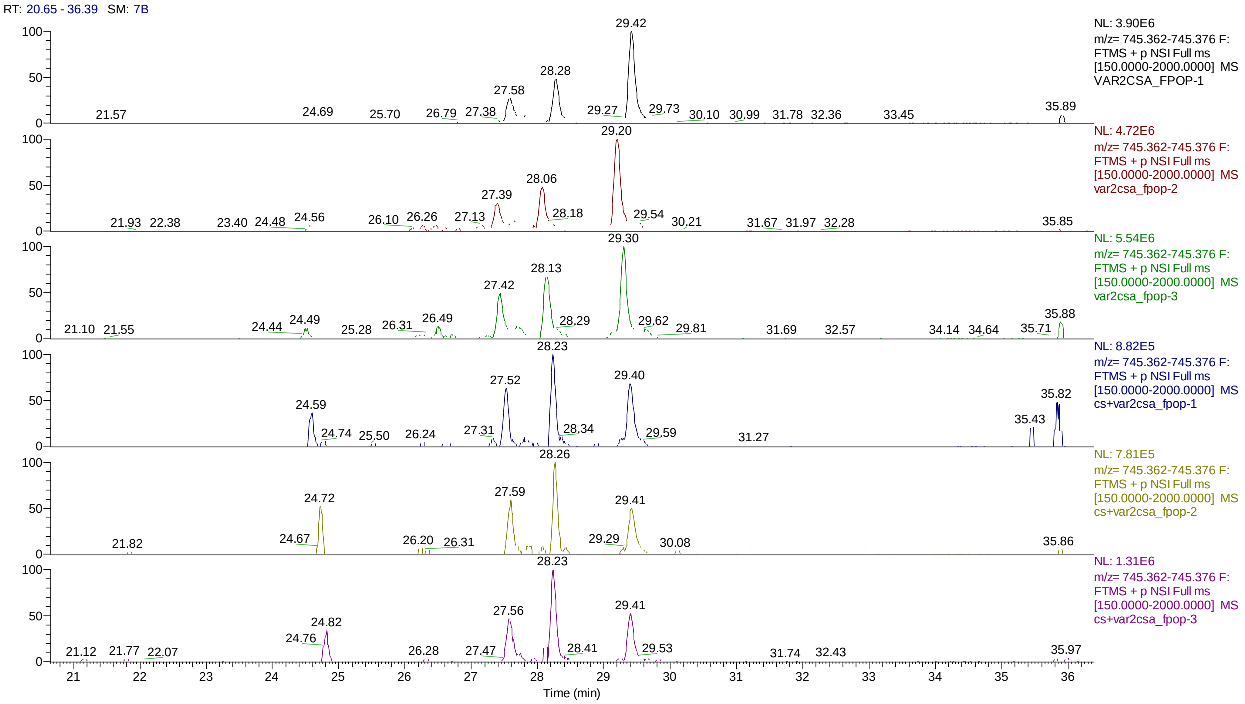

Z. RTSGKSDRKKNF [z=2] Position 242-253

Unoxidized peptide

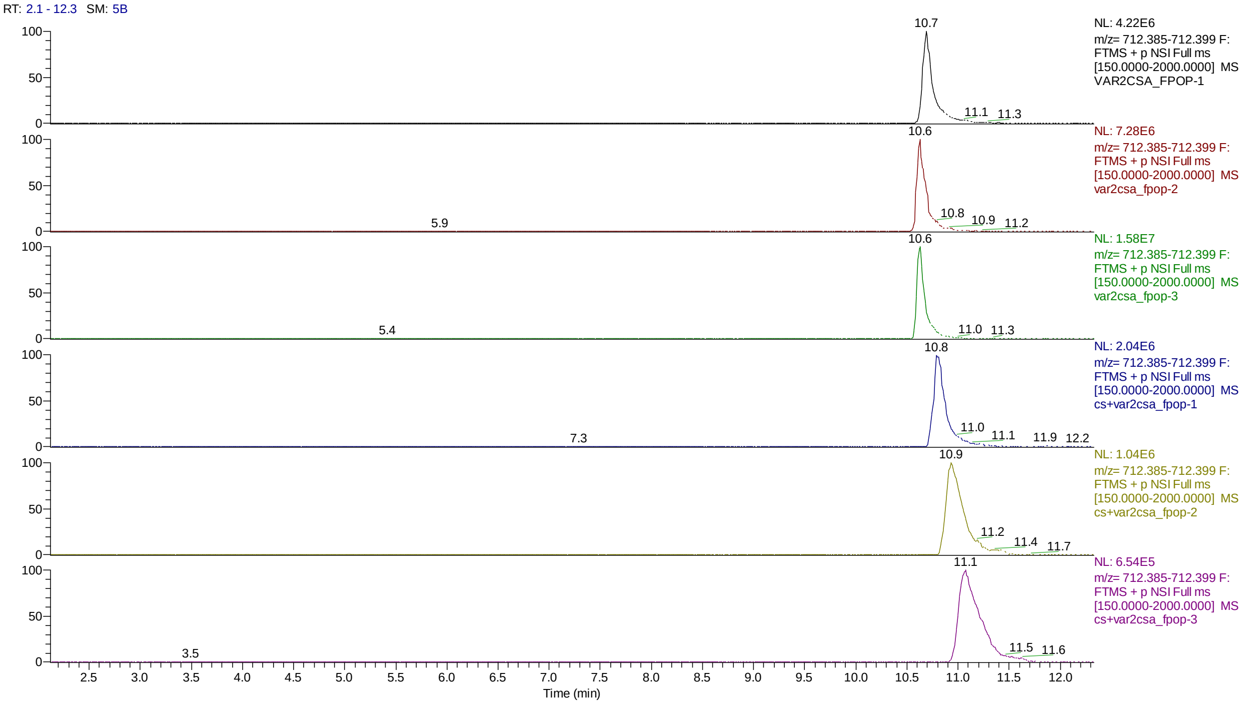

+1 ox

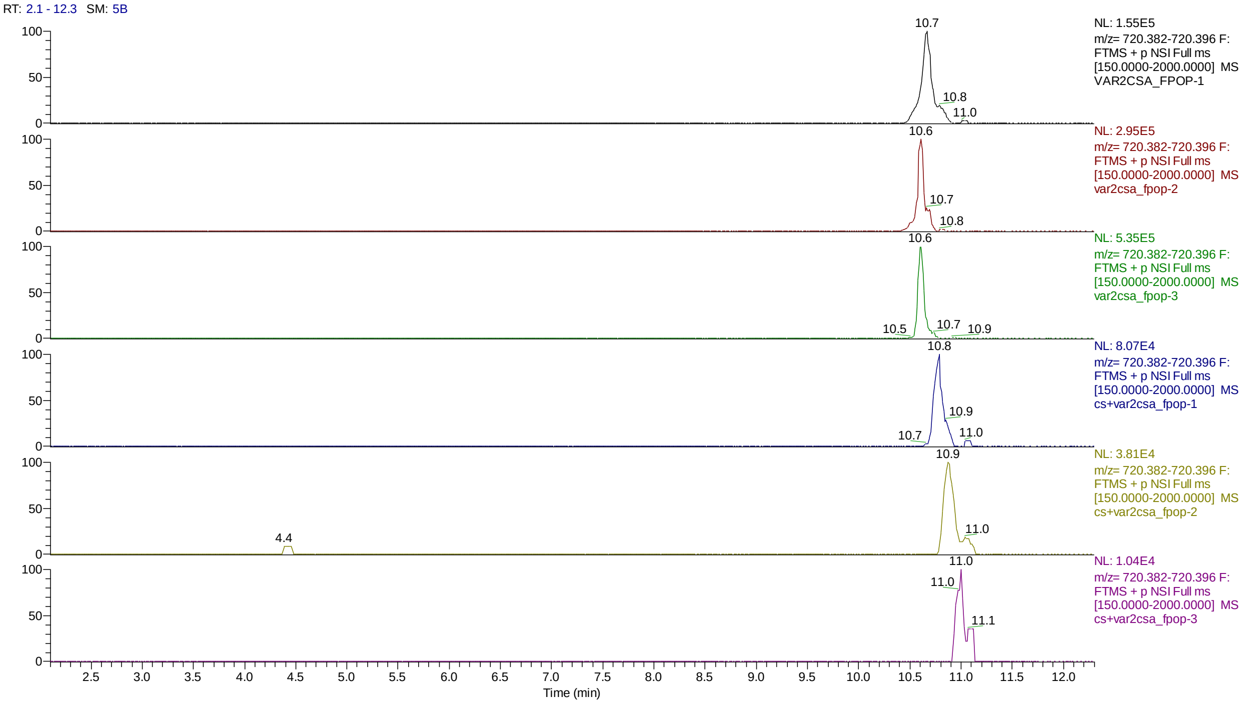

Fig S9: Extracted ion chromatograms of the unoxidized as well as major oxidation products for all FPOP oxidized VAR2CSA peptides. A-M) Identified VAR2CSA peptides significantly protected upon plCS binding. In each screenshot, the top three chromatograms are VAR2CSA in the absence of plCS and in the bottom three is peptides in the presence of plCS. +1 ox, +2 ox, +3 ox represents net addition of 1, 2 or 3 oxygen atoms, respectively. Where MS/MS data by CID allow the major site(s) of oxidation to be narrowed down, the residue or small stretch of residues are listed. Oxidized amino acids are identified when possible. N-O) Identified VAR2CSA peptides significantly exposed in the presence of plCS. Displayed as for A-O. P-Z) Identified VAR2CSA peptides not significantly changed in the presence of plCS. Displayed as for A-O.

Fig. S10: Annotated MS/MS spectra of [M+3H]3+ ion of peptide 543-558.

a

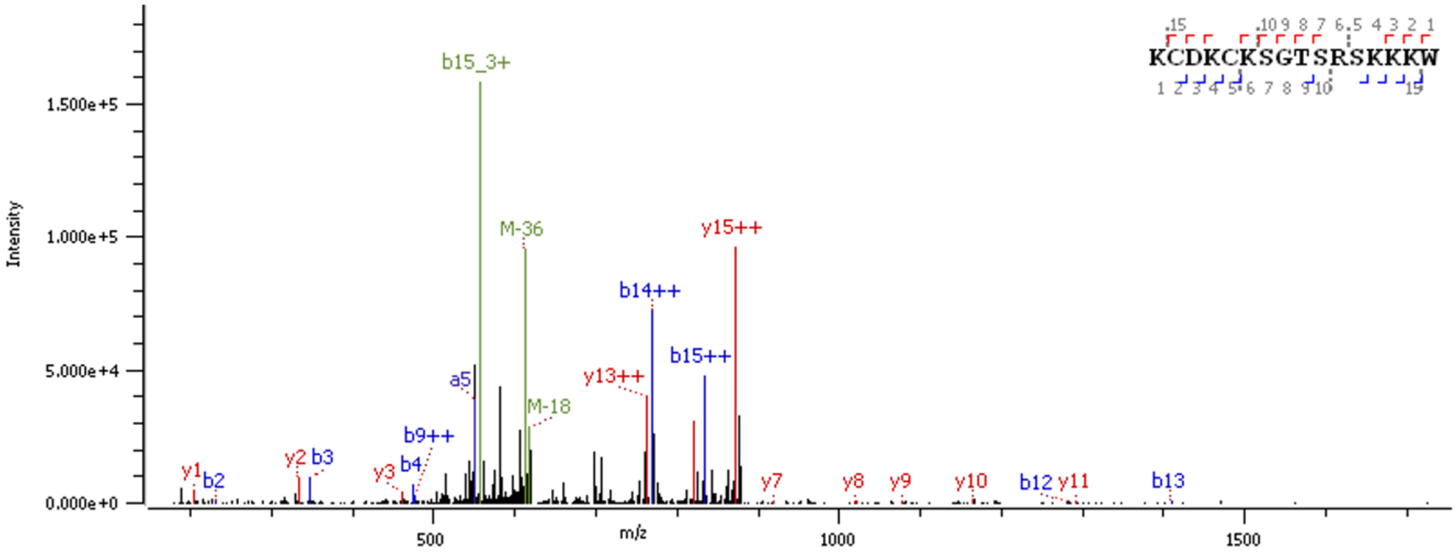

b

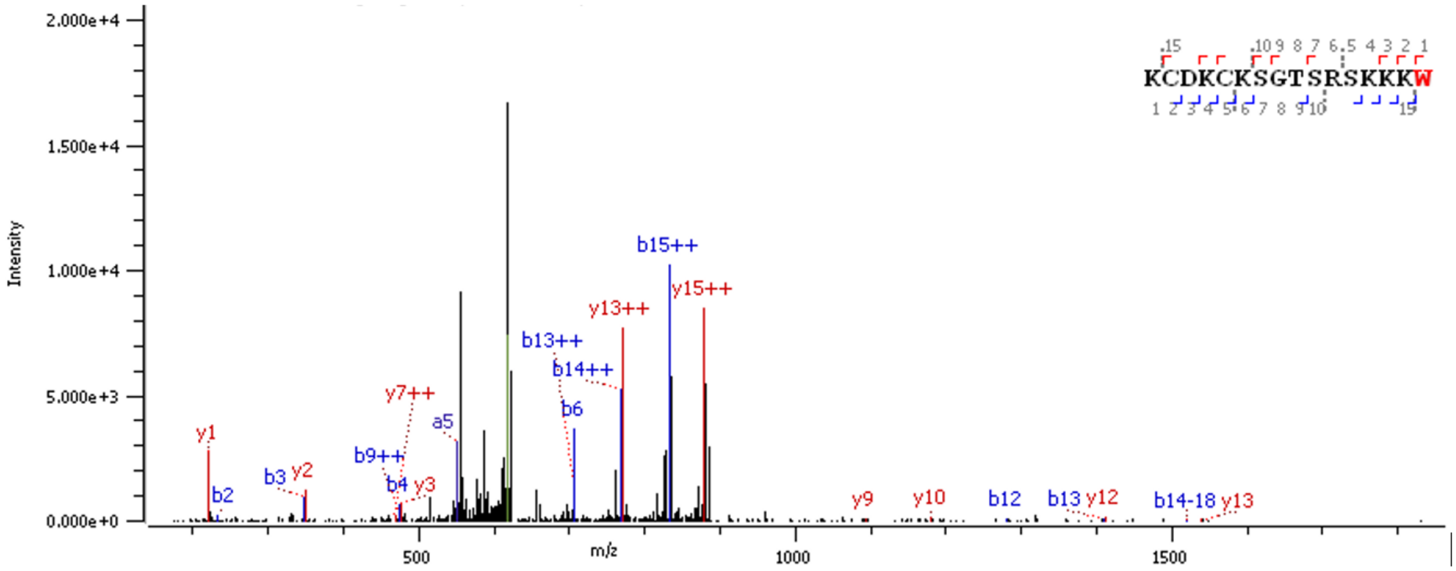

Fig S10: a) MS/MS of the unoxidized peptide. b) MS/MS of the +16 Da oxidized version of the same peptide. Product ion masses from the y-ion series clearly place the major oxidation site at W558.

Fig. S11: Domain flexibility by MD simulations

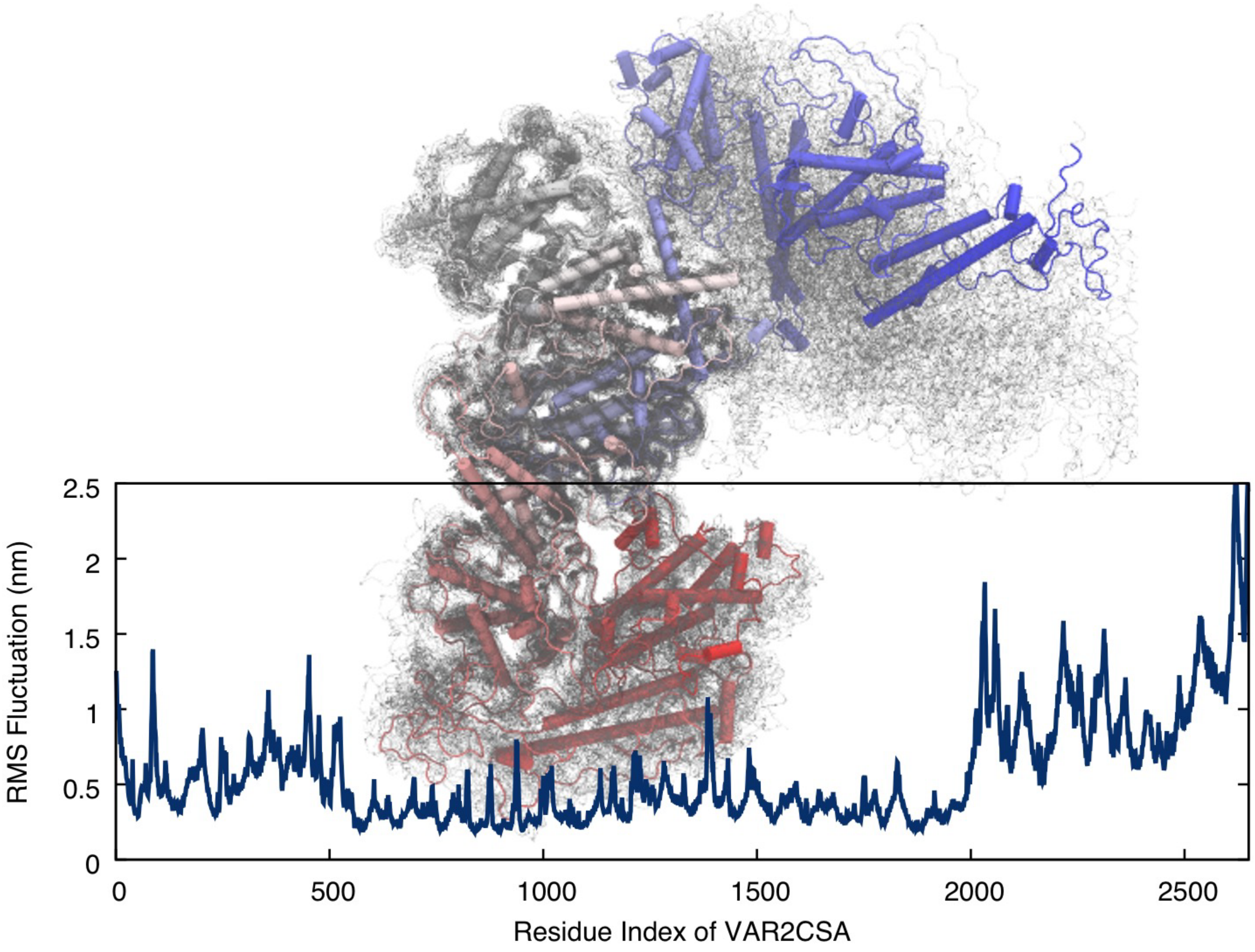

Fig S11: Domain flexibility revealed by MD simulations. The RMS fluctuation of VAR2CSA at a residue level revealed by the MD simulation of the VAR2CSA-CS complex. There are significant motions observed in DBL1/5/6 domains and also the loop regions as illustrated by the grey transparent tubes.

Fig. S12: Gating strategy

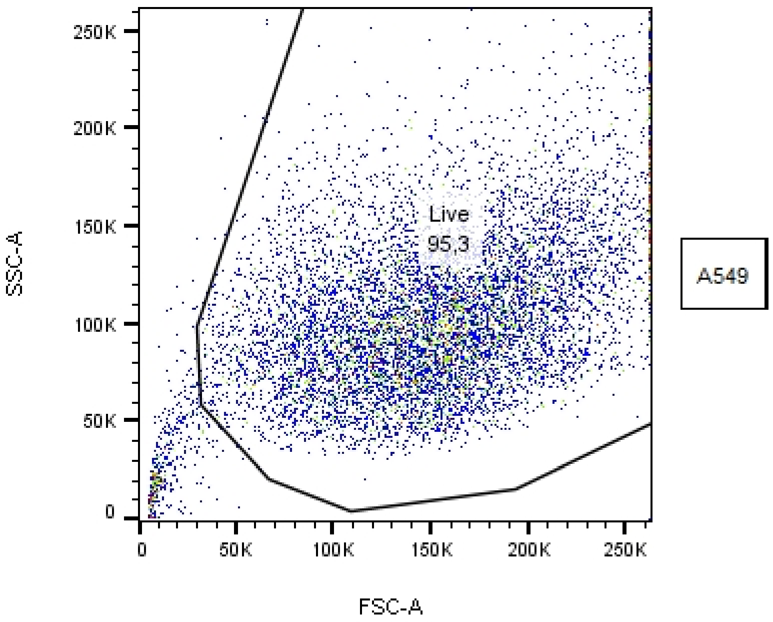

Fig S12: Gating strategy exemplifying how the A549 cells were gated based on forward and side scatter prior to calculating the geometric mean fluorescence intensity.

Table S1: CryoEM data collection, data processing and model building statistics

	**apo, DBL1-ID3**<br>**PDB 7B52, EMD-12017**	**with plCS, DBL1-ID3**<br>**PDB 7B54, EMD-12018**	**with plCS DBL5/6**<br>**PDB 7NNH, EMD-12477**
**Data collection**			
EM equipment	FEI Titan Krios	FEI Titan Krios	
Voltage (kV)	300	300	
Detecter	Falcon 3	Falcon 3	
Data collection mode	linear	counting	
Pixel size (Å)	0.83	0.83	
Electron dose (e-/Å2)	60	40	
Defocus range (mm)	-1.5 ~ -3.0	-1.5 ~ -3.0	
**Data processing**			
Software	cryosparc	cryosparc	cryosparc
Number of final used particles	102676	266774	97695
Symmetry	C1	C1	C1
Map resolution (Å)	3.8	3.1	3.9
**Model refinement statistics**			
Total built residues	1638	1732	637
Model-map-fit CC	0.84	0.82	0.79
**R.m.s.d.**			
bonds (Å)	0.004	0.005	0.004
angles (°)	0.84	0.767	1.364
**Molprobity statistics**			
Molprobity score	2.56	2.26	2.81
**Ramachandran Plot**			
Favored (%)	83.42	87.13	76.85
Allowed (%)	16.40	12.46	21.73
Rotamer outliers (%)	0.19	0.41	1.42
Clash score	23.94	13.54	35.39
Average B-factor ($Å^2$)	122.6	77.06	103.2

Table S2: Example of “Imbalance” Hetero-refinement runs for VAR2CSA apo structure showing particle numbers of Good and “Trash” classes and improved resolution.

	Initial particle number	Initial resolution(Å)	Low-pass filter resolution (Å)	Heterogenious refinement initial resolution (Å)	particle number (percentage)		Improved resolution (Å)
					Good class	"Trash" class	
Run 1	295833	4.33	40	20	220143 (74.4%)	75690 (25.6%)	4.11
Run 2	220143	4.11	40	20	188178 (85.5%)	12876 (14.5%)	4.06
Run 3	188178	4.06	20	10	147891 (78.6%)	40287 (21.4%)	3.90
Run 4	147891	3.90	20	10	117075 (79.2%)	30816 (20.8%)	3.84
Run 5	117075	3.84	20	10	102676 (87.7%)	14399 (12.3%)	3.82
