# Supplementary material for: Cryo-EM reveals the architecture of placental malaria VAR2CSA and provides molecular insight into chondroitin sulfate binding
Source: Nat Commun. 2021 May 19;12:2956. doi: 10.1038/s41467-021-23254-1 (PMC8134449; doi:10.1038/s41467-021-23254-1)
Supplement: Supplementary file 3 — Description of Additional Supplementary Files [file 41467_2021_23254_MOESM3_ESM.pdf]

Description of Additional Supplementary Files

File Name: Supplementary Movie 1
Description: A movie showing the electrostatic potential molecular surface of the full-length VAR2CSA modeled by MDFF. The electrostatic surface is colored according to their charge and isosurfaces drawn at 3.0 (blue) and −3.0 (red) electrostatic potential in units of kT/e.
