# Supplementary material for: Cryo-EM reveals the architecture of placental malaria VAR2CSA and provides molecular insight into chondroitin sulfate binding
Source: Nat Commun. 2021 May 19;12:2956. doi: 10.1038/s41467-021-23254-1 (PMC8134449; doi:10.1038/s41467-021-23254-1)
Supplement: Supplementary file 5 — Reporting Summary [file 41467_2021_23254_MOESM5_ESM.pdf]

nature research

Corresponding author(s): Ali Salanti, Pontus Gourdon

Last updated by author(s): Apr 12, 2021

Reporting Summary

Nature Research wishes to improve the reproducibility of the work that we publish. This form provides structure for consistency and transparency in reporting. For further information on Nature Research policies, see our Editorial Policies and the Editorial Policy Checklist.

Statistics

For all statistical analyses, confirm that the following items are present in the figure legend, table legend, main text, or Methods section.

n/a	Confirmed	
☐	☒	The exact sample size (*n*) for each experimental group/condition, given as a discrete number and unit of measurement
☒	☐	A statement on whether measurements were taken from distinct samples or whether the same sample was measured repeatedly
☐	☒	The statistical test(s) used AND whether they are one- or two-sided<br>*Only common tests should be described solely by name; describe more complex techniques in the Methods section.*
☒	☐	A description of all covariates tested
☒	☐	A description of any assumptions or corrections, such as tests of normality and adjustment for multiple comparisons
☐	☒	A full description of the statistical parameters including central tendency (e.g. means) or other basic estimates (e.g. regression coefficient) AND variation (e.g. standard deviation) or associated estimates of uncertainty (e.g. confidence intervals)
☐	☒	For null hypothesis testing, the test statistic (e.g. *F*, *t*, *r*) with confidence intervals, effect sizes, degrees of freedom and *P* value noted<br>*Give P values as exact values whenever suitable.*
☒	☐	For Bayesian analysis, information on the choice of priors and Markov chain Monte Carlo settings
☒	☐	For hierarchical and complex designs, identification of the appropriate level for tests and full reporting of outcomes
☒	☐	Estimates of effect sizes (e.g. Cohen's *d*, Pearson's *r*), indicating how they were calculated

Our web collection on statistics for biologists contains articles on many of the points above.

Software and code

Policy information about availability of computer code

Data collection	CryoEM data were collected using EPU software from FEI (https://www.thermofisher.com/dk/en/home/electron-microscopy/products/software-em-3d-vis/epu-software.html).
Data analysis	As described in Materials and Methods, the following software has been used:<br>CryoEM data processing was done with Cryosparc v 2.15.0(https://cryosparc.com/).<br>Model building was done with Coot (version 0.8.9.2) (https://www2.mrc-lmb.cam.ac.uk/personal/pemsley/coot/) and phenix.real_space_refinement version 1.18 (https://www.phenix-online.org/documentation/reference/real_space_refine.html).<br>Figure was made with Pymol version 2.3.4 (https://pymol.org/), Chimera version 1.16 (https://www.cgl.ucsf.edu/chimera/) and ChimeraX version 1.2 (https://www.cgl.ucsf.edu/chimerax/).<br>MD-simulation was done using Gromacs2019 (http://www.gromacs.org/). Atomistic MD simulation of the complex was prepared using CHARMMGUI version 3.2 webserver (http://charmm-gui.org/).<br>Molecular docking of CS to VAR2CSA was done using HADDOCK 2.4 (https://wenmr.science.uu.nl/haddock2.4/).<br>Attana QCM biosensor: Frequency data was measured using Attester software, the obtained data was fitted using TraceDrawer version 1.8.1 from Ridgeview Instruments AB. Raw data is available in source data file.<br>NanoDSF: Nanotemper prometheus NT.48 instrument and software was used to generate melting curves. Raw data is available in source data file.<br>FPOP-MS: Peptide analyses was done using Byonic version 2.10.5 (Protein metrics). Data was performed in triplicate for statistical analysis. Raw data is available in source data file.<br>Flow cytometry: Results were analysed using FlowJo software version 10.5.3 (BD life sciences). Raw data is available in source data file.<br>Sequence LOGOs were made using WebLogo3.

For manuscripts utilizing custom algorithms or software that are central to the research but not yet described in published literature, software must be made available to editors and reviewers. We strongly encourage code deposition in a community repository (e.g. GitHub). See the Nature Research guidelines for submitting code & software for further information.

Data

Policy information about availability of data

All manuscripts must include a data availability statement. This statement should provide the following information, where applicable:

- Accession codes, unique identifiers, or web links for publicly available datasets
- A list of figures that have associated raw data
- A description of any restrictions on data availability

The following accession codes and identifiers are used in the work:
Amino acid sequence of VAR2CSA extracellular fragment from genotype FCR3: Genbank GU249598
Cryo-EM: VAR2CSA DBL1-ID3 apostructure: EMD-12017 and PDB-ID 7B52
Cryo-EM: VAR2CSA:CS complex structure DBL1-ID3: EMD-12018 and PDB-ID 7B54
Cryo-EM: VAR2CSA:CS complex structure DBL5-DBL6: EMD-12477 and PDB-ID 7NNH
LC/MS FPOP data: ProteomeXchange server accession number PXD02154
VAR2 sequences for alignment analysis were extracted from the var database, varDB PF3K (vardb.org).

Field-specific reporting

Please select the one below that is the best fit for your research. If you are not sure, read the appropriate sections before making your selection.

- [x] Life sciences
- [ ] Behavioural & social sciences
- [ ] Ecological, evolutionary & environmental sciences

For a reference copy of the document with all sections, see nature.com/documents/nr-reporting-summary-flat.pdf

Life sciences study design

All studies must disclose on these points even when the disclosure is negative.

Sample size	Complete Cryo-EM statistics are indicated in the method section, table S1-S2 and figure S5-S6
Data exclusions	Complete Cryo-EM statistics are indicated in the method section, table S1-S2 and figure S5-S6
Replication	VAR2CSA ectodomain was expressed and purified multiple times. Multiple Cryo-EM grids were prepared and frozen, data was collected for one selected grid of the apostructure and one grid of the VAR2CSA:plCS complex
Randomization	Extracted particles were randomly assigned to calculate gold-standard FSC
Blinding	No blinding was applied

Reporting for specific materials, systems and methods

We require information from authors about some types of materials, experimental systems and methods used in many studies. Here, indicate whether each material, system or method listed is relevant to your study. If you are not sure if a list item applies to your research, read the appropriate section before selecting a response.

Materials & experimental systems

n/a	Involved in the study	
[ ]	[x]	Antibodies
[ ]	[x]	Eukaryotic cell lines
[x]	[ ]	Palaeontology and archaeology
[x]	[ ]	Animals and other organisms
[x]	[ ]	Human research participants
[x]	[ ]	Clinical data
[x]	[ ]	Dual use research of concern

Methods

n/a	Involved in the study	
[x]	[ ]	ChIP-seq
[ ]	[x]	Flow cytometry
[x]	[ ]	MRI-based neuroimaging

Antibodies

Antibodies used	V5-tag Monoclonal Antibody, FITC (Invitrogen 46-0308)
Validation	Highly specific and sensitive antibody for detection of V5-tagged proteins by direct binding to the following amino acid seq: GKPIPNPLLGLDST. The antibody has been validated by testing binding against Positope(TM) control protein.

Eukaryotic cell lines

Policy information about cell lines

Cell line source(s)	The A549 cell line used was kindly provided by Peter Holst who procured it from AmsBio
Authentication	Cell line was not authenticated
Mycoplasma contamination	Mycoplasma contamination was tested on a regular basis. Cells were discarded if tested positive
Commonly misidentified lines (See ICLAC register)	A549 is not on the list of misidentified cells from ICLAC, but has been identified as a contaminating cell line of JHU028

Flow Cytometry

Plots

Confirm that:

- [x] The axis labels state the marker and fluorochrome used (e.g. CD4-FITC).
- [x] The axis scales are clearly visible. Include numbers along axes only for bottom left plot of group (a 'group' is an analysis of identical markers).
- [x] All plots are contour plots with outliers or pseudocolor plots.
- [x] A numerical value for number of cells or percentage (with statistics) is provided.

Methodology

Sample preparation	The A549 cell line was grown and maintained in DMEM media with GlutaMax(TM) (Gibco) supplemented with 10% (v/v) FBS and 1% (v/v) penicillin-streptomycin. The cell line was sustained at 5% CO2 and 37C and passaged by detaching with Trypsin-EDTA solution 1. Proteins were diluted in PBS + 2% (v/v) FBS.
Instrument	LSR-II flow cytometer (BD)
Software	FlowJo software version 10.5.3 (BD life sciences)
Cell population abundance	Analysis was done using a pure cell isolate of A549 cells. Based on forward/side scatter a cell count 95% was used for the analysis.
Gating strategy	A549 cells were gated based on forward and side scatter prior to calculating the geometric mean fluorescence intensity, mainly removing cell debris. Figure S12 shows the gating strategy used.

- [x] Tick this box to confirm that a figure exemplifying the gating strategy is provided in the Supplementary Information.
